# SILAC-based phosphoproteomics reveals new PP2A-Cdc55-regulated processes in budding yeast

**DOI:** 10.1093/gigascience/giy047

**Published:** 2018-05-24

**Authors:** Barbara Baro, Soraya Játiva, Inés Calabria, Judith Vinaixa, Joan-Josep Bech-Serra, Carolina de LaTorre, João Rodrigues, María Luisa Hernáez, Concha Gil, Silvia Barceló-Batllori, Martin R Larsen, Ethel Queralt

**Affiliations:** 1 Cell Cycle Group, Cancer Epigenetics and Biology Program, Institut d'Investigacions Biomèdica de Bellvitge (IDIBELL), L'Hospitalet de Llobregat, Barcelona, Spain; 2 IDIBELL Proteomics Unit, Institut d'Investigacions Biomèdica de Bellvitge, L'Hospitalet de Llobregat, Barcelona, Spain; 3 Structural Biology Department, School of Medicine, Stanford, California, USA; 4 Proteomics Unit, Parque Científico de Madrid, Facultad de Farmacia, Universidad Complutense de Madrid, Madrid, Spain; 5 Department of Biochemistry and Molecular Biology, Odense M, Denmark

**Keywords:** mitosis, PP2A^Cdc55^ phosphatase, Pkc1; mitotic exit network (MEN), Mob1, phosphoproteomics, SILAC

## Abstract

**Background:**

Protein phosphatase 2A (PP2A) is a family of conserved serine/threonine phosphatases involved in several essential aspects of cell growth and proliferation. PP2A^Cdc55^ phosphatase has been extensively related to cell cycle events in budding yeast; however, few PP2A^Cdc55^ substrates have been identified. Here, we performed a quantitative mass spectrometry approach to reveal new substrates of PP2A^Cdc55^ phosphatase and new PP2A-related processes in mitotic arrested cells.

**Results:**

We identified 62 statistically significant PP2A^Cdc55^ substrates involved mainly in actin-cytoskeleton organization. In addition, we validated new PP2A^Cdc55^ substrates such as Slk19 and Lte1, involved in early and late anaphase pathways, and Zeo1, a component of the cell wall integrity pathway. Finally, we constructed docking models of Cdc55 and its substrate Mob1. We found that the predominant interface on Cdc55 is mediated by a protruding loop consisting of residues 84–90, thus highlighting the relevance of these aminoacids for substrate interaction.

**Conclusions:**

We used phosphoproteomics of Cdc55-deficient cells to uncover new PP2A^Cdc55^ substrates and functions in mitosis. As expected, several hyperphosphorylated proteins corresponded to Cdk1-dependent substrates, although other kinases’ consensus motifs were also enriched in our dataset, suggesting that PP2A^Cdc55^ counteracts and regulates other kinases distinct from Cdk1. Indeed, Pkc1 emerged as a novel node of PP2A^Cdc55^ regulation, highlighting a major role of PP2A^Cdc55^ in actin cytoskeleton and cytokinesis, gene ontology terms significantly enriched in the PP2A^Cdc55^-dependent phosphoproteome.

## Introduction

Protein phosphorylation is a key regulatory mechanism of protein function that governs cell cycle progression (reviewed in [1]). The highly conserved and specific family of cyclin-dependent serine/threonine kinases, the Cdks, were considered the main component of the cell cycle control system once they were discovered. Currently, it has become clear that the opposing phosphatases also play a key role in setting the net phosphorylation state of each substrate, thereby being the other side of the coin controlling phosphorylation waves during cell cycle progression. Cdk1-cyclin activity progressively increases as the cell cycle progresses, reaching its maximum in metaphase. At the end of mitosis, high Cdk1 activity needs to return to lower levels to enter into a new G1 phase, and activation of Cdk1-counteracting phosphatases is required for this transition.

Type 2A phosphatases (PP2A) is a family of conserved protein serine/threonine phosphatases involved in several essential aspects of cell growth and proliferation. PP2A is a major Cdk1-counteracting phosphatase during cell cycle progression, which works solely as a multimeric enzyme [2]. The PP2A core enzyme consists of a scaffold subunit and a catalytic subunit. The heterodimeric complex interacts with a variable regulatory subunit (B subunit) to assemble into a holoenzyme. Although highly conserved within the same family, these regulatory subunits share little sequence similarity across families, and their expression levels vary greatly in different cell types and tissues [3]. Several studies have shown that PP2A regulatory subunits confer exquisite substrate specificity to PP2A holoenzymes *in vivo* [4–12].

PP2A is highly conserved from yeast to humans. Knockdown of either the catalytic or a subset of regulatory subunit genes of PP2A holoenzymes results in unviable cells [13–17]. In *Saccharomyces cerevisiae*, the PP2A scaffold subunit is known as Tpd3. The catalytic subunit of the core enzyme is either Pph21 or Pph22, two highly homologous proteins sharing 95% sequence identity [18, 19]. Mutation of both *PPH21* and *PPH22* eliminates the majority of PP2A activity in the cell and drastically reduces growth. Strains lacking *PPH21*, *PPH22*, and a third related gene, *PPH3*, are completely unviable [19]. The regulatory subunits comprise Cdc55 (B-type in vertebrates), Rts1 (B’-type in vertebrates), and the predicted B-subunit Rts3. In this work, we refer to Tpd3, Pph21 or Pph22, and Cdc55 holoenzyme as PP2A^Cdc55^.

PP2A^Cdc55^ and its mammalian homolog, PP2A^B55^, have been extensively studied for their role in mitotic entry regulation (reviewed in [20]). The regulatory axis of Greatwall and PP2A inhibitors, endosulfins (Igo1/2 in budding yeast), govern mitotic entry in both yeast and higher eukaryotes [21–24], illustrating the strong conservation of PP2A regulatory mechanisms across eukaryotes. One of the first known functions of PP2A^Cdc55^ in cell-cycle regulation was its key role affecting Swe1 and Mih1 activity at the G2/M transition [25–30] (Wee1 and Cdc25 in vertebrates). More recently, signals regarding the status of membrane traffic have been shown to be integrated into mitosis progression through PP2A^Cdc55^ via a signaling cascade that includes Rho1, Pkc1, and Zds1/2. Pkc1 binds to PP2A^Cdc55^-Zds1/2, which directly controls the phosphorylation states of Mih1 and Swe1 [31–36].

However, PP2A^Cdc55^ substrates and functions during mitotic exit are less understood, since another phosphatase, Cdc14, which is essential and specifically activated at anaphase onset, has been considered the principal Cdk1-counteracting phosphatase during mitotic exit in budding yeast. In contrast, in vertebrate cells, although *CDC14* homologues are present [37], their functions seem less conserved [38], and PP2A-B55 and PP1 phosphatases are considered the major Cdk1-counteracting phosphatases during mitotic exit [39, 40]. Indeed, yeast PP2A^Cdc55^ has also been shown to play a major role during mitotic exit. PP2A^Cdc55^ counteracts Cdk1-dependent phosphorylation of Net1, which is crucial for Net1-Cdc14 dissociation [8]. Zds1/2 proteins cooperate with separase to downregulate PP2A^Cdc55^ at anaphase onset [41, 42], which leads to Cdc14 activation and release. Thus, Zds1/2 are common PP2A^Cdc55^ modulators, participating in both entry and exit from mitosis. It has recently been described that PP2A^Cdc55^ downregulation in anaphase also initiates the Mitotic Exit Network (MEN) by dephosphorylating the MEN components Bfa1 and Mob1 [43]. In addition, PP2A^Cdc55^ downregulation at anaphase-onset facilitates separase proteolytic activity towards Scc1, which triggers sister-chromatid segregation [44]. Finally, PP2A^Cdc55^ as well as its homologue, PP2A-B55, has been shown to counteract Cdk1-dependent phosphorylation of APC/C during mitosis [45–48]. In conclusion, PP2A^Cdc55^ is also a major Cdk1-counteracting phosphatase during mitotic exit in budding yeast.

Quantitative mass spectrometry analysis has been used to identify Cdk-dependent phosphorylation sites in a large number of substrates *in vivo* by comparing the phosphoproteome of wild-type cells and Cdk1 defective cells [49, 50]. More recently, a global analysis of Cdc14 dephosphorylation sites was performed using a similar approach [51, 52]. In this study, we performed a systematic quantitative phosphoproteomic analysis of PP2A^Cdc55^- deficient cells to identify novel PP2A^Cdc55^ substrates and regulated processes. Since drug inhibition by Okadaic acid in budding yeast only works at high concentration, which also inhibits other Ser/Thr phosphatases, and due to the specificity that the regulatory subunits confer to PP2A [53], in our approach we used a *cdc55* deletion mutant to explore the PP2A^Cdc55^-dependent phosphoproteome. Hence, *cdc55*- deficient cells lack PP2A^Cdc55^ activity but not the other PP2A complexes, PP2A^Rts1^ or PP2A^Rts3^. We identified both known and potentially new substrates for PP2A^Cdc55^ as well as their phosphorylation sites. Our dataset is consistent with PP2A^Cdc55^ being a serine/threonine phosphatase and having a major role in counteracting Cdk1 activity, since S/T-P sites were the most abundant motif enriched in the absence of Cdc55. But, interestingly, we also identified other kinase consensus sequences corresponding to Pro-directed kinases, GSK3 kinases, Cdc5 Polo kinase, and casein kinases I and II, suggesting that PP2A^Cdc55^ counteracts other kinases apart from Cdk1 and/or regulates their activities. Finally, we were able validate up to 9 targets by protein-protein interactions and/or by western blot, which strongly support the validity of our study. We assume that the substrates of the PP2A^Cdc55^ phosphatase identified might not be all direct targets; however, as well as this, our work also uncovered valuable new PP2A-related processes.

## Data Description

To screen for potential new substrates of the PP2A^Cdc55^ phosphatase during mitosis, we performed a quantitative phosphoproteomic analysis based on the Stable Isotope Labelling by Amino Acids in Cell Culture (SILAC) technique. To study the PP2A^Cdc55^-dependent phosphoproteome, we compared the phosphoproteome of a wild-type strain and a *cdc55Δ* mutant strain, which lacks the activity of PP2A^Cdc55^ but not other PP2A complexes. The PP2A regulatory subunits confer substrate specificity to PP2A. Therefore, in our approach we specifically studied the PP2A^Cdc55^ and no other PP2A complexes (with Rts1 or Rts3). To minimize compensatory mutations that might accumulate over time in the gene deletion strain, we freshly prepared the *cdc55Δ* mutant. Wild-type and *cdc55Δ* cells were grown in methionine-free minimum media containing ^13^C_6_-lysine and -arginine (heavy) or unmodified arginine and lysine (light), respectively. Both strains expressed *CDC20* under the control of the repressible *MET3* promoter and were synchronized at the metaphase-to-anaphase transition by adding methionine to the media, which causes Cdc20 depletion. At the time of harvesting, more than 95% of cells in each culture were arrested in metaphase. Protein extracts were prepared as described in Methods.

**Figure 1:**
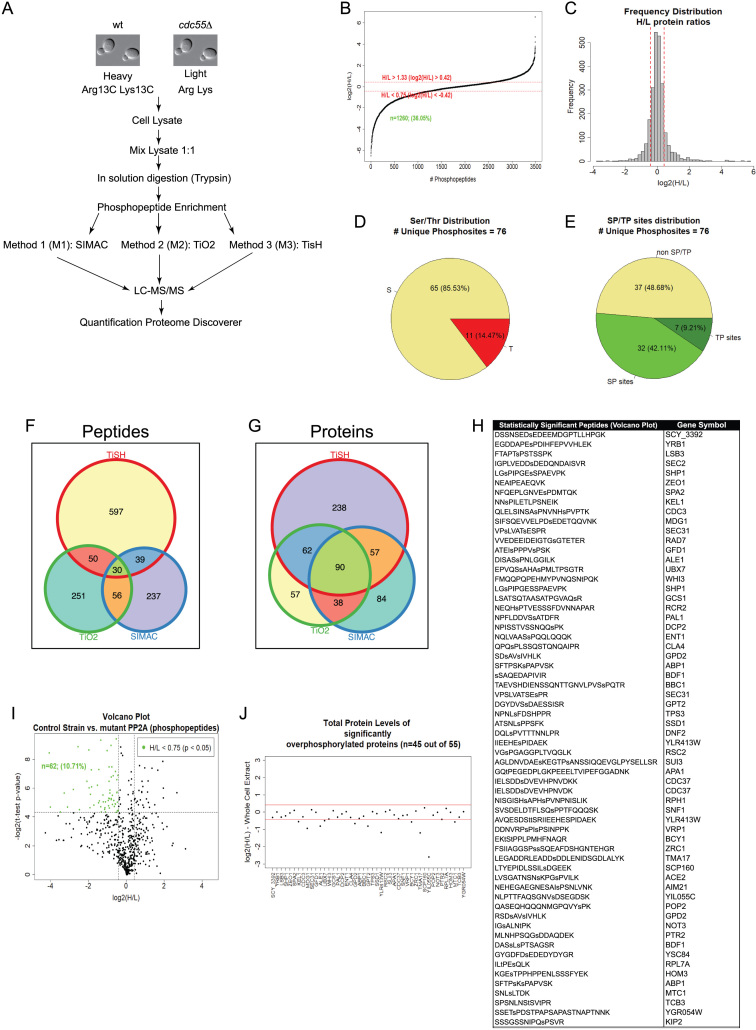
Potential substrates of PP2A^Cdc55^ phosphatase. A) Scheme of the three phosphoenrichment approaches performed in our phosphoproteome study. B) The normalized heavy/light (H/L) ratio of all phosphopeptides. The number of phosphopeptides (n = 1260) with H/L ratios <0.75 (corresponding to the hyperphosphorylated peptides) is shown. C) Frequency distribution of the H/L ratios from an aliquot of the whole protein extracts before phosphopeptide enrichment. The protein abundance is unchangeable for most of the peptides. Red lines mark the lower and upper limits, which are set to 0.75 (log2 = −0.42) and 1.3 (log2 = 0.42), respectively. D) Distribution of the Ser, Thr, and Tyr residues among the 62 hyperphosphorylated peptides statistically significant (Fig. 1I) in the *cdc55Δ* mutant. All the peptides (10,069) identified in our three SILAC approaches were used as background. E) Distribution of the S/TP sites within the 62 hyperphosphorylated peptides. F-G) Venn diagrams representing overlapping hits from the three approaches for both hyperphosphorylated peptides and proteins. H) A list of the 62 hyperphosphorylated peptides statistically significant from Fig. 1I. I) Volcano plot representing the common phosphopeptides (present in at least 2 out of 3 approaches) generated from two-tailed Student's *t* test (*P* <0.05). Green dots represent the significant proteins. J) Analysis of the protein abundance of the 55 common proteins from I.

We used three different strategies to enrich for phosphopeptides: SIMAC, TiO_2_, and TiSH-based (TiO_2_-SIMAC-HILIC). A schematic representation of the different strategies used is shown in Fig. 1A (more details in Additional file 1 and Methods). Different phosphopeptide enrichment methods isolated distinct, partially overlapping segments of a phosphoproteome, whereas none of the methods were able to provide a whole phosphoproteome [54, 55]. Phosphopeptide enrichment strategies are complementary, such that a combination of methods greatly enhances the number of phosphopeptides isolated from complex samples [56]. Analysis of the heavy/light labeled phosphopeptides was performed by LC-tandem mass spectrometry (MS/MS) (see Methods for more details). Global analysis of the data led to the identification of 10,069 peptides (Additional file 2), including 4,467 phosphopeptides. Only peptides identified with high confidence (<1% FDR) were used for further analysis. The mass spectrometry proteomics data have been deposited to the ProteomeXchange Consortium with the dataset identifier PXD007613.

## Analyses

### Large-scale identification of PP2A^Cdc55^-dependent phosphoproteome in metaphase-arrested cells

To study the PP2A^Cdc55^-dependent phosphoproteome, we selected the hyperphosphorylated peptides averaging all the single phosphopeptides obtained in the three experimental approaches (SIMAC, TiO2, and TiSH), according to the filtering parameters described in methods (Fig. 1B; H/L ratio <0.75 or log2(H/L) ratio <-0.42). Analysis of this subset of data led to the quantification of 1,491 phosphoproteins, represented by 4,467 phosphopeptides. Among them, we found 1,260 hyperphosphorylated peptides that show H/L ratios <0.75 (log2(H/L)<-0.42), corresponding to 628 phosphoproteins. The hyperphosphorylated peptides selected and statistical parameters used are shown in Additional file 3. In addition, already known PP2A^Cdc55^ substrates such as Net1, Mob1, Gis1, and Whi5 were identified as being hyperphosphorylated in the *cdc55∆* mutant, which strongly supports the validity of our approach [8, 43, 57, 58].

Since phosphorylation changes measured by the heavy/light ratio could be affected by changes in protein abundance due to absence of Cdc55, we analyzed one aliquot of the protein extract without phosphopeptide enrichment (see Methods) and determined the heavy/light ratio to account for protein abundance. A full list of the peptides and proteins identified is summarized in Additional file 4. We could quantify a total of 18,592 peptides, of which 15,640 peptides contained a heavy/light ratio >0.8 and 2,952 peptides that had a heavy/light ratio <0.8. Therefore, only 15.8% of the peptides had reduced protein abundance due to the absence of Cdc55 (Fig. 1C). In fact, we identified 286 matching proteins to the selected hyperphosphorylated dataset (see Additional file 5), and most of them had similar protein abundance between the wild type and the *cdc55∆* mutant (heavy/light ratio >0.8 in non-enriched analysis). Therefore, we conclude that most of the hyperphosphorylated proteins selected with a heavy/light ratio <0.75 (log2(H/L) ratio <-0.42) correspond to phosphorylation changes and not to protein abundance changes. Nevertheless, we cannot rule out that, for some proteins, changes in protein abundance might affect the heavy/light ratio, since we could not identify all the hyperphosphorylated peptides in the non-enriched fraction.

The overlap of hyperphosphorylated peptides (n = 1,260) and their corresponding phosphoproteins found in the three different approaches (SIMAC, TiO2, and TiSH) are shown by Venn diagrams (Fig. 1F–G). Common proteins and peptides found in the three experiments are shown in Additional file 6. The volcano plot of the common phosphopeptides (at least in two of the three approaches) showed a higher amount of hyperphosphorylated peptides (n = 62, corresponding to 55 proteins) compared to the hypophosphorylated ones (n = 27) (Fig. 1I), in accordance with enrichment in PP2A^Cdc55^ potential substrates. We managed to identify and quantify the amount of protein of 45 proteins (out of 55) in the whole cell extract (Fig. 1J). In 75% (34/45) of the cases, the amount of protein did not change significantly and, therefore, we can be certain that the H/L ratio is due to hyperphosphorylation of the peptides rather than a change in protein abundance. A list of the 62 statistically significant phosphopeptides is shown in Fig. 1H and Additional file 7.

We next analyzed the phosphorylated residues found in the 62 hyperphosphorylated peptides using the non-enriched sample as background. From 76 unique phosphosites identified, 85.53% corresponded to phosphoserine and 14.47% to phosphothreonine (Fig. 1D), which is consistent with PP2A^Cdc55^ being a Ser/Thr phosphatase.

### PP2A^Cdc55^- dependent phosphorylation sites of known kinases

We were interested in studying the kinases counteracted by PP2A^Cdc55^. It has been shown that PP2A^Cdc55^ phosphatase can counteract Cdk1 phosphorylation [8] and Cdc5 phosphorylation [43, 44]. We found that 51.32% of the phosphosites correspond to SP/TP (minimum Cdk1 consensus sequence), consistent with PP2A^Cdc55^ mainly counteracting Cdk1 phosphorylation (Fig. 1E).

**Figure 2:**
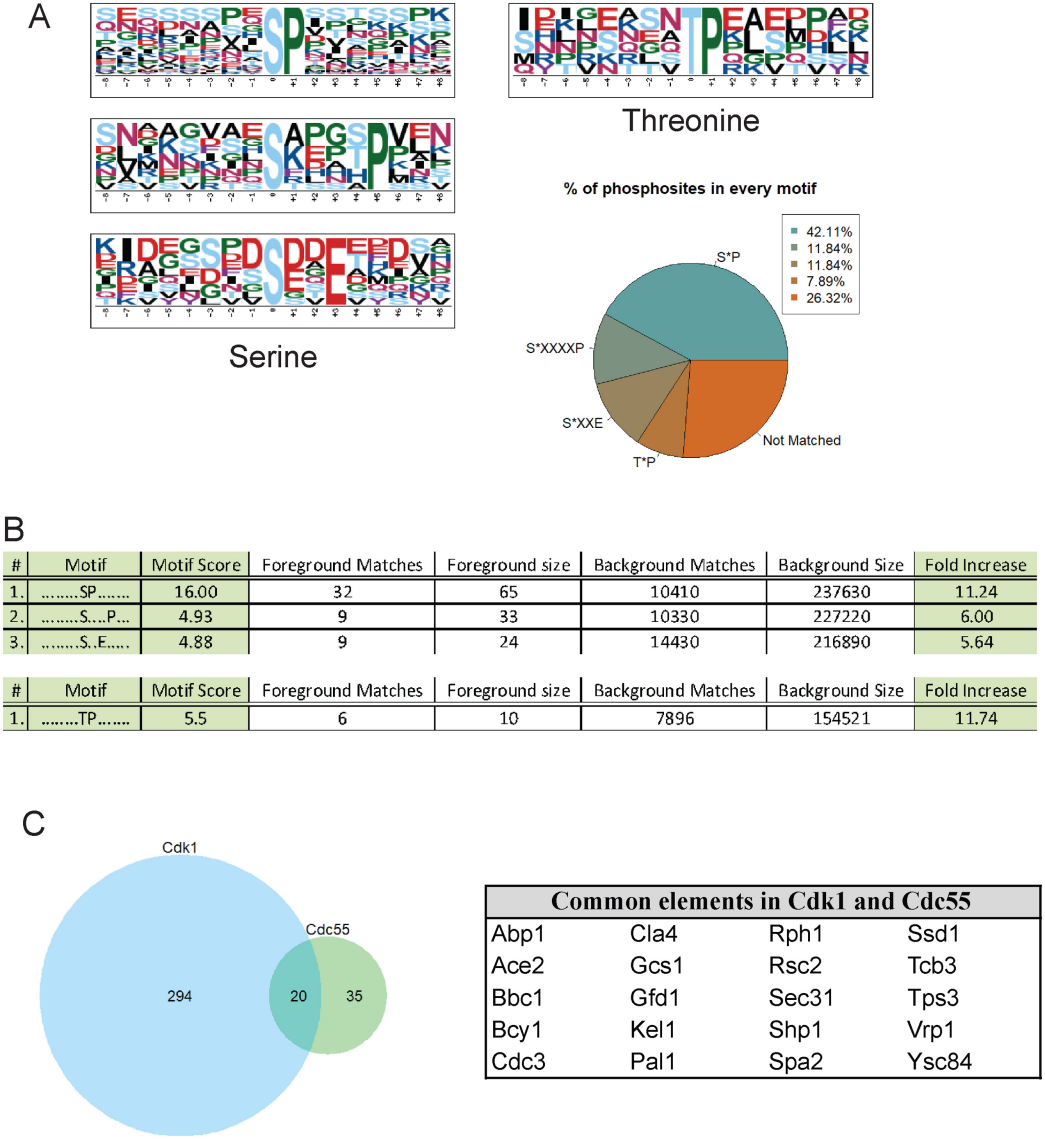
Consensus phosphorylation sites found hyperphosphorylated in absence of PP2A^Cdc55^. A) Motifs logo found using Motif-X for either central residue phospho-Serine or phospho-Threonine. B) Phosphomotif consensus sequence, motif score, and fold increase for each consensus motif. C) Common elements between Cdk1 and PP2A^Cdc55^ targets. Venn diagrams from the common Cdk1-PP2A^Cdc55^ targets and common protein targets are shown.

To identify consensus phosphorylation sites of other known protein kinases, enriched sequence motifs surrounding the phosphosites in the 62 hyperphosphorylated peptides were analyzed via Motif-X [59]. The 62 unique hyperphosphorylated peptides contained 76 unique phosphosites. We obtained four representative unique phosphomotifs (three for serines and one for threonines). As expected, the most represented motif found was S-P (Fig. 2A), present in 42.11% of the dataset, which corresponds to the minimum consensus site of Pro-directed kinases, such as ERK1, p38MAPKs, Cdk1, Cdk2, Cdk4, and Cdk5 [60]. The second phosphorylation consensus sequence found was S-x-x-x-x-P, which contains the S-x-x-x-S/T consensus site of the yeast homologue of GSK-3 kinase, Mck1. This motif was present in 11.84% of the dataset. Interestingly, we found the motif S-x-x-E, one of the consensus sites described for polo kinase-dependent phosphorylation and for casein kinase II (S/T-x-x-D/E), present in 11.84% of the dataset. Within this consensus site, we can infer the D/E/N-x-S motif described for the budding yeast polo-like kinase Cdc5 [61] and the two motifs described for casein kinase I: pS/pT-x_1-2_-S/T and D/E-x_1-2_-S/T. Finally, we observed the T-P motif in 7.89% of the phosphosites. These results suggest that PP2A^Cdc55^ apart of counteracting Cdk1 and Cdc5 as described, could be counteracting other Pro-directed kinases, GSK3 kinase, casein kinase I, and casein kinase II.

Motif sequences, their scores, and fold increase are shown in Fig. 2B. Interestingly, T-P motif presented the highest fold increase, closely followed by S-P motifs. Our results suggest a greater regulation of T-P sites over S-P sites by PP2A^Cdc55^ in mitotic cells, as recently reported [62, 63]. The motifs uncovered also suggest that PP2A^Cdc55^ could counteract other kinases apart from Cdk1 and Cdc5 Polo-like kinase. Four kinases are also found hyperphosphorylated: Snf1, Cla4, Bcy1, and Cdc37, suggesting that PP2A^Cdc55^ could directly regulate their kinase activity.

On the other hand, Cdk1-dependent phosphoproteome was uncovered in a similar study, where approximately 314 proteins containing Cdk1 consensus sites were identified as likely Cdk1 targets in budding yeast [49]. Since Cdk1 is the main kinase counteracted by PP2A^Cdc55^ phosphatase, we compared our list of 55 potential PP2A^Cdc55^ substrates to the Cdk1-dependent dataset and found 20 proteins that were common in both studies (Fig. 2C). The common proteins are summarized in Fig. 2C, right panel, and they are likely to be regulated by both Cdk1 and PP2A^Cdc55.^

### Novel roles for PP2A^Cdc55^ phosphatase in cytokinesis and endocytosis

To uncover new processes related to PP2A^Cdc55^, Gene Ontology (GO) analysis of the 55 statistically significant proteins were done (Table 1). GOs related to cytoskeleton organization and actin filament organization were identified. Next, to expand the analysis, we considered the 247 proteins found in at least two enrichment approaches (Fig. 1G). Functional clustering of the proteins that displayed enhanced phosphorylation in our dataset is presented in Additional file 8 and summarized in Table 2. We found a strong enrichment for cell cycle-related functional categories such as cell cycle, mitotic cell cycle, budding, cell polarity, actin cytoskeleton, and cytokinesis. Most of these processes are related to mitosis events, consistent with a PP2A^Cdc55^ role in mitosis and our analysis of mitotic arrested cells.

**Table 1:** GO categories of the 55 statistically significant phosphoproteins

Term	Count	%	*P* value	Genes	List total	Pop hits	Pop total	Fold enrichment	Benjamini
GO:0007010 cytoskeleton organization	13	25	7,37 E-07	ABP1, VRP1, BBC1, SPA2, CDC3, ENT1, YRB1, CDC37, KEL1, GCS1, YSC84, KIP2, CLA4	48	252	5557	5,97	7,24 E-04
GO:0030029 actin filament-based process	9	18	9,97 E-06	BBC1, SPA2, ENT1, ABP1, VRP1, KEL1, GCS1, AIM21, YSC84	48	129	5557	8,08	0,0048834
GO:0030036 actin cytoskeleton organization	8	16	5,35E-05	BBC1, SPA2, ENT1, ABP1, VRP1, KEL1, GCS1, YSC84	48	119	5557	7,78	00173649
GO:1902589 single-organism organelle organization	14	27	3,00 E-04	ABP1, VRP1, BBC1, SPA2, ENT1, SHP1, CDC3, CDC37, YRB1, RSC2, KEL1, GCS1, KIP2, YSC84	48	528	5557	3,07	0,071123
GO:0022603 regulation of anatomical structure morphogenesis	5	10	3,29 E-04	SPA2, PAL1, VRP1, RSC2, KEL1	48	40	5557	14,47	00624948
GO:0007015 actin filament organization	6	12	3,63 E-04	ENT1, ABP1, VRP1, KEL1, GCS1, YSC84	48	74	5557	9,39	00576188
GO:0051493 regulation of cytoskeleton organization	6	12	4,36 E-04	SPA2, ABP1, YRB1, VRP1, KEL1, KIP2	48	77	5557	9,02	00593868

**Table 2:** Major GO categories of the 247 common phosphoproteins from Fig. 1G

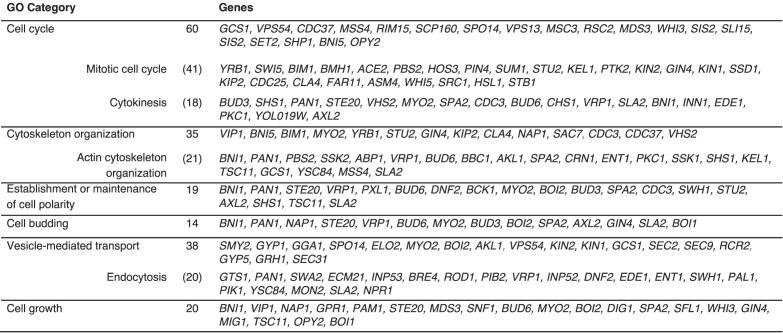

PP2A^Cdc55^ has been recently shown to monitor membrane trafficking and bud growth, integrating several cues to the mitotic entry regulators Swe1 and Mih1 [34] (Wee1 and Cdc25 in mammals). Interestingly, we found components of the cell wall integrity pathway, the Pkc1 and Bck1 kinases, and Zeo1. Moreover, we have been able to identify a physical interaction between Zeo1 and Cdc55 (see below), suggesting that Zeo1 is likely to be a PP2A^Cdc55^ substrate. We also identified other proteins related to budding such as Bud3, Bud6, Gin4, and Nap1.

Interestingly, many proteins required for cytokinesis like Inn1, Boi1, Shs1, and Cdc3 were also found among the PP2A^Cdc55^-dependent phosphoproteome, as well as proteins involved in the general organization of the actin cytoskeleton like Bud6, Bni1, and Spa2. On the other hand, we also found proteins related to vesicle-mediated transport. Control of membrane structures, cell membrane trafficking, and endocytosis has recently been linked to cytokinesis processes [64], and mammalian homolog PP2A-B55 has been related to the reformation of the nuclear envelope and the Golgi apparatus during telophase [40]. Finally, we also identified proteins related to osmotic stress and nutrient response. Thus, PP2A^Cdc55^ phosphatase seems to play a key role in sensing several cues of the environmental conditions, cell growth, and cell structure and integrating them into cell cycle regulation. In our screen, we also found proteins related to signal transduction and organelle organization, processes that are monitored and/or coordinated within the cell cycle (see Additional file 8).

**Figure 3:**
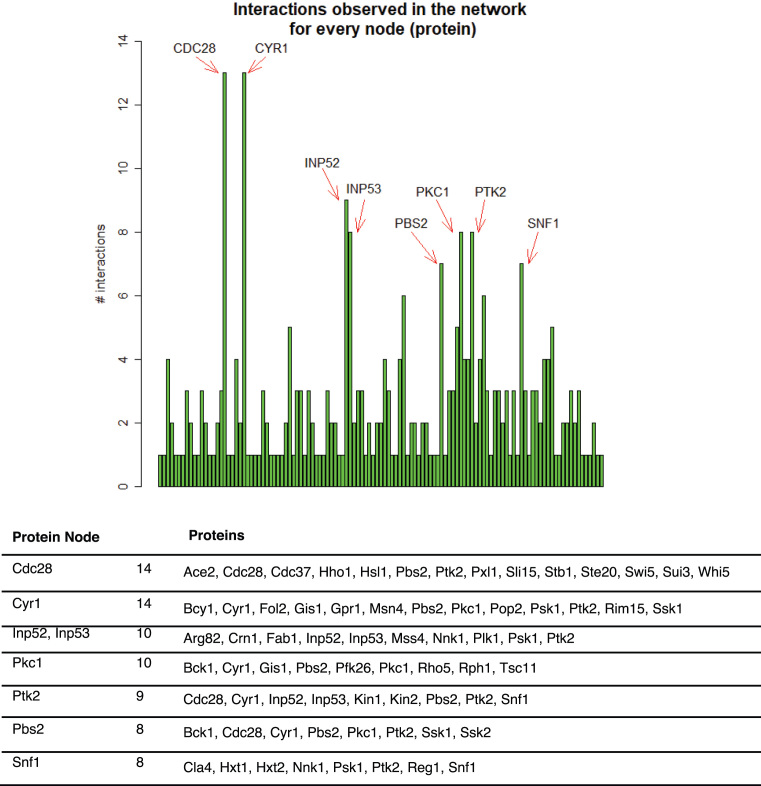
The Interaction Network analysis identified eight protein nodes related to PP2A-Cdc55. Distribution of the number of interactions identified eight protein nodes with more than seven interactions. The proteins present in the eight protein interactions nodes are shown.

A String Network Analysis of the 247 hyperphosphorylated proteins is shown in Additional file 9 and the list of interactions in Additional file 10. To note, the Cdc28 was found in only one of the enrichment approaches, but we included it in the String analysis since it was previously reported that PP2A^Cdc55^ counteracts Cdc28 activity [2, 8, 63]. We plotted the number of interactions found for each protein, and we identified eight proteins with more than seven interactions: Cdc28, Cyr1, Inp52, Inp53, Pkc1, Ptk2, Pbs2, and Snf1 (Fig. 3). Cdc28 and Pkc1 [34, 35] were previously linked with PP2A^Cdc55^. Pkc1 is a serine/threonine kinase involved in cell wall organization, actin filament organization, and bud selection that has recently been related to PP2A^Cdc55^, as it controls the binding of Igo1/2 proteins to PP2A [36]. On the other hand, Inp52 and Inp53 are two members of a conserved family of polyphosphatidylinositol phosphatases (the yeast synaptojanins) involved in endocytosis, cell growth, actin cytoskeleton organization, and bud site selection (reviewed in [65]). Those processes were identified in our GO analysis, suggesting that the role of PP2A^Cdc55^ in actin cytoskeleton and cell wall organization might be performed via Pkc1 and/or Inp52/3.

Cyr1 is the adenylate cyclase that synthesizes cAMP from ATP and is required for cAMP-dependent protein kinase signaling (PKA pathway). Cyr1 is involved in nutrient signaling, cell cycle progression, stress response, sporulation, and longevity [66, 67]. In addition, Snf1 is an AMP-activated serine/threonine kinase (the homologue of mammalian AMPK) involved in the regulation of transcription of glucose-repressed genes, sporulation, filamentous growth, G1-S transition, general stress response, and longevity [68, 69]. Pbs2 is a MAPK kinase of the Hog1 signaling pathway, which controls gene expression and cell cycle progression during G1 and G2 upon osmotic stress [70, 71]. Therefore, Cyr1, Snf1, and Pbs2 have essential roles in cell growth, cell cycle, and stress response and are potential regulation nodes of PP2A^Cdc55^, highlighting the importance of the PP2A^Cdc55^ in those processes.

### Validation of novel PP2A^Cdc55^ substrates in mitosis

*cdc55Δ* cells exhibit elevated tyrosine 19 phosphorylation on Cdk1 due to dysregulation of Swe1 and/or Mih1 [27, 29, 31, 72]. We first confirmed that we could detect this hyperphosphorylation of Cdk1-Y19 in *cdc55Δ* cells in our phosphoproteome screen (VGEGTyGVVYK, Y6 phosphoRS site probability >89%).

**Figure 4:**
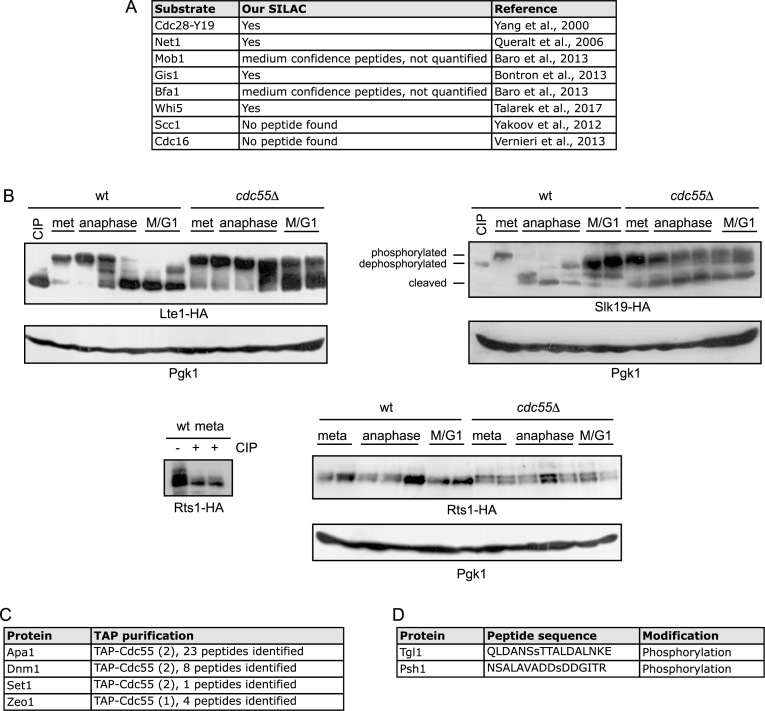
In vivo validation of PP2A^Cdc55^ novel substrates. A) Summary of already known PP2A^Cdc55^ substrates identified in our SILAC experiments. B) Validation of PP2A^Cdc55^ substrates. Strains Y1223 (*MATa LTE1–PK_3_::LEU2 MET-CDC20::LEU2*), Y1224 (as Y1223, but *cdc55Δ*), Y1240 (*MATa RTS1–PK_6_::TRP1 MET-CDC20::LEU2*), Y1241 (as Y1240, but *cdc55Δ*), Y1277 (*MATa SLK19-HA_6_::HIS3 MET-CDC20::LEU2*), and Y1278 (as Y1277, but *cdc55Δ*) were arrested in metaphase by Cdc20 depletion and synchronously released in anaphase by Cdc20 re-introduction. Lte1, Rts1, and Slk19 phosphorylation status were identified by western blot. Native protein extracts from metaphase samples were treated with alkaline phosphastase (CIP lane) as dephosphorylation controls. C) Proteins identified as PP2A^Cdc55^ physical-interactor proteins after TAP purification experiments. Protein extract from Y614 strain containing a TAP-Cdc55 (*MATa*, *CDC14-HA_6_::HIS3 TAP::CDC55 GAL1-CDC20::URA3*) was prepared, and TAP purification assay was performed as described in Methods. D) Proteins identified phosphorylated and co-eluted with HA-Cdc55. Protein extract from Y2541 strain containing an HA-Cdc55 (*MATa HA_3_::CDC55 GAL1-CDC20::LEU2*) was prepared, HA-Cdc55 was purified, and phosphopeptide enrichment was performed as described in Methods.

We next searched for already known PP2A^Cdc55^ substrates (Fig. 4A), as we previously published an extended study about Net1 being a PP2A^Cdc55^ substrate and its functional relevance for mitotic exit regulation [8]. Net1 was identified as being hyperphosphorylated in the *cdc55*∆ mutant, suggesting our approach to broadly identify substrates worked. In addition, Mob1 protein was identified in this phosphoproteomic study as a low confidence phosphopeptide, which we recently validated as a new PP2A^Cdc55^ substrate and demonstrated functional relevance for MEN activation [43]. Based on that result, we looked for other MEN components in our PP2A^Cdc55^-dependent phosphoproteome and found Lte1 hyperphosphorylated in the *cdc55∆* mutant. We further explored Lte1 phosphorylation at the metaphase-to-anaphase transition (Fig. 4B). Wild-type and *cdc55∆* cells were arrested in metaphase by Cdc20 depletion and released into synchronous anaphase by Cdc20 re-introduction. In wild-type cells, Lte1 was dephosphorylated in anaphase and transition to G1 (M/G1). In contrast, Lte1 was hyperphosphorylated in *cdc55∆* cells at the indicated times, suggesting it is likely to be a PP2A^Cdc55^ substrate. Native protein extracts from metaphase samples were treated with alkaline phosphatase as a control of phosphorylation. Additional MEN components, Cdc14 and Kin4, were also identified in our phosphoproteome analyses as putative new substrates of PP2A^Cdc55^ (Additional file 3), suggesting a closer regulation of the whole MEN pathway by PP2A^Cdc55^ phosphatase.

On the other hand, one component of the FEAR pathway was also identified in our PP2A^Cdc55^-dependent phosphoproteome, Slk19, which is a kinetochore-associated protein involved in chromosome segregation and Cdc14 release. We explored Slk19 protein modifications in the metaphase- to-anaphase transition as we had done for Lte1. In wild-type cells, Slk19 is phosphorylated in metaphase and, upon anaphase entry, undergoes cleavage. In contrast, Slk19 was hyperphosphorylated in *cdc55∆* cells throughout anaphase, and although it underwent cleavage, Slk19 showed an altered migration pattern of the cleaved form. This result suggests that PP2A^Cdc55^ is required to dephosphorylate Slk19.

In addition, Rts1, the second regulatory subunit of PP2A^Cdc55^, was also identified in our phosphoproteome analysis. PP2A^Rts1^ is located at the centromeres during mitosis and prevents cohesin cleavage by separase [73], and it is also required for cell size control [74]. Rts1 was dephosphorylated in M/G1 in wild-type cells (Fig. 4B). In contrast, Rts1 was hyperphosphorylated in *cdc55∆* cells at the indicated times. Native protein extracts from metaphase samples were treated with alkaline phosphatase as control. These results indicate that Rts1 is hyperphosphorylated in the absence of PP2A^Cdc55^, suggesting that PP2A^Cdc55^ is required to dephosphorylate Rts1.

### Zeo1 and other potential PP2A^Cdc55^ substrates interact with the PP2A^Cdc55^ phosphatase *in vivo*

Finally, we used Cdc55 pull-down strategies to further validate new potential substrates of PP2A^Cdc55^ and further explore specific binding partners of this phosphatase. We first used tandem affinity purification (TAP) to find new Cdc55 interactors, using a strain expressing a TAP-epitope tagged Cdc55 (TAP-Cdc55). TAP involves fusion of the TAP epitope (protein A from *Staphylococcus aureus* and the calmodulin binding peptide arranged in tandem and separated by a TEV protease cleavage site) to the target protein of interest. The fusion protein and their associated components were then recovered by two rounds of affinity purifications. Eluted fractions were then directly processed by high sensitive LC-MS/MS methods. A strain without the TAP epitope was used as control. The peptides identified in the TAP-Cdc55 pull-down that are not found in the negative control purification are considered novel Cdc55-associated proteins (Additional file 11). Among them, four proteins—Zeo1, Apa1, Dnm1, and Set1—were also found hyperphosphorylated in our PP2A^Cdc55^-dependent phosphoproteome (Fig. 4C), suggesting they are likely to be PP2A^Cdc55^ substrates.

We next performed a second Cdc55 purification using HA-Cdc55 tagged strain and HA-affinity columns. The eluted fractions were subjected to TiO_2_ enrichment to search for proteins that are undergoing phosphorylation modifications among the newly identified Cdc55 associated proteins. The enriched peptides were subjected to LC-MS/MS. Peptides identified are shown in Additional file 12. Among them, Psh1, Tgl1, Hos3, and Sro9 were identified as Cdc55-interacting proteins. Peptide and protein modifications were obtained using the Mascot search engine. Interestingly, Tgl1 and Psh1 were also found in our quantitative phosphoproteomic study of potential PP2A^Cdc55^ substrates (Fig. 4D). Considering that those proteins interact physically with PP2A^Cdc55^ and are found hyperphosphorylated in *cdc55Δ* cells, they are likely new PP2A^Cdc55^ substrates.

We observed little overlap between our SILAC studies with the pull-down experiments. This is consistent with the long-held notion that kinase-substrate interactions are commonly weak and transient, thus difficult to detect by purification-based protein interaction screens.

### Docking models of PP2A^Cdc55^ and Mob1 highlight potential binding interfaces for Cdc55 and Mob1

To explore the interaction surface of Cdc55 and its Cdk1-dependent substrates, we performed rigid-body computational docking using HADDOCK [75] (version 2.2, ). Except for the previously validated substrate Mob1 [43], none of the other substrates have structural data for regions with Cdc55-dependent phosphosites. As such, we built a homology model of Cdc55 based on the crystal structure of the mammalian homologue B55 and used the published crystal structure of Mob1 to build 100,000 models of the Cdc55/Mob1 complex, using knowledge of a Tau binding region on B55 to restrict the search space of the docking calculations on the Cdc55 surface.

**Figure 5:**
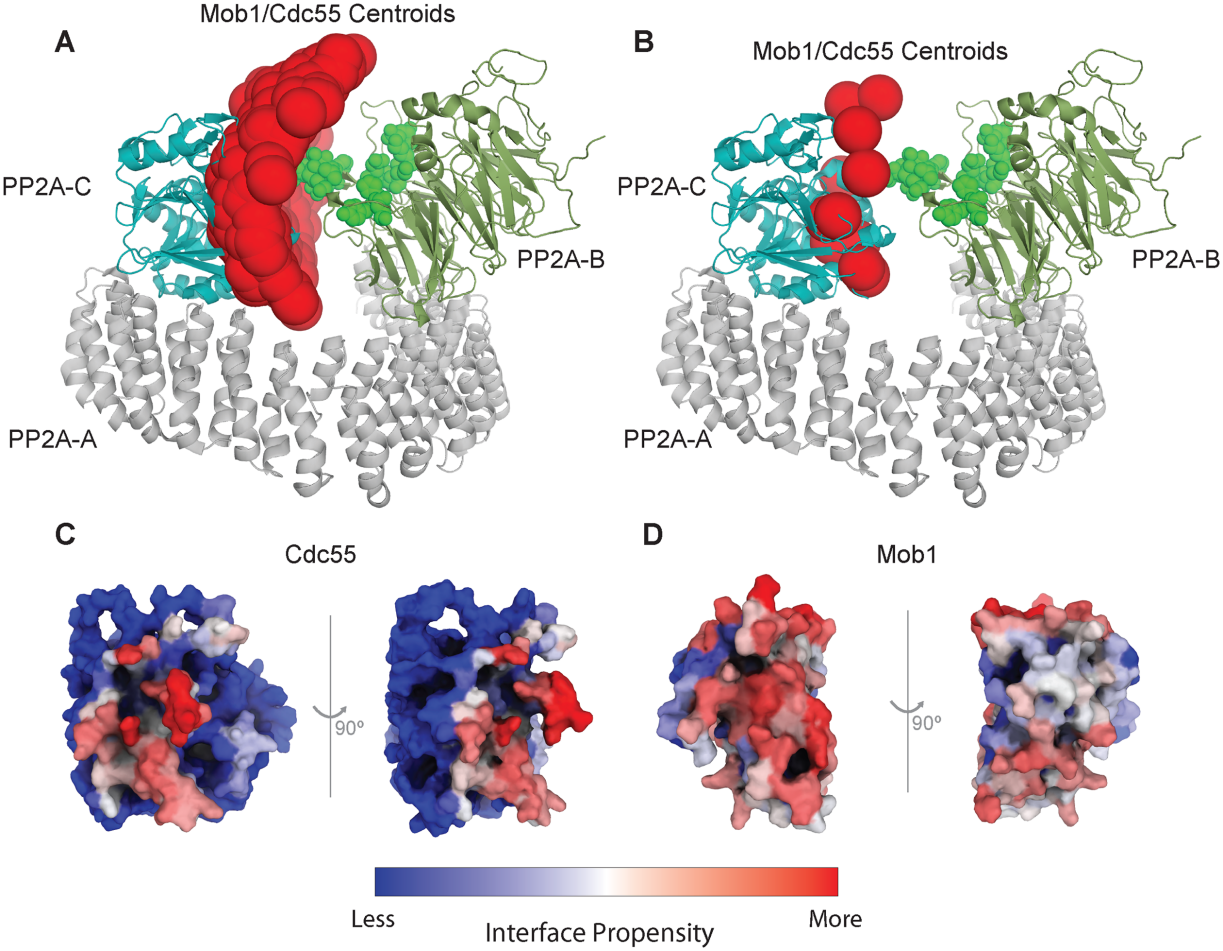
Docking models of PP2A^Cdc55^ and Mob1 highlight potential binding interfaces for Cdc55 and Mob1. A) Representatives of the best 10,000 models of the Cdc55/Mob1 complexes superimposed on the human heterotrimeric PP2A structure (PDB 3dw8). Red spheres represent the centers of mass of representative models. The regulatory B55 subunit, homologous to Cdc55, is shown in green, while the catalytic subunit is shown in blue. Residues previously identified as interacting with Tau are represented as green spheres. B) Representatives of the filtered subset of 294 models of Cdc55/Mob1 after filtering for catalytic subunit distance. C–D) Per-residue interface propensities (log2 scaled, red showing higher values) calculated on 294 filtered models of Cdc55/Mob1, respectively.

The best 10,000 models, ranked by intermolecular energy, cluster into 437 representative binding poses that show a smooth distribution of Mob1 across the surface of the β-propeller of PP2A (Fig. 5A). Filtering these models for those where Mob1 adopts a binding pose compatible with dephosphorylation by the catalytic subunit of PP2A, measured by the distance between a known phosphosite (S80) and the proton donor on PP2A (H118), narrows down the possible interaction nodes to 294 models (12 clusters) with a very similar interaction surface (Fig. 5B). In these models, the predominant interface on Cdc55 is mediated by a protruding loop consisting of residues 84–90, which were shown to be critical for Tau binding and more recently to the binding of mitotic substrate PRC1; therefore, the Cdc55 residues interacting with its substrates seem to be conserved. This is shown more clearly by a statistical analysis of per-residue interface propensities where the residues 84–90 (marked in red) appeared concentrated in the more frequent interfaces (Fig. 5C). On Mob1, there is no such conserved narrow interface (represented as red residues broadly spread throughout the interphases), even among the binding poses consistent with the dephosphorylation function, although one face of the protein seems to be more favorable for interaction (Fig. 5D). Interestingly, most of these models are located in between the regulatory B55 subunit and the catalytic subunit of PP2A, which would be compatible with an open-close conformational change of the scaffold subunit. Indeed, a substantial degree of flexibility of the scaffold subunit has been observed upon formation of the core enzyme alone [76].

## Discussion

Mitotic exit depends on phosphatase activation in all organisms studied so far. PP2A^Cdc55^ is a major Cdk1-counteracting phosphatase during cell cycle progression and a principal mitotic regulator. To uncover new PP2A^Cdc55^ targets and functions during mitosis, we depleted *CDC55* in budding yeast and screened for hyperphosphorylated peptides enriched in metaphase-arrested cells in a quantitative SILAC-based approach. Non- phospho-enriched control samples indicated that most of the phosphorylation changes found can be attributed to PP2A^Cdc55^ inactivation and not to changes in protein abundance in the *cdc55Δ* mutant. None of the different phosphopeptide enrichment methods available provide a whole phosphoproteome. Each method provides varying degrees of selectivity and specificity of phosphopeptide enrichment, resulting in the identification of phosphopeptides of different nature. In fact, the ratio of monophosphorylated and multiphosphorylated peptides detected in each method varies considerably (42% and 58% in TiO2, 83% and 17% in SIMAC, and 90% and 10% in TiSH, respectively). Due to the intrinsic and distinct nature of the phosphoenrichment methods the overlapping among the three strategies is modest (Fig. 1), but the combination of the methods greatly enhances the number of phosphopeptides isolated of different nature.

We identified several kinases—Snf1, Cla4, Bcy1, and Cdc37—hyperphosphorylated in the absence of PP2A^Cdc55^, suggesting that processes regulated by these kinases are potentially regulated by PP2A^Cdc55^ phosphatase as well. Indeed, we found a major set of peptides containing Cdk1 consensus sites. In addition to Cdk1 consensus sites, we found other kinase consensus motifs enriched in the Cdc55-dependent phosphoproteome. It has been shown that Cdc5 kinase phosphorylation of Scc1 [44] and Bfa1 is counteracted by PP2A^Cdc55^ phosphatase [43]. In accordance, we identified a group of proteins containing the proposed Cdc5 polo-like kinase consensus sites (D/E/N-x-S/T) (Additional file 13).

While preparing this manuscript, two SILAC-based studies targeting PP2A^Cdc55^ [63] and the mammalian PP2A-B55 [62] were published. By comparing the phosphorylation status of Cdk1 substrates in the absence of PP2A^Cdc55^ at different cell cycle phases (G1, S, and G2) [63], they deciphered how PP2A^Cdc55^ contributes to determining the progressive phosphorylation of Cdk1 substrates. In contrast, our study focused on metaphase-arrested cells (when the PP2A^Cdc55^ activity is higher during mitosis), and we considered not only the Cdk1-counteracted substrates but all Cdc55-dependent phosphorylation sites for downstream analysis. Nevertheless, when we compared both datasets, we found 34% of the proteins in our data identified in [63] (19 common/55 proteins in our dataset, Additional file 14), indicating a high degree of overlapping in both studies. As expected, most of the overlapping proteins (16/55) were found when comparing the closest cell cycle stages, G2 in [63] and M in our dataset. Interestingly, although phosphorylated serines were more abundant, threonines showed the most dramatic fold-increase in our X-motif analysis, in agreement with the two published studies showing that this phosphatase has a threonine preference [62, 63].

Although the increased phosphorylation of the proteins identified in the *cdc55Δ* mutant is either a direct or indirect effect of PP2A^Cdc55^ inactivation, new regulated PP2A^Cdc55^ processes can be discovered. In fact, GO analysis of the 247 common proteins in at least two approaches identified several processes related to mitosis, actin cytoskeleton organization, budding, and cytokinesis. Budding impinges a dramatic re-arrangement of the cell structure and morphogenesis changes, GO categories that were also found in our study. In addition, we identified proteins related to osmotic stress and nutrient response. Thus, PP2A^Cdc55^ phosphatase seems to play a key role in sensing several cues of the environmental conditions, cell growth, cell polarity, and cell structure and integrating them to regulate the cell cycle.

PP2A^Cdc55^ has been shown to integrate membrane growth into mitosis regulation via Rho1 and Pkc1 [34–36], regulators of the cell wall integrity pathway. Indeed, Pkc1 was found hyperhosphorylated in the absence of Cdc55 and was identified as a protein node in our string analysis. Regulation of Cdc55 activity by Pkc1 phosphorylation in the context of blocking membrane trafficking has also been uncovered [35]. Thus, mutual regulation of Pkc1 and Cdc55 seems to occur and they might share several substrates. Interestingly, we found Zeo1, an upstream negative regulator of the cell integrity pathway, to be hyperphosphorylated in the absence of Cdc55 phosphatase. In addition, we showed that Cdc55 and Zeo1 potentially interact through co-purification assays; thus, Zeo1 is likely a new PP2A^Cdc55^ substrate. Altogether, we conclude that PP2A^Cdc55^ could counteract other kinases apart from Cdk1 and Cdc5, like Pkc1, as well as regulate their activity.

In previous studies, we identified a dual regulation of the MEN by PP2A^Cdc55^ phosphatase, which dephosphorylates Bfa1 and Mob1. Here, we found that other MEN components were hyperphosphorylated in the Cdc55-dependent phosphoproteome, and we validated Lte1 as a likely substrate of PP2A^Cdc55^ phosphatase. Thus, PP2A^Cdc55^ seems to closely regulate the MEN pathway by dephosphorylating other elements apart of Bfa1 and Mob1. In addition, we validated Slk19, a component of the Cdc14 early anaphase release (FEAR) pathway, as likely PP2A^Cdc55^ substrate during mitotic exit as well as other potential substrates (Apa1, Dnm1, Set1, Psh1, and Tgl1) by co-purification with Cdc55.

To better understand how PP2A^Cdc55^ interacts with its substrates, we built computational docking models of Cdc55 with its recently described Cdk1-dependent substrate, Mob1. Interestingly, residues 84–90, located at the Cdc55 groove structure, were predicted to interact with Mob1. This same interface has been shown to be critical for Tau and PRC1 binding to mammalian B55 *in vivo*. Further studies, including substrates regulated by other PP2A^Cdc55^-counteracted kinases, could help elucidate how this specific phosphatase recognizes and interacts with its substrates.

This work attempts to bring new insight into the mitotic exit regulation picture, with a special focus on PP2A^Cdc55^ functions in this critical phase of cell division. A profound understanding of mitotic exit regulation could set the stage for new therapeutic strategies, since failure to progress normally through mitotic exit can induce cell death and could be exploited to kill hyper-proliferating cancer cells. The study of phosphatase holoenzymes, and especially the regulatory phosphatase subunits such as Cdc55, provides valuable information for the development of new pharmacological inhibitors or modulators that selectively target specific phosphatase complexes.

### Potential implications

Dysregulation of PP2A phosphatases has been found in many solid cancers and leukemias. PP2A-B55 and its highly conserved homolog in budding yeast, PP2A^Cdc55^, regulate the cell cycle and are required for efficient mitotic exit. Budding yeast is thus a powerful model to gain insight into mitotic exit regulation, specifically, the activities of PP2A phosphatase holoenzymes. This could promote the design of new therapeutic strategies, since failure to progress normally through mitotic exit may be exploited to kill hyper-proliferating cancer cells. Here, we used phosphoproteomics of Cdc55-deficient cells to uncover new PP2A^Cdc55^ substrates and functions in mitosis. We also reveal new kinases potentially counteracted and regulated by PP2A^Cdc55^ phosphatase. In particular, Pkc1 was discovered as a significant PP2A^Cdc55^ regulation node. Finally, we attempted to gain insight into Cdc55-susbtrate interaction using docking models of Cdc55 and Mob1 substrate, which suggest a specific interface for substrate interaction.

## Methods

### Yeast strains, plasmids, and cell cycle synchronization procedures

All yeast strains used in this study were derivatives of W303. Epitope tagging of endogenous genes was performed by gene targeting using polymerase chain reaction products. Endogenous *CDC55* was N-terminal tagged as previously described [8]. Metaphase arrest by Cdc20 depletion was also performed as previously described [77].

### Stable isotope labeling of yeast cells and preparation of yeast protein extracts for phosphoproteomic analysis

For each biological replicate, yeast cells were labeled with stable isotopes and protein extracts prepared as previously described [78]. In brief, cells were grown in minimum media containing either 100 mg/L arginine and 100 mg/L lysine or 100 mg/L ^13^C_6_-arginine and 100 mg/L ^13^C_6_-lysine (Cambridge Isotope Laboratories Inc., US). Y859 (*MATa lys2Δ::TRP1*, *arg4Δ::HIS3 MET-CDC20::LEU2*) and Y858 (as Y859 but *cdc55Δ*) cells were grown in free-methionine minimum media containing ^13^C_6_-lysine and -arginine (heavy) or unmodified arginine and lysine (light), respectively. Both strains were synchronized at the metaphase-to-anaphase transition by adding methionine to the media. Protein extracts were prepared by mechanical lysis using glass beads in presence of protein inhibitors (Complete EDTA-free, Roche, Spain) and 2xphosphatase inhibitors PhosStop (Roche, Spain). Cell lysates were mixed 1:1 and digestion with trypsin was performed. Approximately 400 μg of the mixed heavy/light protein sample was processed for in-solution digestion as previously described [79]. Proteins were reduced with 5 mM DL-Dithiothreitol (DTT) for 30 min at 37°C and alkylated with 10 mM iodoacetamide for 30 min at 30°C. Samples were diluted five times with 25 mM ammonium bicarbonate, trypsin (Promega, Spain, ratio enzyme: protein 1:10) was added, and samples were incubated overnight at 37°C. Digestion was stopped by addition of formic acid.

### Phosphopeptide enrichment

Three strategies were used for phosphopeptide enrichment. In the first approach, phosphopeptide enrichment by SIMAC was done as previously described [78]. Peptide samples were added to an immobilized metal affinity chromatography suspension (Phos-Select, Sigma, Spain) and were incubated for 1 hat room temperature. The flow-through was collected, and the immobilized metal affinity chromatography resin was washed once with 50 μl 50% acetonitrile (ACN) and 0.1% TFA. The wash fraction was pooled with the flow-through. Acid elution was then carried out by adding 50 μl 30% ACN and 1% TFA and incubating for 5 min at room temperature. After this step, alkaline elution was done with 50 μl 0.5% NH_4_OH pH 10.5, followed by 30-min incubation at room temperature. For further enrichment of phosphopeptides, the flow-through fraction and the acid eluate were incubated with TiO_2_ beads (GL Sciences, Tokyo, Japan) and incubated with shaking for 1 h at 30°C. The TiO_2_ beads were washed twice with 80% ACN and 1% TFA and once with water. Bound peptides were eluted from the beads with 0.5% NH_4_OH pH 10.5 for 30 min at 30°C. Eluted peptides were dried via centrifugal evaporation, resuspended with 1 μl formic acid and 15 μl water and analyzed using nano-LC-MS/MS on an LTQ-Orbitrap (Themo Scientific) mass spectrometer.

In the second strategy, phosphopeptide enrichment was done using TiO_2_ chromatography following the product specifications (TiO_2_ Mag Sepharose, GE Healthcare, Fisher Scientific, Spain). An aliquot of 100 μg was separated to be further processed and analyzed without phosphopeptide enrichment. All samples (enriched and non-enriched for phosphopeptide) were dried via centrifugal evaporation and subjected to fractionation with a high pH reversed phase peptide fractionation kit (Pierce, Fisher Scientific, Spain). The peptides were eluted in nine fractions of increasing ACN concentration of 5% ACN to 75% ACN. The nine eluted fractions were dried via centrifugal evaporation, resuspended in 1% formic acid (FA), and analyzed in a nanoAcquity liquid chromatographer (Waters) coupled to an LTQ-Orbitrap Velos (Thermo Scientific, Spain) mass spectrometer.

In the third approach, a combination of enrichment and fractionation methods was used (The “TiSH” method: TiO_2_-SIMAC-HILIC) as previously described [80]. Briefly, peptide digest was first pre-enriched in phosphopeptides using TiO_2_ chromatography [81] (5 μm, GL Sciences Inc, Japan) followed by SIMAC purification [82]. The mono-phosphorylated peptide fraction from the SIMAC enrichment was further subjected to a second TiO_2_ purification. The mono-phosphorylated fraction was then pre-fractionated by HILIC chromatography (Hydrophilic Interaction Liquid Chromatography, Column TSK Gel Amide 80, 15 cm, 0.3 mm ID) using a 40-min gradient from 90% B buffer (95% acetonitrile, 0.1% TFA) to 60% B buffer. Twenty-five fractions were collected, which were pooled into a final five fractions that were then analyzed by reverse-phase LC-MS/MS. The multi-phosphorylated fraction from SIMAC was directly analyzed by LC-MS/MS after desalting and concentration using a Poros Oligo R3 (ABSciex, US) Reversed phase (RP) micro-column.

### LC-MS/MS analysis

For the first approach, the peptides were analyzed using nano-LC-MS/MS on an LTQ-Orbitrap Velos (Thermo Scientific, Spain) mass spectrometer. Peptides were separated on a BioBasic C-18 PicoFrit column (75μm Øi, 10 cm, New Objective, Woburn, MA) at a flow rate of 200 nL/min. Water and ACN, both containing 0.1% formic acid, were used as solvents A and B, respectively. Peptides were trapped and desalted in the trap column for 5 min. The gradient was started and kept at 10% B for 5 min, ramped to 60% B over 60 min or 120 min depending on the sample complexity, and kept at 90% B for another 5 min. Peptides (m/z 400–1,400) were analyzed on the LTQ-Orbitrap velos in full Scan MS mode with a resolution of 60,000 FWHM at 400m/z; up to the seven most abundant peptides were selected from each MS scan and then fragmented using collision-induced dissociation in a linear ion trap using helium as collision gas at 7,500 FWHM and 30-sec exclusion time. Generated .raw data files were collected with Thermo Xcalibur v.2.2.

For the second approach, the peptides (enriched and non-enriched) were resuspended in 1% FA and were injected for chromatographic separation. Peptides were trapped on a Symmetry C18^TM^ trap column (Waters, US) and were separated using a C18 reverse-phase capillary column (75 μm Øi, 25 cm, nano Acquity, 1.7 μm BEH column; Waters, US). The gradient used for the elution of the peptides was 1% to 35% B in 90 min, followed by a gradient from 35% to 85% in 10 min (A: 0.1% FA; B: 100% ACN, 0.1%FA), with a 250-nL/min flow rate. Eluted peptides were subjected to electrospray ionization in an emitter needle (PicoTip, New Objective, US) with an applied voltage of 2,000 V. Peptide masses (*m/z* 300–1,700) were analyzed in data-dependent mode where a full Scan MS was acquired in the Orbitrap with a resolution of 60,000 FWHM at 400m/z. Up to the 10 of most abundant peptides (minimum intensity of 500 counts) were selected from each MS scan and then fragmented using collision-induced dissociation in the linear ion trap using helium as collision gas. Multistage activation was enabled to favor the detection of phosphopeptides. The scan time settings were: full MS: 250 ms and MSn: 120 ms. Generated .raw data files were collected with Thermo Xcalibur v.2.2.

For the third strategy, the peptides were resuspended in 0.1% TFA and analyzed using an Easy-nanoLC (Thermo Fisher Scientific, Proxeon, Denmark) coupled to an LTQ-Orbitrap Fusion Tribride mass spectrometer (Thermo Fisher Scientific, Denmark). Peptides were loaded onto a pre-column of 2 cm Reprosil –Pur C18 AQ 5 µm RP material (Dr. Maishc, Ammerbuch-Entrigen, Germany) using the EASY-LC system and eluted directly onto a 20-cm-long fused silica capillary column (75 µm ID) packed with Reprosil- Pur C18 AQ 3 µm RP material. The peptides were separated using a gradient from 0 to 34% B (A buffer: 0.1% FA; B buffer: 90% ACN/0.1% FA) at a flow rate of 250 nL/min over 30–60 min depending on the UV trace of the HILIC fractions. The peptides (m/z 400–1,400) were analyzed in full MS mode using a resolution of 120,000 FWHM at 200 m/z and the peptides were selected and fragmented using helium as collision gas; the fragment ions were recorded in the LTQ with low resolution (rapid scan rate). A maximum of 3 sec was allowed between each MS and for MSMS the ion filling time was set to 40 ms and an AGC target value of 2E4 ions. Raw data was viewed in Xcalibur v2.0.7.

### Data analysis for peptide identification and quantification

To perform the sample data analysis, we compiled the raw files from the technical replicates of each phosphoenrichment method, obtaining a unique list of peptides and proteins for each method. Peptide identification was performed using Proteome Discoverer v1.4.1.14 (Thermo Scientific, Spain) and search against Swiss Prot/Uniprot *Saccharomyces cerevisiae* database (v. January 2016) with SequestHT search engine. Both a target and a decoy database were searched to obtain a false discovery rate (FDR). To improve the sensitivity of the database search, Percolator (semi-supervised learning machine) was used to discriminate correct from incorrect peptide spectrum matches. The PhosphoRS node was used to provide a confidence measure for the localization of phosphorylation in the peptide sequences identified with this modification.

Database searches were performed with the following parameters: precursor mass tolerance 10 ppm, fragment mass tolerance 0.6 Da, cysteine carbamydomethylation as fixed modification, and 2 missed cleavage for trypsin. Variable modifications considered were phosphorylation on S/T/Y and K/R label:^13^C_6_ and oxidation (M).

Only peptides with high confidence Percolator q of 0.01 (FDR <1%) were considered for further analyses.

Peptide quantification from SILAC labels was performed with Proteome Discoverer v1.4. The log2-ratio value associated with each peptide was calculated as a weighted average of the scans used to quantify the peptide, as described elsewhere [58, 59], and the data were normalized based on the median. Only quantified peptides detected as statistically significant (high confidence FDR <0.01) were selected. The processing of the data was performed in R (v.3.3.1) with the help of the “xlsx,” “rvest,” “Vennerable,” and “Venneuler” packages. Briefly, the H/L ratios from the samples (TiSH, SIMAC, and TiO_2_) were averaged for every phosphopeptide. The resulting list was filtered to keep only the phosphopeptides of interest, that is, peptides with a coefficient of variation (CV) between samples <40%, peptides without CV (peptides only appearing in one sample), and peptides with a CV >40% that show a H/L ratio in all the samples <0.75. Statistical significance was assessed at 5% (two-tailed Student's *t*test; *P*<0.05).

### Phosphorylation motif analysis

The Phosphorylation Motifs Enrichment Analysis was performed with the motif-X web tool [83, 84]. Before the analysis, the phosphosites were aligned so that the phosphosite was centered. Only the statistically significant peptides identified in at least 2 of 3 SILAC (62 phosphopeptides from Fig. 1H) approaches were used to search for enriched motifs against the SGD yeast proteome database as a background.

### TAP purification

Protein extracts were prepared by mechanical lysis using glass beads in presence of protein inhibitors (Complete EDTA-free, Roche, Spain) and 2xphosphatase inhibitors PhosStop (Roche, Spain). TAP and CBP epitopes, fusion proteins, and associated proteins were recovered from cell extracts by affinity chromatography using an IgG-sepharose matrix. After washing, the Tobacco Etch Virus (AcTEV, Life technologies, Spain) protease was added to release the bound material. The eluate was incubated with calmodulin-coated beads in the presence of calcium. This second affinity step was required to remove not only the AcTEV protease but also traces of contaminants remaining after first affinity purification. After washing, the bound material was released with ethylene glycol tetra acetic acid. The calmodulin eluates from the TAP-purified complexes were precipitated with trichloroacetic acid (TCA) and directly subjected to LC-MS/MS. Pellets were dissolved with 20 μL of 50 mM ammonium bicarbonate (ABC). Cysteine residues were reduced by 2 mM DTT in 50 mM ABC at 60° for 20 min. Sulfhydryl groups were alkylated with 5 mM iodoacetamide (IAM) in 50 mM ABC in the dark at room temperature for 30 min. IAM excess was neutralized with 10 mM DTT in 50 mM ABC 30 min at room temperature. Then 5 μL of each sample was loaded onto a trap column (nanoLC column, 3 μ C18-CL, 75 μm x 15 cm; Eksigen, US) and desalted with 0.1% TFA at 2 μL/min during 10 min. The peptides were then loaded onto an analytical column (LC Column, 3 μ C18-CL, 75 μm x 15 cm; Eksigen, US) equilibrated in 5% acetonitrile 0.1% FA. Elution was carried out with a linear gradient of 5–35% B in A for 120 min (A: 0.1% FA; B: AN 0.1% FA) at a flow rate of 300 nL/min. Peptides were analyzed in a mass spectrometer nanoESI qQTOF (5600 TripleTOF, ABSciex, US). The tripleTOF was operated in information-dependent acquisition mode, in which a 0.25-s TOF MS scan 350–1,250 m/z, was performed, followed by 0.05-s product ion scans from 100to 1,500 m/z on the 50 most intense 2–5 charged ions. Protein identification was performed using ProteinPilot v4.0.8085 (ABSciex, US) or Mascot v2.3 (Matrix Science, UK) search engines. Protein Pilot default parameters were used to generate peak list directly from 5600 TripleTOF wiff files. The Paragon algorithm of ProteinPilot was used to search Expasy protein database (1072964 sequences). The proteomic analysis was carried out in the SCSIE_university of Valencia Proteomics Unit, a member of ISCIII ProteoRed Proteomics Platform. Peptides identified in two TAP-Cdc55 biological replicates and the untagged control had been deposited to the ProteomeXchange Consortium with the dataset identifier PXD007613.

### HA purification

Protein extracts were prepared by mechanical lysis using glass beads in presence of protein inhibitors (Complete EDTA-free, Roche, Spain) and 2xphosphatase inhibitors PhosStop (Roche, Spain). HA-Cdc55 fusion proteins and associated proteins were recovered from cell extracts by HA-agarose beads (Sigma, Spain). The eluates were precipitated with TCA and proteins were separated in a protein gel. After trypsin digestion, peptide was desalted by Strata X C18 column (Phenomenex, China) and vacuum-dried. A total of 1 μgdried peptide was reconstituted in a solution containing 65% ACN and 2% TFA and was saturated with glutamic acid (20 mg/ml, pH 2.0–2.5). Then the peptide solution was added to TiO_2_ (GL Science, Saitama, China) and was incubated for 20 min. The peptides were eluted once with 1.1% NH_4_OH solution in 50% ACN and once with 3% NH_4_OH solution in 50% ACN (diluted from 25% NH4OH solution). Two elute fractions were combined and vacuum-dried. Then, phosphopeptides were subjected to nanoelectrospray ionization followed by MS/MS on a Q-Exactive mass spectrometer (ThermoFisher Scientific, China). Peptide and protein modification were obtained using Mascot software. HA purification experiments were performed using BGI proteomic services and BGI bioinformatics department.

### Western-blot validation of cell cycle-dependent phosphorylated substrates

Cell synchronization by Cdc20 depletion and entry into synchronous anaphase by Cdc20 re-introduction were also performed as previously described [43]. Protein extracts for western blots were obtained by TCA protein extraction. Gels of 8–10% were used for electrophoresis. Antibodies used for protein staining were α-HA clone 12CA5 (Roche, Spain) and α-Pk clone SV5-Pk1 (Serotec, Bionova, Spain).

### Interaction maps and GO

The networks were created with the STRING database [85] by using the proteins quantified in at least two of three SILAC experiments (247 phosphoproteins from Fig. 1G) [86]. Only high-confidence interactions from experiments or databases were extracted and binary interactions were also discarded.

Classification into functional clusters and GO was performed with the DAVID bioinformatics tools using the 247 hyperphosphorylated proteins described above [87, 88]. Only clusters with an Enrichment Score >1.5 and GO terms with a *P* <0.001 were considered.

### Structure prediction of Cdc55

A structural model of full-length yeast Cdc55 (Uniprot AC: 2ABA_YEAST) was built by homology modeling. HHpred [89] identified the regulatory B55 subunit of the heterotrimeric human protein phosphatase PP2A (Uniprot AC: 2ABA_HUMAN; PDB: 3dw8_B) as a suitable template and provided a pairwise alignment. We then used the loop model protocol implemented in MODELLER 9v18 [90] to build 50 models of Cdc55, which were assessed and ranked with the DOPE statistical potential [91].

### Sampling the binding interface of the CDC55/Mob1 complex

Models of the interaction between Cdc55 and Mob1 were calculated using the data-driven docking software HADDOCK (version 2.2) [75]. As initial structures, we used the Cdc55 homology model with the lowest (best) DOPE score and the available crystal structure of Mob1 (PDB: 2HJN_A). We restricted the search on Cdc55 to solvent accessible residues within a 10-Å radius of the Tau binding region identified by NMR and mutagenesis experiments on the homologous B55 [3]. All residues are strictly conserved between the two proteins: E24, K45, F75, D76, Y77, L78, K79, S80, L81, E84, E85, K86, Y185, H186, and D204. For Mob1, we defined the entire surface of the protein as a possible interaction site. A residue was defined as solvent accessible if its main-chain or side-chain atoms had a relative solvent accessibility ≥15% as calculated by FREESASA [92] and the NACCESS scale.

We calculated 100,000 models using the data-driven rigid-body docking protocol in HADDOCK and kept the best 10,000 (top 10%) ranked by HADDOCK score for further analysis. We then superimposed these models on the heterotrimeric PP2A structure and calculated the distance between residues P81 in Mob1 (proxy for the phosphosite S80, not resolved in the crystal) and H118 (proton donor) in the catalytic subunit of PP2A. Using a threshold of 10Å as filtered, we obtained a list of 294 models, which we then grouped in 12 representative clusters using a fast contact-based interface similarity algorithm [93]. We also used these 294 models to calculate propensities for each individual residue to be part of the Cdc55/Mob1 interface. A residue was defined as part of the interface if any of its atoms was within 5Å of any atom of the partner protein.

## Availability of supporting data

The mass spectrometry proteomics data have been deposited to the ProteomeXchange Consortium [94] via the PRIDE [95] partner repository with the dataset identifier PXD007613.

The files uploaded correspond to: (1) Madrid Phospho Analysis 2016.msf. This file contains the proteins and peptides detected in the SIMAC-based enrichment assay (Method 1). It is generated (and can be open) by the “Proteome Discoverer” software with the following raw data: Elu1–12_75.raw, Elu1–12_75_bis.raw, Elu2–12_75.raw, FT-12_75.raw, FT-12_75_bis.raw. (2) reg1418_TiO2_13raw.msf. This file contains the proteins and peptides detected in the TiO2-based enrichment assay (Method 2). It is generated (and can be opened) by the “Proteome Discoverer” software with the following raw data: reg1418_TiO2_FTwash.raw, reg1418_TiO2_f1f8_160426190328.raw, reg1418_TiO2_f1f8_160503121600.raw, reg1418_TiO2_f2f9.raw, reg1418_TiO2_f2f9_160503145752.raw, reg1418_TiO2_f3.raw, reg1418_TiO2_f3f7FTwash.raw, reg1418_TiO2_f4.raw, reg1418_TiO2_f4f6.raw, reg1418_TiO2_f5.raw, reg1418_TiO2_f5_160503221654.raw, reg1418_TiO2_f6.raw, reg1418_TiO2_f7.raw. (3) MLarsen_Replique1.msf and MLarsen_replique 2.msf. These files contain the proteins and peptides detected in the TiSH-based enrichment assay (Method 3). It is generated (and can be opened) by the “Proteome Discoverer” software with the following raw data: FUS01268.raw, FUS01269.raw, FUS01270.raw, FUS01271.raw, FUS01272.raw, FUS01273.raw, FUS01274.raw, FUS01275.raw, FUS01276.raw, FUS01277.raw, FUS01278.raw, FUS01279.raw, FUS01280.raw, FUS01281.raw, FUS01282.raw, FUS01283.raw, FUS01284.raw, FUS01285.raw. (4) TAP-Cdc55Purification.xlsx. File with the proteins and peptides detected in the TAP purification Assay.

The R script used for the analysis as well as the needed data files were uploaded to the *GigaScience* repository, GigaDB [96].

## Additional files

**Additional file 1.pdf**

Workflow for SILAC analysis of PP2A-Cdc55-dependent phosphoproteome. Three different methods were used for phosphopeptide enrichment: SIMAC, TiO2, and TiSH-based approach. A detailed scheme of each methodology is presented. LC-MS/MS analysis of the eluted fractions was performed to identify and quantify the heavy/light labeled peptides. Identification and quantification was analyzed using Proteome Discoverer.

**Additional file 2.xlsx**

Summary of all the peptides identify in the three approaches. 10,069 peptides were identified: 2,696 peptides in Method 1, 2,662 peptides in Method 2, and 4,711 in Method 3.

**Additional file 3.xlsx**

Hyperphosphorylated peptides corresponding to putative PP2A-Cdc55 regulated proteins. List of the 1,260 quantified hyperphosphorylated peptides identified in our three SILAC experiments.

**Additional file 4.xlsx**

Complete list of proteins and peptides identified in the whole cell extract. List of 2,674 proteins and 27,957 peptides identified in the whole cell extract.

**Additional file 5.xlsx**

Common peptides and proteins quantified in the whole cell extract and in the hyperphosphorylated list. List of the 286 matching proteins identified in the whole cell extract (non-enriched analysis) and in our hyperphosphorylated dataset. All the matching proteins had similar protein abundance between the wild type and the *cdc55∆* mutant (heavy/light ratio >0.8 in the non-enriched analysis).

**Additional file 6.xlsx**

Common peptides and proteins found in the phosphoproteomic study. List of the hyperphosphorylated peptides and proteins found in the three different phospho-enrichment approaches corresponding to the Venn diagrams in Fig. 1F–G.

**Additional file 7.xlsx**

List of statistically significant hyperphosphorylated peptides. The 62 hyperphosphorylated peptides from the volcano plot in Fig. 1I. We identified 62 unique hyperphosphorylated peptides containing 76 unique phosphosites.

**Additional file 8.xlsx**

GO of the PP2A-Cdc55 potential substrates. The gene ontology terms of the 247 commons proteins from Fig. 1G are summarized in the Non-Clustered sheet, and the functional clustering of the GO terms is summarized in the Clustered sheet.

**Additional file 9.pdf**

String Network analysis of the 247 common hyperphosphorylated proteins. Interactions found for the 247 common proteins from Fig. 1G.

**Additional file 10.xlsx**

Detailed list of the proteins nodes described in Fig. 3 and Additional file 7. Proteins for each node and the interaction score are shown.

**Additional file 11.xlsx**

Proteins identified in two TAP-Cdc55 purification assays. List of proteins identified in the two TAP-Cdc55 pull-downs that are not found in the negative control purification. A strain without the TAP epitope was used as negative control.

**Additional file 12.xlsx**

Proteins identified in the HA-Cdc55 purifications. Proteins and peptides identified after HA-Cdc55 purification using HA-affinity columns. The eluted fractions were subjected to TiO_2_ enrichment to search for proteins that are undergoing phosphorylation modifications among the newly identified Cdc55-associated proteins. Peptide and protein modifications were obtained using the Mascot search engine.

**Additional file 13.xlsx**

Proteins and peptides identified containing a Cdc5 consensus site. List of proteins from our PP2A-Cdc55 phosphoproteome dataset (62 statistically significant phosphopeptides) containing the D/E/N-x-S/T Cdc5 polo-like kinase consensus sites. We identified 16 phosphopeptides corresponding to 16 unique proteins.

**Additional file 14.xlsx**

Common elements between PP2A^Cdc55^ targets identified in Godfrey et al. [63] and our study. A list of common proteins for each cell cycle stages is presented.

## Abbreviations

APC/C Anaphase-promoting complex; AMP Adenosine monophosphate; ATP Adenosine triphosphate; CBP Calmodulin binding protein; DOPE Discrete Optimized Protein, Energy EDTA Ethylenediaminetetraacetic acid; FDR False discovery rate; FWHM Full width at half maximum; GSK3 kinases Glycogen synthase kinase 3; H/L Heavy/Light; HA Human influenza hemagglutinin; HILIC Hydrophilic interaction liquid chromatography; IMAC Immobilized metal affinity chromatography; LC-MS Liquid chromatography-mass spectrometry; MAPK Mitogen-activated protein kinases; NMR Nuclear magnetic resonance spectroscopy; PKA protein kinase A PRIDE Proteomics IDEntifications; QTOF Quadruple Time of Flight; SGD Saccharomyces Genome Database; SIMAC sequential elution from IMAC; SV5 Simian virus 5 epitope; TFA Trifluoroacetic acid; TiO2 Titanium dioxide UV Ultraviolet.

## Supplementary Material

GIGA-D-17-00246_Original_Submission.pdf

GIGA-D-17-00246_Revision_1.pdf

GIGA-D-17-00246_Revision_2.pdf

Response_to_Reviewer_Comments_Original_Submission.pdf

Response_to_Reviewer_Comments_Revision_1.pdf

Reviewer_1_Report_(Original_Submission) -- Andrew Burgess 10/12/2017 Reviewed

Reviewer_1_Report_(Revision_1) -- Andrew Burgess 1/15/2018 Reviewed

Reviewer_2_Report_(Original_Submission) -- Peter Li 10/17/2017 Reviewed

Reviewer_2_Report_(Revision_1) -- Peter Li 1/22/2018 Reviewed

Reviewer_3_Report_(Original_Submission) -- Arminja Kettenbach 10/18/2017 Reviewed

Reviewer_3_Report_(Revision_1) -- Arminja Kettenbach 1/17/2018 Reviewed

Reviewer_3_Report_(Revision_2) -- Arminja Kettenbach 3/13/2018 Reviewed

Additional Files
