# Supplementary material for: SILAC-based phosphoproteomics reveals new PP2A-Cdc55-regulated processes in budding yeast
Source: Gigascience. 2018 May 24;7(5):giy047. doi: 10.1093/gigascience/giy047 (PMC5967524; doi:10.1093/gigascience/giy047)
Supplement: GIGA-D-17-00246_Original_Submission.pdf [file giy047_giga-d-17-00246_original_submission.pdf]

GigaScience

SILAC-based phosphoproteomics reveals new PP2A-Cdc55-regulated processes in budding yeast.

--Manuscript Draft--

**Manuscript Number:**	GIGA-D-17-00246
**Full Title:**	SILAC-based phosphoproteomics reveals new PP2A-Cdc55-regulated processes in budding yeast.
**Article Type:**	Research
**Funding Information:**	Secretaría de Estado de Investigación, Desarrollo e Innovación (BFU2013-43132-P) — Dr Ethel Queralt<br>Secretaría de Estado de Investigación, Desarrollo e Innovación (BFU2016-77975-R) — Dr Ethel Queralt<br>Secretaría de Estado de Investigación, Desarrollo e Innovación (BFU2011-27568) — Dr Ethel Queralt<br>Lundbeckfonden — Dr. Martin R Larsen<br>Villum Fonden — Dr. Martin R Larsen<br>Instituto de Salud Carlos III (13FIS037) — Dr. Silvia Barceló-Batllori<br>Instituto de Salud Carlos III (PT13/0001/0033) — Dr. Silvia Barceló-Batllori
**Abstract:**	Background: Protein phosphatase 2A (PP2A) is a family of conserved serine/threonine phosphatases involved in several essential aspects of cell growth and proliferation. PP2ACdc55 phosphatase has been extensively related to cell cycle events in budding yeast, however few PP2ACdc55 substrates have been identified. Here, we performed a quantitative mass spectrometry approach to reveal new substrates of PP2ACdc55 phosphatase and new PP2A-related processes in mitotic arrested cells. Results: We identified 626 potential PP2ACdc55 substrates involved in a broad range of mitotic processes. In addition, we validated new PP2ACdc55 substrates such as Slk19 and Lte1, involved in early and late anaphase pathways, and Zeo1, a component of the cell wall integrity pathway. Finally, we constructed docking models of Cdc55 and its substrate Mob1. We found that the predominant interface on Cdc55 is mediated by a protruding loop consisting of residues 84-90, thus highlighting the relevance of these aminoacids for substrate interaction. Conclusions: We used phosphoproteomics of Cdc55 deficient cells to uncover new PP2ACdc55 substrates and functions in mitosis. As expected, several hyperphosphorylated proteins corresponded to Cdk1-dependent substrates, although other kinases' consensus motifs were also enriched in our dataset, suggesting that PP2ACdc55 counteracts and regulates other kinases distinct from Cdk1. Indeed, Pkc1 and Cla4 kinases emerged as novel nodes of PP2ACdc55 regulation, highlighting a major role of PP2ACdc55 in membrane trafficking and cytokinesis, gene ontology terms significantly enriched in the PP2ACdc55-dependent phosphoproteome.
**Corresponding Author:**	Ethel Queralt, PhD in Biochemistry<br>Institut d'Investigacio Biomedica de Bellvitge<br>Barcelona, Barcelona SPAIN
**Corresponding Author Secondary Information:**	
**Corresponding Author's Institution:**	Institut d'Investigacio Biomedica de Bellvitge
**Corresponding Author's Secondary Institution:**	
**First Author:**	Barbara Baro
**First Author Secondary Information:**	

**Order of Authors:**	Barbara Baro
	Soraya Jativa
	Ines Calabria
	Judith Vinaixa
	Joan-Josep Bech-Serra
	Carolina deLaTorre
	Joao Rodrigues
	Maria Luisa Hernaez
	Concepción Gil
	Silvia Barceló-Batllori
	Martin R Larsen
	Ethel Queralt, PhD in Biochemistry
**Order of Authors Secondary Information:**	
**Opposed Reviewers:**	
**Additional Information:**	
**Question**	**Response**
Are you submitting this manuscript to a special series or article collection?	No
**Experimental design and statistics**<br><br>Full details of the experimental design and statistical methods used should be given in the Methods section, as detailed in our Minimum Standards Reporting Checklist. Information essential to interpreting the data presented should be made available in the figure legends.<br><br>Have you included all the information requested in your manuscript?	Yes
**Resources**<br><br>A description of all resources used, including antibodies, cell lines, animals and software tools, with enough information to allow them to be uniquely identified, should be included in the Methods section. Authors are strongly encouraged to cite Research Resource Identifiers (RRIDs) for antibodies, model organisms and tools, where possible.<br><br>Have you included the information requested as detailed in our Minimum Standards Reporting Checklist?	Yes

**Availability of data and materials**<br><br>All datasets and code on which the conclusions of the paper rely must be either included in your submission or deposited in publicly available repositories (where available and ethically appropriate), referencing such data using a unique identifier in the references and in the "Availability of Data and Materials" section of your manuscript.<br><br>Have you have met the above requirement as detailed in our Minimum Standards Reporting Checklist?	Yes

Manuscript Click here to download Manuscript Baro.docx

SILAC-based phosphoproteomics reveals new PP2A-Cdc55-regulated processes in budding yeast.

Barbara Baro[1]*, Soraya Játiva[1], Inés Calabria[1$], Judith Vinaixa[1], Joan-Josep Bech-Serra[2], Carolina deLaTorre[2], João Rodrigues[3], María Luisa Hernáez[4], Concha Gil[4], Silvia Barceló-Batllori[2], Martin R Larsen[5] and Ethel Queralt[1#]

Cell Cycle Group, Cancer Epigenetics and Biology Program, Institut d'Investigacions Biomèdica de Bellvitge (IDIBELL), L'Hospitalet de Llobregat, Barcelona, Spain[1]; IDIBELL Proteomics Unit, Institut d'Investigacions Biomèdica de Bellvitge, L'Hospitalet de Llobregat, Barcelona, Spain[2]; Structural Biology Department, School of Medicine, Stanford, California, USA[3]; Proteomics Unit, Parque Científico de Madrid, Facultad de Farmacia, Universidad Complutense de Madrid, Madrid, Spain[4]; Department of Biochemistry and Molecular Biology, Odense M, Denmark[5]

Running Head: Targets of PP2A^{Cdc55} phosphatase

* Present address: Division of Infectious Diseases, Pediatrics Department, School of Medicine, Stanford, California, USA.

[$] Present address: Genomics Unit, Medical Research Institute La Fe, Valencia, Spain.

#Address correspondence to Ethel Queralt: equeralt@idibell.cat.

Text word count: 12374

Key words: mitosis, PP2A^{Cdc55} phosphatase, Pkc1, Cla4, mitotic exit network (MEN), Mob1, phosphoproteomics, SILAC.

Abstract

Background: Protein phosphatase 2A (PP2A) is a family of conserved serine/threonine phosphatases involved in several essential aspects of cell growth and proliferation. PP2A^{Cdc55} phosphatase has been extensively related to cell cycle events in budding yeast, however few PP2A^{Cdc55} substrates have been identified. Here, we performed a quantitative mass spectrometry approach to reveal new substrates of PP2A^{Cdc55} phosphatase and new PP2A-related processes in mitotic arrested cells. Results: We identified 626 potential PP2A^{Cdc55} substrates involved in a broad range of mitotic processes. In addition, we validated new PP2A^{Cdc55} substrates such as Slk19 and Lte1, involved in early and late anaphase pathways, and Zeo1, a component of the cell wall integrity pathway. Finally, we constructed docking models of Cdc55 and its substrate Mob1. We found that the predominant interface on Cdc55 is mediated by a protruding loop consisting of residues 84-90, thus highlighting the relevance of these aminoacids for substrate interaction. Conclusions: We used phosphoproteomics of Cdc55 deficient cells to uncover new PP2A^{Cdc55} substrates and functions in mitosis. As expected, several hyperphosphorylated proteins corresponded to Cdk1-dependent substrates, although other kinases' consensus motifs were also enriched in our dataset, suggesting that PP2A^{Cdc55} counteracts and regulates other kinases distinct from Cdk1. Indeed, Pkc1 and Cla4 kinases emerged as novel nodes of PP2A^{Cdc55} regulation, highlighting a major role of PP2A^{Cdc55} in membrane trafficking and cytokinesis, gene ontology terms significantly enriched in the PP2A^{Cdc55}-dependent phosphoproteome.

Background

Protein phosphorylation is a key regulatory mechanism of protein function that governs cell cycle progression (reviewed in (1)). The highly conserved and specific family of cyclin-dependent serine/threonine kinases, the Cdks, were considered the main component of the cell cycle control system once they were discovered. Nowadays, it has become clear that the opposing phosphatases also play a key role in setting the net phosphorylation state of each substrate, thereby being the other side of the coin controlling phosphorylation waves during cell cycle progression. Cdk1-cyclin activity progressively increases as the cell cycle progresses, reaching its maximum in metaphase. At the end of mitosis, high Cdk1 activity needs to return to lower levels in order to enter into a new G1 phase, and activation of Cdk1-counteracting phosphatases is required for this transition.

Type 2A phosphatases (PP2A) is a family of conserved protein serine/threonine phosphatases involved in several essential aspects of cell growth and proliferation. PP2A is a major Cdk1-counteracting phosphatase during cell cycle progression, which works solely as a multimeric enzyme (2). The PP2A core enzyme consists of a scaffold subunit and a catalytic subunit. The heterodimeric complex interacts with a variable regulatory subunit (B subunit) to assemble into a holoenzyme. Although highly conserved within the same family, these regulatory subunits share little sequence similarity across families, and their expression levels vary greatly in different cell types and tissues (3). Several studies have shown that PP2A regulatory subunits confer exquisite substrate specificity to PP2A holoenzymes *in vivo* (4–12).

PP2A is highly conserved from yeast to humans. Knockdown of either the catalytic or a subset of regulatory subunit genes of PP2A holoenzymes results in unviable cells (13–17). In *S. cerevisiae*, the PP2A scaffold subunit is known as Tpd3. The catalytic subunit of the core enzyme is either Pph21 or Pph22, two highly homologous proteins sharing 95% sequence identity (18, 19). Mutation of both *PPH21* and *PPH22* eliminates the majority of PP2A activity in the cell and drastically reduces growth. Strains lacking *PPH21*, *PPH22*, and a third related gene, *PPH3*, are completely unviable (19). The regulatory subunits comprise Cdc55 (B-type in vertebrates), Rts1 (B'-type in vertebrates) and the predicted B-subunit Rts3. In this work, we refer to Tpd3, Pph21 or Pph22, and Cdc55 holoenzyme as $PP2A^{Cdc55}$.

$PP2A^{Cdc55}$ and its mammalian homolog, $PP2A^{B55}$, have been extensively studied for their role in mitotic entry regulation (reviewed in (20)). The regulatory axis of Greatwall and PP2A inhibitors, endosulfins (Igo1/2 in budding yeast), govern mitotic entry in both yeast and in higher eukaryotes (21, 22), illustrating the strong conservation of PP2A regulatory mechanisms across eukaryotes. One of the first known functions of $PP2A^{Cdc55}$ in cell-cycle regulation was its key role affecting Swe1 and Mih1 activity at the G2/M transition (23–28) (Wee1 and Cdc25 in vertebrates). More recently, signals regarding the status of membrane traffic have been shown to be integrated into mitosis progression through $PP2A^{Cdc55}$ via a signaling cascade that includes Rho1, Pkc1 and Zds1/2. Pkc1 binds to $PP2A^{Cdc55}$-Zds1/2, which directly controls the phosphorylation states of Mih1 and Swe1 (29–34). However, $PP2A^{Cdc55}$ substrates and functions during mitotic exit are less understood, since another phosphatase, Cdc14, which is essential and specifically activated at anaphase-onset, has been considered the principal Cdk1-counteracting phosphatase during mitotic exit in

budding yeast. In contrast, in vertebrates cells, although *CDC14* homologues are present (35), their functions seem less conserved (36), and PP2A-B55 and PP1 phosphatases are considered the major Cdk1-counteracting phosphatases during mitotic exit (37, 38). Indeed, yeast $PP2A^{Cdc55}$ has also been shown to play a major role during mitotic exit. $PP2A^{Cdc55}$ counteracts Cdk1-dependent phosphorylation of Net1, which is crucial for Net1-Cdc14 dissociation (39). Zds1/2 proteins cooperate with separase to downregulate $PP2A^{Cdc55}$ at anaphase-onset (40, 41) which leads to Cdc14 activation and release. Thus, Zds1/2 are common $PP2A^{Cdc55}$ modulators, participating in both entry and exit from mitosis. It has recently been described that $PP2A^{Cdc55}$ downregulation in anaphase also initiates the Mitotic Exit Network (MEN) by dephosphorylating the MEN components Bfa1 and Mob1 (42). In addition, $PP2A^{Cdc55}$ downregulation at anaphase-onset facilitates separase proteolytic activity towards Scc1, which triggers sister-chromatid segregation (43). Finally, $PP2A^{Cdc55}$ as well as its homologue, PP2A-B55, has been shown to counteract Cdk1-dependent phosphorylation of APC/C during mitosis (44–47). In conclusion, $PP2A^{Cdc55}$ is also a major Cdk1-counteracting phosphatase during mitotic exit in budding yeast.

Quantitative mass spectrometry has been used to identify Cdk-dependent phosphorylation sites in a large number of substrates *in vivo*, by comparing the phosphoproteome of wild-type cells and Cdk1 defective cells (48, 49). More recently, a global analysis of Cdc14 dephosphorylation sites was performed using a similar approach (50, 51). In this study, we performed a systematic quantitative phosphoproteomic analysis of $PP2A^{Cdc55}$ deficient cells to identify novel $PP2A^{Cdc55}$ substrates and regulated processes. Since drug inhibition by Okadaic acid in budding yeast only works at high concentration, which also inhibits other

Ser/Thr phosphatases, and due to the specificity that the regulatory subunits confer to PP2A (52), in our approach we used a *cdc55* deletion mutant to explore the $PP2A^{Cdc55}$-dependent phosphoproteome. Hence, *cdc55* deficient cells lack $PP2A^{Cdc55}$ activity but not the other PP2A complexes, $PP2A^{Rts1}$ or $PP2A^{Rts3}$. With this approach, we expected to decipher new pathways specifically regulated by the $PP2A^{Cdc55}$ phosphatase. We identified both known and potentially new substrates for $PP2A^{Cdc55}$ as well as their phosphorylation sites.

While preparing this manuscript, two SILAC-based studies targeting $PP2A^{Cdc55}$ (53) and the mammalian, PP2A-B55 (54), were published. By comparing the phosphorylation status of Cdk1 substrates in the absence of $PP2A^{Cdc55}$ at different cell cycle phases (G1, S and M)(53), they deciphered how $PP2A^{Cdc55}$ contributes to determining the progressive phosphorylation of Cdk1 substrates. In contrast, our study focused on metaphase-arrested cells, and we considered not only the Cdk1-counteracted substrates but all Cdc55-dependent phosphorylation sites for downstream analysis. Indeed, we found a major set of peptides containing Cdk1 consensus sites, in agreement with previous data showing that $PP2A^{Cdc55}$ mostly counteracts the phosphorylation of Cdk1 targets. But, interestingly, we also identified other kinase consensus sequences corresponding to ERK/MAPK kinases, Cdc5 Polo kinase and AGC kinases; suggesting that $PP2A^{Cdc55}$ counteracts other kinases apart from Cdk1, and/or regulates their activities. Finally, we were able validate up to 9 targets by protein-protein interactions and/or by western blot, which strongly support the validity of our study. We assume that the substrates of the $PP2A^{Cdc55}$ phosphatase identified might not be all direct targets; however, as well as this, our work also uncovered valuable new PP2A-related processes. In fact, gene ontology analysis of our phosphoproteome study identified several processes related to mitosis, actin cytoskeleton organization, budding and cytokinesis. Budding impinges a dramatic re-arrangement of the cell structure, and

morphogenesis changes, GO categories that were also found in our study. In addition, we identified proteins related to osmotic stress and nutrient response. Thus, $PP2A^{Cdc55}$ phosphatase seems to play a key role in sensing several cues of the environmental conditions, cell growth, cell polarity and cell structure, and integrating them to regulate the cell cycle.

Data description

To screen for potential new substrates of the $PP2A^{Cdc55}$ phosphatase during mitosis, we performed a quantitative phosphoproteomic analysis based on the *S*table *I*sotope *L*abelling by *A*mino Acids in *C*ell Culture (SILAC) technique. To study the $PP2A^{Cdc55}$-dependent phosphoproteome, we compared the phosphoproteome of a wild-type strain and a *cdc55Δ* mutant strain, which lacks the activity of $PP2A^{Cdc55}$ but not other PP2A complexes. The PP2A regulatory subunits confer substrate specificity to PP2A. Therefore, in our approach we specifically studied the $PP2A^{Cdc55}$ and no other PP2A complexes (with Rts1 or Rts3). To minimize compensatory mutations that might accumulate over time in the gene deletion strain, we freshly prepared the *cdc55Δ* mutant. Wild-type and *cdc55Δ* cells were grown in methionine-free minimum media containing $^{13}C_6$-lysine and -arginine (heavy) or unmodified arginine and lysine (light), respectively. Both strains expressed *CDC20* under the control of the repressible *MET3* promoter and were synchronized at the metaphase-to-anaphase transition by adding methionine to the media, which causes Cdc20 depletion. At the time of harvesting, more than 95% of cells in each culture were arrested in metaphase. Protein extracts were prepared as described in methods.

We used three different strategies to enrich for phosphopeptides: SIMAC, TiO_2 and TiSH-based (TiO_2-SIMAC-HILIC). A schematic representation of the different strategies used is shown in Additional file 1. Analysis of the heavy/light labelled phosphopeptides was performed by LC-MS/MS (see methods for more details). Global analysis of the data led to the identification of 10,069 peptides, including 4,186 phosphopeptides. Only peptides identified with high confidence (< 1% FDR) were used for further analysis. The mass spectrometry proteomics data have been deposited to the ProteomeXchange Consortium with the dataset identifier PXD007613.

Analyses

Large-scale identification of PP2A^{Cdc55}-dependent phosphoproteome in metaphase-arrested cells

To study the PP2A^{Cdc55}-dependent phosphoproteome, we selected the hyperphosphorylated peptides according to the filtering parameters described in methods (Fig. 1A). Analysis of this subset of data led to the quantification of 628 phosphoproteins, represented by 1,260 quantified hyperphosphorylated peptides. The hyperphosphorylated peptides selected and statistical parameters used are shown in Additional file 2. In addition, already known PP2A^{Cdc55} substrates such as Net1, Mob1, Gis1 and Whi5 were identified as being hyperphosphorylated in the *cdc55*Δ mutant, which strongly supports the validity of our approach (39, 42, 55, 56).

Since phosphorylation changes measured by the heavy/light ratio could be affected by changes in protein abundance due to absence of Cdc55, we analyzed one aliquot of the protein extract without phosphopeptide enrichment (see methods) and determined the heavy/light ratio to account for protein abundance. We could quantify a total of 18,592

peptides, of which 15,640 peptides contained a heavy/light ratio >0.8 and 2,952 peptides which had a heavy/light ratio <0.8. Therefore, only 15.8% of the peptides had reduced protein abundance due to the absence of Cdc55 (Fig. 1B). In fact, we identified 286 matching proteins to the selected hyperphosphorylated dataset (see Additional file 3), and all of them had similar protein abundance between the wild type and the *cdc55*Δ mutant (heavy/light ratio >0.8 in non-enriched analysis). Therefore, we conclude that most of the hyperphosphorylated proteins selected with a heavy/light ratio <0.75 correspond to phosphorylation changes and not to protein abundance changes. Nevertheless, we cannot rule out that, for some proteins, changes in protein abundance might affect the heavy/light ratio, since we could not identify all the hyperphosphorylated peptides in the non-enriched fraction.

The overlap of hyperphosphorylated peptides and phosphoproteins found in the three different approaches are shown by Venn diagrams (Fig. 1E-F). Common proteins found in the three experiments are summarized in Fig. 1G and the common peptides are shown in Additional file 4. The volcano plot of the common phosphopeptides showed a higher amount of hyperphosphorylated peptides compared to the hypophosphorylated ones (Fig. 1H), in accordance with enrichment in PP2A^{Cdc55} potential substrates. However, since SIMAC, TiO2 and TiSH-based enrichments have different capacity and specificity, common hits are considered hyperphosphorylated peptides which showed similar performance in the different purification protocols used, rather than being more likely to be PP2A^{Cdc55} substrates. Indeed, each approach uncovered a unique subset of hyperphosphorylated peptides useful for downstream analysis.

We next analyzed the phosphorylated residues found in the hyperphosphorylated peptides dataset using the non-enriched sample as background. From 1,375 unique phosphosites

identified, 78.25% corresponded to phosphoserine, 20.65% to phosphothreonine and 1.09% to phosphotyrosine (Fig. 1D), which is consistent with PP2A^{Cdc55} being a Ser/Thr phosphatase. This phosphosite distribution is also consistent with the recently reported PP2A^{Cdc55} preference for threonine residues in mitotic substrates (53, 54), since the global *S. cerevisiae* phosphoproteome consists of only 13-15% phosphothreonine. Our next analysis regarding phosphomotifs enriched in the *cdc55*Δ mutant also highlighted PP2A^{Cdc55} preference for phosphothreonines (see below).

PP2A^{Cdc55} dependent phosphorylation sites of known kinases.

We were interested in studying the kinases counteracted by PP2A^{Cdc55}. It has been shown that PP2A^{Cdc55} phosphatase can counteract Cdk1 phosphorylation (39) and Cdc5 phosphorylation (42, 43). We found that 32.95% of the phosphosites correspond to SP/TP (minimum Cdk1 consensus sequence), consistent with PP2A^{Cdc55} mainly counteracting Cdk1 phosphorylation (Fig. 1D).

In order to identify consensus phosphorylation sites of other known protein kinases, enriched sequence motifs surrounding the phosphosites in the hyperphosphorylated dataset were analyzed via Motif-X (57). For this analysis, only phosphorylated residues identified with high confidence were considered (peptides with a pRS probability > 95%; see Additional file 5). The 721 unique hyperphosphorylated peptides contained 562 unique phosphomotifs. As expected, the most represented motif found was S-P (Fig. 2A), present in 23.95% of the dataset, which corresponds to the minimum consensus site of Pro-directed kinases, such as ERK1, p38MAPKs, Cdk1, Cdk2, Cdk4 and Cdk5 (58). Interestingly, the second phosphorylation consensus sequence found was R-x-x-S of AGC kinases, which include the PKC, PKA, Sch9, Ypk1 and Ypk2 kinases. This motif was present in 9.58% of

the dataset. With a similar abundance, we found S-x-x-S and T-P motifs (9.29% and 8.99%, respectively). Finally, we found the motif S-x-x-E, one of the consensus sites described for polo kinase-dependent phosphorylation, present in 5.16% of the dataset. Within this consensus site we can infer the D/E/N-x-S motif described for the budding yeast polo-like kinase Cdc5 (59).

Motif sequences, their scores and fold increase are shown in Fig 2B. Interestingly, T-P motif presented the highest fold-increase, followed by R-x-x-S-x-x-S and S-P motifs. Our results suggest a greater regulation of T-P sites over S-P sites by PP2A^{Cdc55} in mitotic cells, as recently reported (53, 54). The motifs uncovered also suggest PP2A^{Cdc55} could counteract other kinases apart from Cdk1 and Cdc5 Polo-like kinase. Several of these kinases are also found hyperphosphorylated in our study, as well as some substrates of these newly identified PP2A^{Cdc55}-counteracted kinases, suggesting that PP2A^{Cdc55} could directly regulate their kinase activity (Table 1).

On the other hand, Cdk1-dependent phosphoproteome was uncovered in a similar study, where approximately 314 proteins containing Cdk1 consensus sites were identified as likely Cdk1 targets in budding yeast (48). Since Cdk1 is the main kinase counteracted by PP2A^{Cdc55} phosphatase, we compared our list of potential PP2A^{Cdc55} substrates containing the S/T-P motif to the Cdk1-dependent data set and we found 74 proteins that were common in both datasets (Fig. 2C). These common proteins corresponded to GO processes such as cell cycle and mitotic cell cycle (Fig. 2C *right panel*) as expected, and they are more likely to be regulated by both Cdk1 and PP2A$^{Cdc55.}$

Novel roles for PP2A^{Cc55} phosphatase in cytokinesis and endocytosis

Functional clustering of proteins that displayed enhanced phosphorylation in our dataset is

presented in Additional file 6 and summarized in Table 2. We found a strong enrichment for cell cycle related functional categories such as cell cycle, mitotic cell cycle, cell growth, budding, cell polarity, actin cytoskeleton, cytokinesis and endocytosis. Most of these processes are related to mitosis events, consistent with a PP2A^{Cdc55} role in mitosis and our analysis of mitotic arrested cells.

PP2A^{Cdc55} has been recently shown to monitor membrane trafficking and bud growth, integrating several cues to the mitotic entry regulators Swe1 and Mih1 (32) (Wee1 and Cdc25 in mammals). Budding impinges a dramatic re-arrangement of the cell wall and cell morphogenesis, GO categories that were found in our study. Interestingly, we found components of the cell wall integrity pathway, the Pkc1, Bck1, Ypk1/2 and Pkh1 kinases, and Zeo1. Indeed, Pkc1 consensus motif was enriched in our PP2A^{Cdc55}-dependent phosphosites. Moreover, we have been able to identify a physical interaction between Zeo1 and Cdc55 (see below), suggesting that Zeo1 is likely to be a PP2A^{Cdc55} substrate. We also identified other proteins related to budding such as Bud3, Bud6, Gin4 and Nap1. Interestingly, many proteins required for cytokinesis like Inn1, Boi1, Shs1, Bni4, Cdc11, Cdc12, Cdc3 and Iqg1 were also found among the PP2A^{Cdc55}-dependent phosphoproteome, as well as proteins involved in the general organization of the actin cytoskeleton like Sla1, Bud6, Bni1 and Spa2. On the other hand, we also found proteins related to vesicle-mediated transport and endocytosis. Control of membrane structures, cell membrane trafficking and endocytosis have recently been linked to cytokinesis processes (60) and mammalian homolog, PP2A-B55, has been related to the reformation of the nuclear envelope and the Golgi apparatus during telophase (38). Finally, we also identified proteins related to osmotic stress and nutrient response. Thus, PP2A^{Cdc55} phosphatase seems to play a key role sensing several cues of the environmental conditions, cell growth and cell

structure, and integrating them into cell cycle regulation. In our screen, we also found proteins related to signal transduction, transcription, chromatin organization and organelle organization, all processes that are monitored and/or coordinated within the cell cycle (see Additional file 6).

A String Network Analysis of our hyperphosphorylated proteins is showed in Additional file 7. We plotted the number of interactions found for each protein, and we identified 6 proteins with more than 15 interactions: Cdc28, Cla4, Pkc1, Snf1, Stb1 and Swi4 (Fig. 3). Cdc28 and Pkc1 (32, 33) had been previously linked with $PP2A^{Cdc55}$. Pkc1 is a serine/threonine kinase involved in cell wall organization that has recently been related to $PP2A^{Cdc55}$, as it controls the binding of Igo1/2 proteins to PP2A (34). As we just mentioned, we uncovered several proteins from the cell wall organization pathway, and the Pkc1 kinase consensus site was found enriched in our $PP2A^{Cdc55}$ dependent phosphoproteome.

On the other hand, Snf1 is an AMP-activated serine/threonine kinase involved in the regulation of transcription of glucose-repressed genes. It regulates filamentous growth in response to starvation. Stb1 regulates the MBF-G1/S specific transcription factor; while Swi4 together with Swi6 forms the second G1/S specific transcription factor, SBF. Therefore, Snf1, Stb1 and Swi4 have essential roles in G1, and are potential regulation nodes of $PP2A^{Cdc55}$, highlighting the importance of this phosphatase in G1 regulation, in agreement with recent studies uncovering G1-related functions (55, 56, 61, 62).

Finally, Cla4 also emerged as a potential node of $PP2A^{Cdc55}$ regulation in our study. Strikingly, Cla4 is a PAK kinase that regulates septin ring assembly during cytokinesis and vacuole inheritance. Cla4 and the related Ste20 kinase consensus motif were found

enriched among our $PP2A^{Cdc55}$ dependent phosphoproteome and many proteins related to cytokinesis were identified. Altogether, our results suggest that Cla4 and $PP2A^{Cdc55}$ might have more related functions in cytokinesis than previously anticipated.

Validation of novel $PP2A^{Cdc55}$ substrates in mitosis

cdc55Δ cells exhibit elevated tyrosine 19 phosphorylation on Cdk1 due to dysregulation of Swe1 and/or Mih1 (25, 27, 29, 63). We first confirmed that we could detect this hyperphosphorylation of Cdk1-Y19 in *cdc55Δ* cells in our phosphoproteome screen (VGEGTyGVVYK, Y6 phosphoRS site probability > 89%).

We next searched for already known $PP2A^{Cdc55}$ substrates (Fig. 4A), as we previously published an extended study about Net1 being a $PP2A^{Cdc55}$ substrate and its functional relevance for mitotic exit regulation (39). Net1 was identified as being hyperphosphorylated in the *cdc55*Δ mutant, suggesting our approach to broadly identify substrates worked. In addition, Mob1 protein was identified in this phosphoproteomic study as a low confidence phosphopeptide, which we recently validated as a new $PP2A^{Cdc55}$ substrate and demonstrated functional relevance for MEN activation (42). Based on that result, we looked for other MEN components in our $PP2A^{Cdc55}$-dependent phosphoproteome, and we found Lte1 hyperphosphorylated in the *cdc55*Δ mutant. We further explored Lte1 phosphorylation at the metaphase to anaphase transition (Fig. 4B). Wild-type and *cdc55*Δ cells were arrested in metaphase by Cdc20 depletion and released into synchronous anaphase by Cdc20 re-introduction. In wild-type cells, Lte1 was dephosphorylated in anaphase and transition to G1 (M/G1). In contrast, Lte1 was hyperphosphorylated in *cdc55*Δ cells at the indicated times, suggesting is likely to be a $PP2A^{Cdc55}$ substrate. Native protein extracts from metaphase samples were treated with

alkaline phosphatase as a control of phosphorylation. Additional MEN components, Cdc14 and Kin4, were also identified in our phosphoproteome analyses as putative new substrates of PP2A^{Cdc55} (Additional file 1), suggesting a closer regulation of the whole MEN pathway by PP2A^{Cdc55} phosphatase.

On the other hand, one component of the FEAR pathway was also identified in our PP2A^{Cdc55}-dependent phosphoproteome, Slk19, which is a kinetochore-associated protein involved in chromosome segregation and Cdc14 release. We explored Slk19 protein modifications in the metaphase to anaphase transition as we had done for Lte1. In wild type cells, Slk19 is phosphorylated in metaphase and, upon anaphase entry, undergoes cleavage. In contrast, Slk19 was hyperphosphorylated in *cdc55*Δ cells throughout anaphase, and although it underwent cleavage, Slk19 showed an altered migration pattern of the cleaved form. This result suggests that PP2A^{Cdc55} is required to dephosphorylate Slk19.

In addition, Rts1 the second regulatory subunit of PP2A^{Cdc55} was also identified in our phosphoproteome analysis. PP2A^{Rts1} is located at the centromeres during mitosis and prevents cohesin cleavage by separase (64), and it is also required for cell size control (65). Rts1 was dephosphorylated in M/G1 in wild-type cells (Fig. 4B). In contrast, Rts1 was hyperphosphorylated in *cdc55*Δ cells at the indicated times. Native protein extracts from metaphase samples were treated with alkaline phosphatase as control. These results indicate that Rts1 is hyperphosphorylated in the absence of PP2A^{Cdc55}, suggesting that PP2A^{Cdc55} is required to dephosphorylate Rts1.

Zeo1 and other potential PP2A^{Cdc55} substrates interact with the PP2A^{Cdc55} phosphatase *in vivo*

Finally, we used Cdc55 pull-down strategies to further validate new potential substrates of $PP2A^{Cdc55}$ and further explore specific binding partners of this phosphatase. We first used tandem affinity purification (TAP) to find new Cdc55 interactors, using a strain expressing a TAP-epitope tagged Cdc55 (TAP-Cdc55). TAP involves fusion of the TAP epitope (protein A from *Staphylococcus aureus* and the calmodulin binding peptide [CBP] arranged in tandem and separated by a TEV protease cleavage site) to the target protein of interest. The fusion protein and their associated components were then recovered by two rounds of affinity purifications. Eluted fractions were then directly processed by high sensitive LC-MS/MS methods. A strain without the TAP epitope was used as control. The peptides identified in the TAP-Cdc55 pull-down that are not found in the negative control purification are considered novel Cdc55 associated proteins (Additional file 8). Among them, 4 proteins Zeo1, Apa1, Dnm1 and Set1 were also found hyperphosphorylated in our $PP2A^{Cdc55}$-dependent phosphoproteome (Fig. 4C), suggesting they are likely to be $PP2A^{Cdc55}$ substrates.

We next performed a second Cdc55 purification using HA-Cdc55 tagged strain and HA-affinity columns. The eluted fractions were subjected to TiO_2 enrichment to search for proteins that are undergoing phosphorylation modifications among the newly identified Cdc55 associated proteins. The enriched peptides were subjected to LC-MS/MS. Peptides identified are shown in Additional file 9. Among them, Psh1, Tgl1, Hos3 and Sro9 were identified as Cdc55-interacting proteins. Peptide and protein modifications were obtained using the Mascot search engine. Interestingly, Tgl1 and Psh1 were also found in our quantitative phosphoproteomic study of potential $PP2A^{Cdc55}$ substrates (Fig. 4D). Considering that those proteins interact physically with $PP2A^{Cdc55}$ and are found hyperphosphorylated in *cdc55Δ* cells, they are likely new $PP2A^{Cdc55}$ substrates.

We observed little overlap between our SILAC study with the pull-down experiments. This is consistent with the long-held notion that kinase-substrate interactions are commonly weak and transient, thus difficult to detect by purification-based protein interaction screens.

Docking models of PP2A^{Cdc55} and Mob1 highlight potential binding interfaces for Cdc55 and Mob1

To explore the interaction surface of Cdc55 and its Cdk1-dependent substrates, we performed rigid-body computational docking using HADDOCK (66) (version 2.2,). Except for the previously validated substrate Mob1 (42), none of the other substrates have structural data for regions with Cdc55-dependent phosphosites. As such, we built a homology model of Cdc55 based on the crystal structure of the mammalian homologue B55 and used the published crystal structure of Mob1 to build 100.000 models of the Cdc55/Mob1 complex, using knowledge of a Tau binding region on B55 to restrict the search space of the docking calculations on the Cdc55 surface.

The best 10.000 models, ranked by intermolecular energy, cluster into 437 representative binding poses that show a smooth distribution of Mob1 across the surface of the β-propeller of PP2A (Figure 5A). Filtering these models for those where Mob1 adopts a binding pose compatible with dephosphorylation by the catalytic subunit of PP2A, measured by the distance between a known phosphosite (S80) and the proton donor on PP2A (H118), narrows down the possible interaction nodes to 294 models (12 clusters) with a very similar interaction surface (Figure 5B). In these models, the predominant interface on Cdc55 is mediated by a protruding loop consisting of residues 84-90, which were shown to be critical for Tau binding and more recently to the binding of mitotic substrate PRC1;

therefore, the Cdc55 residues interacting with its substrates seem to be conserved. This is shown more clearly by a statistical analysis of per-residue interface propensities where the residues 84-90 (marked in red) appeared concentrated in the more frequent interfaces (Figure 5C). On Mob1, there is no such conserved narrow interface (represented as red residues broadly spread throughout the interphases), even among the binding poses consistent with the dephosphorylation function, although one face of the protein seems to be more favorable for interaction (Figure 5D). Interestingly, most of these models are located in between the regulatory B55 subunit and the catalytic subunit of PP2A, which would be compatible with an open-close conformational change of the scaffold subunit. Indeed, a substantial degree of flexibility of the scaffold subunit has been observed upon formation of the core enzyme alone (67).

Discussion

Mitotic exit depends on phosphatase activation in all organisms studied so far. $PP2A^{Cdc55}$ is a major Cdk1-counteracting phosphatase during cell cycle progression and a principal mitotic regulator. To uncover new $PP2A^{Cdc55}$ targets and functions during mitosis, we depleted *CDC55* in budding yeast and screened for hyperphosphorylated peptides enriched in metaphase-arrested cells in a quantitative SILAC-based approach. Non phospho-enriched control samples indicated that most of the phosphorylation changes found can be attributed to $PP2A^{Cdc55}$ inactivation and not to changes in protein abundance in the *cdc55Δ* mutant. Our dataset is consistent with $PP2A^{Cdc55}$ being a serine/threonine phosphatase and having a major role in counteracting Cdk1 activity, since S/T-P sites were the most abundant motif enriched in the absence of Cdc55. Interestingly, although phosphorylated serines were more

abundant, threonines showed the most dramatic fold-increase, in agreement with published studies showing this phosphatase has a threonine preference. Recently, the threonine preference of $PP2A^{Cdc55}$ has been proposed to determine late Cdk1 substrates (53, 54).

Although the increased phosphorylation of the proteins identified in the *cdc55Δ* mutant is either a direct or indirect effect of $PP2A^{Cdc55}$ inactivation, new regulated $PP2A^{Cdc55}$– processes can be discovered. We identified several kinases hyperphosphorylated in the absence of $PP2A^{Cdc55}$, as well as some of their substrates, suggesting that processes regulated by these kinases are potentially regulated by $PP2A^{Cdc55}$ phosphatase as well.

In addition to Cdk1 consensus sites, we found other kinase consensus motifs enriched in the Cdc55-dependent phosphoproteome. It has been shown that Cdc5 kinase phosphorylation of Scc1 (43) and Bfa1 is counteracted by $PP2A^{Cdc55}$ phosphatase (42). In accordance, we identified a group of proteins containing the proposed Cdc5 polo-like kinase consensus sites (D/E/N-x-S/T) (Additional file 10). Strikingly, we found the R-X-X-S phosphorylation motif to be enriched in the absence of Cdc55. This motif corresponds to the consensus phosphorylation motif of Pkc1, which also emerged as a node of interactions in the $PP2A^{Cdc55}$-dependent phosphoproteome.

$PP2A^{Cdc55}$ has been shown to integrate membrane growth into mitosis regulation via Rho1 and Pkc1 (32–34), regulators of the cell wall integrity pathway. Indeed, kinases of this pathway like Pkc1, Bck1, and the closely related Pkh1, Ypk1 and Ypk2, were found hyperhosphorylated in the absence of Cdc55. Regulation of Cdc55 activity by Pkc1 phosphorylation in the context of blocking membrane trafficking has also been uncovered (33). Thus, mutual regulation of Pkc1 and Cdc55 seems to occur and they might share

several substrates. Interestingly, we found Zeo1, an upstream negative regulator of the cell integrity pathway, to be hyperphosphorylated in the absence of Cdc55 phosphatase. In addition, we showed that Cdc55 and Zeo1 potentially interact through co-purification assays, thus, we conclude Zeo1 is likely a new $PP2A^{Cdc55}$ substrate.

On the other hand, the yeast casein kinase Yck2 was identified in our phosphoproteome screening and has been described to present a genetic interaction with Cdc55 (68). Yck2 shows cell cycle-specific localization to sites of polarized growth and it is required for proper septin organization and cytokinesis, functional groups identified in our GO analysis (69).

Moreover, the PAK kinase phosphorylation motif R-R-x-S (a subset of R-x-x-S) was also enriched in the absence of phosphatase $PP2A^{Cdc55}$ and two Pak-like kinases, Ste20 and Cla4, were found hyperphosphorylated (as well as their identified substrates) in the $PP2A^{Cdc55}$-dependent phosphoproteome. Ste20 and Cla4 have been linked to cytokinesis (60, 70–74) and we identified Cla4 as a node of interactions in the $PP2A^{Cdc55}$-dependent phosphoproteome, suggesting a functional link between Cla4 and $PP2A^{Cdc55}$. Indeed, Nap1 a septin regulator in fission yeast is phosphorylated by Cla4 and dephosphorylated by PP2A (75) indicating that PP2A counteracts Cla4 phosphorylations. Altogether, we conclude that $PP2A^{Cdc55}$ could counteract other kinases separate from to Cdk1 and Cdc5, like Pkc1 and Cla4, as well as regulate their activity.

In previous studies, we identified a dual regulation of the Mitotic Exit Network (MEN) by $PP2A^{Cdc55}$ phosphatase, which dephosphorylates Bfa1 and Mob1. Here, we found that other MEN components were hyperphosphorylated in the Cdc55-dependent phosphoproteome, and we validated Lte1 as a likely substrate of $PP2A^{Cdc55}$ phosphatase. Thus, $PP2A^{Cdc55}$

seems to closely regulate the MEN pathway, by dephosphorylating other elements apart of Bfa1 and Mob1. In addition, we validated Slk19, a component of the Cdc14 early anaphase release (FEAR) pathway, as likely PP2A^{Cdc55} substrates during mitotic exit, as well as other potential substrates Apa1, Dnm1, Set1, Psh1 and Tgl1 by co-purification with Cdc55. To better understand how PP2A^{Cdc55} interacts with its substrates, we built computational docking models of Cdc55 with its recently described Cdk1-dependent substrate, Mob1. Interestingly, residues 84-90, located at the Cdc55 groove structure, were predicted to interact with Mob1. This same interface has been shown to be critical for Tau and PRC1 binding to mammalian B55 *in vivo*. Further studies, including substrates regulated by other PP2A^{Cdc55}-counteracted kinases, could help elucidate how this specific phosphatase recognizes and interacts with its substrates.

This work attempts to bring new insight into the mitotic exit regulation picture, with a special focus on PP2A^{Cdc55} functions in this critical phase of cell division. A profound understanding of mitotic exit regulation could set the stage for new therapeutic strategies, since failure to progress normally through mitotic exit can induce cell death and could be exploited to kill hyper-proliferating cancer cells. The study of phosphatase holoenzymes, and especially, the regulatory phosphatase subunits such as Cdc55, provides valuable information for the development of new pharmacological inhibitors or modulators that selectively target specific phosphatase complexes.

Potential implications

Dysregulation of PP2A phosphatases have been found in many solid cancers and leukemias. PP2A-B55, and its highly-conserved homolog in budding yeast, PP2A^{Cdc55},

regulate the cell cycle and are required for efficient mitotic exit. Budding yeast is thus a powerful model to gain insight into mitotic exit regulation, specifically, to the activities of PP2A phosphatase holoenzymes, which could promote the design of new therapeutic strategies, since failure to progress normally through mitotic exit may be exploited to kill hyper-proliferating cancer cells. Here, we used phosphoproteomics of Cdc55 deficient cells to uncover new $PP2A^{Cdc55}$ substrates and functions in mitosis. We also reveal new kinases potentially counteracted and regulated by $PP2A^{Cdc55}$ phosphatase. In particular, Pkc1 and Cla4 kinases were discovered as significant $PP2A^{Cdc55}$ regulation nodes. Finally, we attempted to gain insight into Cdc55-susbtrate interaction using docking models of Cdc55 and Mob1 substrate, which suggest a specific interface for substrate interaction.

Methods

Yeast strains, plasmids and cell cycle synchronization procedures

All yeast strains used in this study were derivatives of W303. Epitope tagging of endogenous genes was performed by gene targeting using polymerase chain reaction (PCR) products. Endogenous *CDC55* was N-terminal-tagged as previously described (39). Metaphase arrest by Cdc20 depletion was also performed as previously described (76).

Stable Isotope Labelling of Yeast Cells and Preparation of Yeast Protein Extracts for Phosphoproteomic Analysis

For each biological replicate, yeast cells were labelled with stable isotopes and protein extracts prepared as previously described (77). In brief, cells were grown in minimum media containing either 100 mg/L arginine and 100 mg/L lysine or 100 mg/L $^{13}C_6$-arginine

and 100 mg/L $^{13}C_6$-lysine (Cambridge Isotope Laboratories Inc.). Y859 (*MAT a lys2Δ::TRP1*, *arg4Δ::HIS3 MET-Cdc20::LEU2*) and Y858 (as Y859 but *cdc55Δ*) cells were grown in free-methionine minimum media containing $^{13}C_6$-lysine and -arginine (heavy) or unmodified arginine and lysine (light), respectively. Both strains were synchronized at the metaphase-to-anaphase transition by adding methionine to the media. Protein extracts were prepared by mechanical lysis using glass beads in presence of protein inhibitors (Complete EDTA-free, Roche) and 2X phosphatase inhibitors PhosStop (Roche). Cell lysates were mixed 1:1 and digestion with trypsin was performed. Approximately 400 μg of the mixed heavy/light protein sample were processed for in-solution digestion as previously described (78). Proteins were reduced with 5 mM DTT for 30 min at 37ºC and alkylated with 10 mM iodoacetamide for 30 min at 30ºC. Samples were diluted five times with 25 mM ammonium bicarbonate, trypsin (Promega, ratio enzyme:protein 1:10) was added and incubated overnight at 37ºC. Digestion was stopped by addition of formic acid.

Phosphopeptide enrichment

Three strategies were used for phosphopeptide enrichment. In the first approach, phosphopeptide enrichment by sequential elution from IMAC (SIMAC) was done as previously described (77). Peptides samples were added to an immobilized metal affinity chromatography suspension (Phos-Select, Sigma) and were incubated for 1h at room temperature. The flow-through was collected, and the immobilized metal affinity chromatography resin was washed once with 50 μl 50% ACN and 0.1% TFA. The wash fraction was pooled with the flow-through. Acid elution was then carried out by adding 50 μl 30% ACN and 1% TFA and incubating for 5 min at room temperature. After this step,

alkaline elution was done with 50 µl 0.5% NH_4OH pH 10.5, followed by 30 min incubation at room temperature. For further enrichment of phosphopeptides, the flow-through fraction and the acid eluate were incubated with TiO_2 beads (GL Sciences, Tokyo, Japan) and incubated with shaking for 1 h at 30 °C. The TiO_2 beads were washed twice with 80% ACN and 1% TFA and once with water. Bound peptides were eluted from the beads with 0.5% NH_4OH pH 10.5 for 30 min at 30 °C. Eluted peptides were dried via centrifugal evaporation, resuspended with 1 µl formic acid and 15 µl water and analyzed using nano-LC-MS/MS on an LTQ-Orbitrap (Themo Scientific) mass spectrometer.

In the second strategy, phosphopeptide enrichment was done using TiO_2 chromatography following the product specifications (TiO_2 Mag Sepharose, GE Healthcare). An aliquot of 100 µg was separated to be further processed and analyzed without phosphopeptide enrichment. All samples (enriched and non-enriched for phosphopeptide) were dried via centrifugal evaporation and subjected to fractionation with a high pH reversed phase peptide fractionation kit (Pierce). The peptides were eluted in 9 fractions of increasing acetonitrile (ACN) concentration of 5% ACN to75% ACN. The 9 eluted fractions were dried via centrifugal evaporation, resuspended in 1% FA and analyzed in a nanoAcquity liquid chromatographer (Waters) coupled to an LTQ-Orbitrap Velos (Thermo Scientific) mass spectrometer.

In the third approach, a combination of enrichment and fractionation methods was used (The “TiSH” method: TiO_2-SIMAC-HILIC) as previously described(79). Briefly, peptide digest was first pre-enriched in phosphopeptides using TiO_2 chromatography (80) (5 µm, GL Sciences Inc, Japan) followed by SIMAC purification (81). The mono-phosphorylated peptide fraction from the SIMAC enrichment was further subjected to a second TiO_2

purification. The mono-phosphorylated fraction was then pre-fractionated by HILIC chromatography (Hydrophilic Interaction Liquid Chromatography, Column TSK Gel Amide 80 15 cm 0,3mm ID) using a 40 min gradient from 90% B buffer (95% acetonitrile, 0.1% TFA) to 60 % B buffer. Twenty-five fractions were collected, which were pooled into a final five fractions that were then analyzed by reverse phase LC-MS/MS. The multi-phosphorylated fraction from SIMAC was directly analyzed by LC-MS/MS after desalting and concentration using a Poros Oligo R3 (ABSciex) Reversed phase (RP) micro-column.

LC-MS/MS Analysis

For the first approach, the peptides were analyzed using nano-LC-Ms/MS on an LTQ-Orbitrap Velos (Thermo Scientific) mass spectrometer. Peptides were separated on a BioBasic C-18 PicoFrit column (75 µm Øi, 10 cm, New Objective, Woburn, MA) at a flow rate of 200 nL/min. Water and ACN, both containing 0.1% formic acid, were used as solvents A and B, respectively. Peptides were trapped and desalted in the trap column for 5 min. The gradient was started and kept at 10% B for 5 min, ramped to 60% B over 60 min or 120 min, depending on the sample complexity, and kept at 90% B for another 5 min. Peptides (m/z 400-1400) were analyzed on the LTQ-Orbitrap velos in full Scan MS mode with a resolution of 60,000 FWHM at 400m/z; up to the 7 most abundant peptides were selected from each MS scan and then fragmented using collision induced dissociation in a linear ion trap using helium as collision gas at 7500 FWHM and 30 sec exclusion time. Generated .raw data files were collected with Thermo Xcalibur v.2.2.

For the second approach, the peptides (enriched and non-enriched) were resuspended in 1% FA and were injected for chromatographic separation. Peptides were trapped on a Symmetry C18^{TM} trap column (Waters), and were separated using a C18 reverse phase

capillary column (75 µm Øi, 25 cm, nano Acquity, 1.7µm BEH column; Waters). The gradient used for the elution of the peptides was 1 to 35 % B in 90 min, followed by a gradient from 35% to 85% in 10 min (A: 0.1% FA; B: 100% ACN, 0.1%FA), with a 250 nL/min flow rate. Eluted peptides were subjected to electrospray ionization in an emitter needle (PicoTip^{TM}, New Objective) with an applied voltage of 2000V. Peptide masses (*m/z* 300-1700) were analyzed in data dependent mode where a full Scan MS was acquired in the Orbitrap with a resolution of 60,000 FWHM at 400m/z. Up to the 10 most abundant peptides (minimum intensity of 500 counts) were selected from each MS scan and then fragmented using CID (Collision induced Dissociation) in the linear ion trap using helium as collision gas. Multistage activation was enabled to favor the detection of phosphopeptides. The scan time settings were: Full MS: 250 ms and MSn: 120 ms. Generated .raw data files were collected with Thermo Xcalibur v.2.2.

For the third strategy, the peptides were resuspended in 0.1 % TFA and analyzed using an Easy-nanoLC (Thermo Fisher Scientific, Proxeon, Denmark) coupled to an LTQ-Orbitrap Fusion Tribride mass spectrometer (Thermo Fisher Scientific). Peptides were loaded onto a pre-column of 2cm Reprosil –Pur C18 AQ 5 µm RP material (Dr. Maishc, Ammerbuch-Entrigen, Germany) using the EASY-LC system and eluted directly onto a 20 cm long fused silica capillary column (75 µm ID) packed with Reprosil- Pur C18 AQ 3 µm RP material. The peptides were separated using a gradient from 0-34% B (A buffer: 0.1 % formic acid (FA); B buffer: 90% ACN/0.1% FA) at a flow rate of 250 nL/min over 30-60 min depending on the UV trace of the HILIC fractions. The peptides (m/z 400-1400) were analyzed in full MS mode using a resolution of 120.000 FWHM at 200 m/z and the peptides were selected and fragmented using helium as collision gas and the fragment ions were recorded in the LTQ with low resolution (rapid scan rate). A maximum of 3 sec were

allowed between each MS and for MSMS the ion filling time was set to 40 ms and an AGC target value of 2E4 ions. Raw data was viewed in Xcalibur v2.0.7.

Data Analysis for Peptide Identification and Quantification

Peptide identification was performed using Proteome Discoverer v1.4.1.14 (Thermo Scientific) and search against Swiss Prot /Uniprot *Saccharomyces cerevisiae* database (v. January 2016) with SequestHT search engine. Both a target and a decoy database were searched to obtain a false discovery rate (FDR). To improve the sensitivity of the database search, Percolator (semi-supervised learning machine) was used to discriminate correct from incorrect peptide spectrum matches. The PhosphoRS node was used to provide a confidence measure for the localization of phosphorylation in the peptide sequences identified with this modification.

Database search parameters were: precursor mass tolerance 10 ppm, fragment mass tolerance 0.6 Da, cysteine carbamydomethylation as fixed modification and 2 missed cleavage for trypsin. Variable modifications considered were phosphorylation on S/T/Y and K/R label:$^{13}C_6$ and oxidation (M).

Only peptides with high confidence Percolator q of 0,01 (FDR<1%) were considered for further analyses.

Peptide quantification from SILAC labels was performed with Proteome Discoverer v1.4. The log2-ratio value associated with each peptide was calculated as a weighted average of the scans used to quantify the peptide, as described elsewhere (58, 59) and the data were normalized based on the median. Only quantified peptides detected as statistically significant (high confidence FDR< 0.01) were selected. The processing of the data was performed in R (v.3.3.1) with the help of the ‘rvest’, ‘Vennerable’ and ‘Venneuler’

packages. Briefly, the H/L ratios from the samples (TiSH, SIMAC and TiO_2) were averaged for every phosphopeptide. The resulting list was filtered to keep only the phosphopeptides of interest. That is, peptides with a coefficient of variation (CV) between samples below 40%, peptides without CV (peptides only appearing in one sample) and peptides with a CV above 40% which show a H/L ratio in all the samples below 0.75. Statistical significance was assessed at 5% (two-tailed Student's t-test; $p<0.05$). The mass spectrometry proteomics data have been deposited to the ProteomeXchange Consortium (82) via the PRIDE (83) partner repository with the dataset identifier PXD007613 (**Username:** reviewer50711@ebi.ac.uk, **Password:** Tv9GFPI2).

Phosphorylation motif analysis

The Phosphorylation Motifs Enrichment Analysis (PMEA) was performed with the motif-X web tool (http://motif-x.med.harvard.edu/) (84). Before the analysis, the phosphosites were aligned so that the phosphosite is centered. All the peptides identified in our 3 SILAC approaches were used to search for enriched motifs against the SGD yeast proteome database as a background.

TAP purification

Protein extracts were prepared by mechanical lysis using glass beads in presence of protein inhibitors (Complete EDTA-free, Roche) and 2X phosphatase inhibitors PhosStop (Roche). TAP (*T*andem *A*ffinity *P*urification of Protein A and CBP (calmodulin binding protein) epitopes), fusion proteins and associated proteins were recovered from cell extracts by affinity chromatography using an IgG-sepharose matrix. After washing, the Tobacco Etch

Virus (AcTEV, Life technologies) protease was added to release the bound material. The eluate was incubated with calmodulin-coated beads in the presence of calcium. This second affinity step was required to remove not only the AcTEV protease but also traces of contaminants remaining after first affinity purification. After washing, the bound material was released with ethylene glycol tetra acetic acid (EGTA). The calmodulin eluates from the TAP-purified complexes were precipitated with trichloroacetic acid (TCA) and directly subjected to LC-MS/MS. Pellets were dissolved with 20 μL of 50 mM ammonium bicarbonate (ABC). Cysteine residues were reduced by 2 mM DTT (DL-Dithiothreitol) in 50 mM ABC at 60º for 20 min. Sulfhydryl groups were alkylated with 5 mM iodoacetamide (IAM) in 50 mM ABC in the dark at RT for 30 min. IAM excess was neutralized with 10 mM DTT in 50 mM ABC 30 min at RT. 5 μL of each sample were loaded onto a trap column (nanoLC column, 3 μ C18-CL, 75 μmx15cm; Eksigen) and desalted with 0.1% TFA at 2 μL/min during 10 min. The peptides were then loaded onto an analytical column (LC Column, 3 μ C18-CL, 75 μmx15cm; Eksigen) equilibrated in 5 % acetonitrile 0.1% FA (formic acid). Elution was carried out with a linear gradient of 5-35% B in A for 120 min (A: 0.1% FA; B: AN 0.1% FA) at a flow rate of 300 nL/min. Peptides were analyzed in a mass spectrometer nanoESI qQTOF (5600 TripleTOF, ABSCIEX). The tripleTOF was operated in information-dependent acquisition mode, in which a 0.25-s TOF MS scan 350-1250 m/Z, was performed, followed by 0.05-s product ion scans from 100-1500 m/z on the 50 most intense 2-5 charged ions. Protein identification was performed using ProteinPilot v4.0.8085 (ABSciex) or Mascot v2.3 (Matrix Science) search engines. Protein Pilot default parameters were used to generate peak list directly from 5600 TripleTOF wiff files. The Paragon algorithm of ProteinPilot was used to search Expasy

protein database (1072964 sequences). The proteomic analysis was carried out in the SCSIE_university of Valencia Proteomics Unit, a member of ISCIII ProteoRed Proteomics Platform. Peptides identified in two TAP-Cdc55 biological replicates and the untagged control had been deposited to the ProteomeXchange Consortium with the dataset identifier PXD007613.

HA Purification

Protein extracts were prepared by mechanical lysis using glass beads in presence of protein inhibitors (Complete EDTA-free, Roche) and 2X phosphatase inhibitors PhosStop (Roche). HA-Cdc55 fusion proteins and associated proteins were recovered from cell extracts by HA-agarose beads (Sigma). The eluates were precipitated with trichloroacetic acid (TCA) and proteins were separated in a protein gel. After trypsin digestion, peptide was desalted by Strata X C18 column (Phenomenex) and vacuum-dried. A total of 1μg dried peptide was reconstituted in a solution containing 65% ACN, 2% TFA and was saturated with glutamic acid (20 mg/ml, pH 2.0-2.5). Then the peptide solution was added to TiO_2 (GL Science, Saitama) and was incubated for 20 min. The peptides were eluted once with 1.1% NH_4OH solution in 50% ACN and once with 3% NH_4OH solution in 50% ACN (diluted from 25% NH4OH solution). Two elute fractions were combined and vacuum-dried. Then, phosphopeptides were subjected to nanoelectrospray ionization followed by tandem mass spectrometry (MS/MS) on a Q-Exactive mass spectrometer (ThermoFisher Scientific). Peptide and protein modification were obtained using Mascot software. TAP purifications experiments were performed using BGI proteomic services and BGI bioinformatics department.

Western Blot validation of cell cycle-dependent phosphorylated substrates

Cell synchronization by Cdc20 depletion and entry into synchronous anaphase by Cdc20 re-introduction were also performed as previously described (42). Protein extracts for western blots were obtained by TCA protein extraction. Gels of 8-10 % were used for electrophoresis. Antibodies used for protein staining were α-HA clone 12CA5 (Roche) and α-Pk clone SV5-Pk1 (Serotec).

Interaction maps and Gene ontology

The networks were created with the STRING database (http://string-db.org/) (85). Only high-confidence interactions from experiments or databases were extracted and binary interactions were also discarded.

Classification into functional clusters and gene ontology was performed with the DAVID bioinformatics tools (https://david.ncifcrf.gov/) (86). Only clusters with an Enrichment Score higher than 1.5 and GO terms with a $p<0.001$ were considered.

Structure Prediction of Cdc55

A structural model of full-length yeast Cdc55 (Uniprot AC: 2ABA_YEAST) was built by homology modeling. HHpred (87) identified the regulatory B55 subunit of the heterotrimeric human protein phosphatase PP2A (Uniprot AC: 2ABA_HUMAN; PDB: 3dw8_B) as a suitable template and provided a pairwise alignment. We then used the loop model protocol implemented in MODELLER 9v18 (88) to build 50 models of CDC55, which were assessed and ranked with the DOPE statistical potential (89).

Sampling the binding interface of the CDC55/Mob1 complex.

Models of the interaction between CDC55 and Mob1 were calculated using the data-driven docking software HADDOCK (version 2.2) (66). As initial structures, we used the Cdc55 homology model with the lowest (best) DOPE score and the available crystal structure of Mob1 (PDB: 2HJN_A). We restricted the search on Cdc55 to solvent accessible residues within a 10 Å radius of the Tau binding region identified by NMR and mutagenesis experiments on the homologous B55 (3). All residues are strictly conserved between the two proteins: E24, K45, F75, D76, Y77, L78, K79, S80, L81, E84, E85, K86, Y185, H186 and D204. For Mob1, we defined the entire surface of the protein as a possible interaction site. A residue was defined as solvent accessible if its main-chain or side-chain atoms had a relative solvent accessibility equal to or greater than 15% as calculated by FREESASA (90) and the NACCESS scale.

We calculated 100.000 models using the data-driven rigid-body docking protocol in HADDOCK and kept the best 10.000 (top 10%) ranked by HADDOCK score for further analysis. We then superimposed these models on the heterotrimeric PP2A structure and calculated the distance between residues P81 in Mob1 (proxy for the phosphosite S80, not resolved in the crystal) and H118 (proton donor) in the catalytic subunit of PP2A. Using a threshold of 10 Å as filtered, we obtained a list of 294 models, which we then grouped in 12 representative clusters using a fast contact-based interface similarity algorithm (91). We also used these 294 models to calculate propensities for each individual residue to be part of the Cdc55/Mob1 interface. A residue was defined as part of the interface if any of its atoms was within 5 Å of any atom of the partner protein.

Acknowledgements

We wish to thank Brendan Kelly, Priscilla Aquino, and all the members of our laboratory for discussion and their critical reading of the manuscript.

Funding information:

Work in our laboratory is supported by the Spanish Ministry of Science and Innovation (BFU2011-27568), Spanish Ministry of Economy and Competitively (BFU2013-43132-P and BFU2016-77975-R AEI/FEDER, UE cofounded by FEDER funds/European Regional Development Fund- a way to build Europe). MRL was supported by the Lundbeck foundation (Junior Group Leader Fellowship). This work was supported by a generous grant from the VILLUM Foundation to the VILLUM Centre for Bioanalytical Sciences at the University of Southern Denmark. SBB is a recipient of ISCIII grant 13FIS037. IDIBELL Proteomics Unit belongs to ProteoRed, PRB2-ISCIII, and is supported by grant PT13/0001/0033.

Author contribution: BB, SJ, JV, IC and EQ performed the experiments. CG, MLH, SBB, CDLT and MRL performed the SILAC experiments. JJBS, BB and EQ performed and discussed the bioinformatics analysis. BB and JR did the *in silico* docking experiments. BB and EQ design the experiments, interpreted the data and wrote the manuscript. All authors read and discussed the manuscript.

Conflict of interest

The authors declare that they have no conflicts of interest.

References

1. Mustelin T. 2007. A brief introduction to the protein phosphatase families. Methods Mol Biol 365:9–22.
2. Stark MJ. 1996. Yeast protein serine/threonine phosphatases: multiple roles and diverse regulation. Yeast 12:1647–1675.
3. Shi Y. 2009. Serine/Threonine Phosphatases: Mechanism through Structure. Cell 139:468–484.
4. Kitajima TS, Sakuno T, Ishiguro K, Iemura S, Natsume T, Kawashima S a, Watanabe Y. 2006. Shugoshin collaborates with protein phosphatase 2A to protect cohesin. Nature 441:46–52.
5. Riedel CG, Katis VL, Katou Y, Mori S, Itoh T, Helmhart W, GÃ¡lovÃ¡ M, Petronczki M, Gregan J, Cetin B, Mudrak I, Ogris E, Mechtler K, Pelletier L, Buchholz F, Shirahige K, Nasmyth K. 2006. Protein phosphatase 2A protects centromeric sister chromatid cohesion during meiosis I. Nature 441:53–61.
6. Tang Z, Shu H, Qi W, Mahmood NA, Mumby MC, Yu H. 2006. PP2A Is Required for Centromeric Localization of Sgo1 and Proper Chromosome Segregation. Dev Cell 10:575–585.
7. Gregan J, Spirek M, Rumpf C. 2008. Solving the shugoshin puzzle. Trends Genet.
8. Queralt E, Lehane C, Novak B, Uhlmann F. 2006. Downregulation of PP2ACdc55 Phosphatase by Separase Initiates Mitotic Exit in Budding Yeast. Cell 125:719–732.
9. Drewes G, Mandelkow EM, Baumann K, Goris J, Merlevede W, Mandelkow E. 1993. Dephosphorylation of tau protein and Alzheimer paired helical filaments by calcineurin and phosphatase-2A. FEBS Lett 336:425–32.

10. Gong CX, Grundke-Iqbal I, Iqbal K. 1994. Dephosphorylation of Alzheimer's disease abnormally phosphorylated tau by protein phosphatase-2A. Neuroscience 61:765–772.

11. Xu Y, Chen Y, Zhang P, Jeffrey PD, Shi Y. 2008. Structure of a Protein Phosphatase 2A Holoenzyme: Insights into B55-Mediated Tau Dephosphorylation. Mol Cell 31:873–885.

12. Mo S-T, Chiang S-J, Lai T-Y, Cheng Y-L, Chung C-E, Kuo SCH, Reece KM, Chen Y-C, Chang N-S, Wadzinski BE, Chiang C-W. 2014. Visualization of Subunit Interactions and Ternary Complexes of Protein Phosphatase 2A in Mammalian Cells. PLoS One 9:e116074.

13. Götz J, Probst A, Ehler E, Hemmings B, Kues W. 1998. Delayed embryonic lethality in mice lacking protein phosphatase 2A catalytic subunit Calpha. Proc Natl Acad Sci U S A 95:12370–5.

14. Kong M, Fox CJ, Mu J, Solt L, Xu A, Cinalli RM, Birnbaum MJ, Lindsten T, Thompson CB. 2004. The PP2A-Associated Protein 4 Is an Essential Inhibitor of Apoptosis. Science (80-) 306:695–698.

15. Li X, Scuderi A, Letsou A, Virshup DM. 2002. B56-Associated Protein Phosphatase 2A Is Required For Survival and Protects from Apoptosis in Drosophila melanogaster. Mol Cell Biol 22:3674–3684.

16. Silverstein AM, Barrow C a, Davis AJ, Mumby MC. 2002. Actions of PP2A on the MAP kinase pathway and apoptosis are mediated by distinct regulatory subunits. Proc Natl Acad Sci U S A 99:4221–4226.

17. Strack S, Cribbs JT, Gomez L. 2004. Critical role for protein phosphatase 2A heterotrimers in mammalian cell survival. J Biol Chem 279:47732–47739.

18. Sneddon AA, Cohen PT, Stark MJ. 1990. Saccharomyces cerevisiae protein phosphatase 2A performs an essential cellular function and is encoded by two genes. EMBO J 9:4339–46.

19. Ronne H, Carlberg M, Hu GZ, Nehlin JO. 1991. Protein phosphatase 2A in Saccharomyces cerevisiae: effects on cell growth and bud morphogenesis. Mol Cell Biol 11:4876–4884.

20. Wlodarchak N, Xing Y. 2016. PP2A as a master regulator of the cell cycle. Crit Rev Biochem Mol Biol 51:162–184.

21. Juanes MA, Khoueiry R, Kupka T, Castro A, Mudrak I, Ogris E, Lorca T, Piatti S. 2013. Budding Yeast Greatwall and Endosulfines Control Activity and Spatial Regulation of PP2ACdc55 for Timely Mitotic Progression. PLoS Genet 9.

22. Yamamoto TM, Blake-Hodek K, Williams BC, Lewellyn AL, Goldberg ML, Maller JL. 2011. Regulation of Greatwall kinase during Xenopus oocyte maturation. Mol Biol Cell 22:2157–64.

23. Harvey SL, Charlet A, Haas W, Gygi SP, Kellogg DR. 2005. Cdk1-dependent regulation of the mitotic inhibitor Wee1. Cell 122:407–420.

24. Harvey SL, Enciso G, Dephoure N, Gygi SP, Gunawardena J, Kellogg DR. 2011. A phosphatase threshold sets the level of Cdk1 activity in early mitosis in budding yeast. Mol Biol Cell 22:3595–3608.

25. Minshull J, Straight A, Rudner AD, Dernburg AF, Belmont A, Murray AW. 1996. Protein phosphatase 2A regulates MPF activity and sister chromatid cohesion in budding yeast. Curr Biol 6:1609–1620.

26. Wang Y, Burke DJ. 1997. Cdc55p, the B-type regulatory subunit of protein phosphatase 2A, has multiple functions in mitosis and is required for the

kinetochore/spindle checkpoint in Saccharomyces cerevisiae. Mol Cell Biol 17:620–626.

27. Yang H, Jiang W, Gentry M, Hallberg RL. 2000. Loss of a Protein Phosphatase 2A Regulatory Subunit (Cdc55p) Elicits Improper Regulation of Swe1p Degradation. Mol Cell Biol 20:8143–8156.

28. Lucena R, Alcaide-Gavilán M, Anastasia SD, Kellogg DR. 2017. Wee1 and Cdc25 are controlled by conserved PP2A-dependent mechanisms in fission yeast. Cell Cycle 16:428–435.

29. Pal G, Paraz MT, Kellogg DR. 2008. Regulation of Mih1/Cdc25 by protein phosphatase 2A and casein kinase 1. J Cell Biol2008/03/05. 180:931–945.

30. Yasutis K, Vignali M, Ryder M, Tameire F, Dighe SA, Fields S, Kozminski KG. 2010. Zds2p regulates Swe1p-dependent polarized cell growth in Saccharomyces cerevisiae via a novel Cdc55p interaction domain. Mol Biol Cell2010/10/29. 21:4373–4386.

31. Wicky S, Tjandra H, Schieltz D, Yates 3rd J, Kellogg DR. The Zds proteins control entry into mitosis and target protein phosphatase 2A to the Cdc25 phosphatase. Mol Biol Cell2010/12/02. 22:20–32.

32. Anastasia SD, Nguyen DL, Thai V, Meloy M, MacDonough T, Kellogg DR. 2012. A link between mitotic entry and membrane growth suggests a novel model for cell size control. J Cell Biol 197:89–104.

33. Jonasson EM, Rossio V, Hatakeyama R, Abe M, Ohya Y, Yoshida S. 2016. Zds1/Zds2–PP2A(Cdc55) complex specifies signaling output from Rho1 GTPase. J Cell Biol 212:51–61.

34. Thai V, Dephoure N, Weiss A, Ferguson J, Leitao R, Gygi SP, Kellogg DR. 2017.

Protein kinase C controls binding of Igo/ENSA proteins to protein phosphatase 2A in budding yeast. J Biol Chem .

35. Vázquez-Novelle MD, Esteban V, Bueno A, Sacristán MP. 2005. Functional homology among human and fission yeast Cdc14 phosphatases. J Biol Chem 280:29144–29150.

36. Berdougo E, Nachury M V., Jackson PK, Jallepalli P V. 2008. The nucleolar phosphatase Cdc14B is dispensable for chromosome segregation and mitotic exit in human cells. Cell Cycle 7:1184–1190.

37. Wu JQ, Guo JY, Tang W, Yang C-S, Freel CD, Chen C, Nairn AC, Kornbluth S. 2009. PP1-mediated dephosphorylation of phosphoproteins at mitotic exit is controlled by inhibitor-1 and PP1 phosphorylation. Nat Cell Biol.

38. Schmitz MHA, Held M, Janssens V, Hutchins JRA, Hudecz O, Ivanova E, Goris J, Trinkle-Mulcahy L, Lamond AI, Poser I, Hyman AA, Mechtler K, Peters J-M, Gerlich DW. 2010. Live-cell imaging RNAi screen identifies PP2A-B55alpha and importin-beta1 as key mitotic exit regulators in human cells. Nat Cell Biol 12:886–93.

39. Queralt E, Lehane C, Novak B, Uhlmann F. 2006. Downregulation of PP2ACdc55 Phosphatase by Separase Initiates Mitotic Exit in Budding Yeast. Cell 125:719–732.

40. Queralt E, Uhlmann F. 2008. Separase cooperates with Zds1 and Zds2 to activate Cdc14 phosphatase in early anaphase. J Cell Biol 182:873–883.

41. Calabria I, Baro B, Rodriguez-Rodriguez J-A, Russinol N, Queralt E. 2012. Zds1 regulates PP2ACdc55 activity and Cdc14 activation during mitotic exit through its Zds_C motif. J Cell Sci.

42. Baro B, Rodriguez-Rodriguez JA, Calabria I, Hernáez ML, Gil C, Queralt E. 2013.

Dual Regulation of the Mitotic Exit Network (MEN) by PP2A-Cdc55 Phosphatase. PLoS Genet 9.

43. Yaakov G, Thorn K, Morgan DO. 2012. Separase Biosensor Reveals that Cohesin Cleavage Timing Depends on Phosphatase PP2ACdc55 Regulation. Dev Cell 23:124–136.

44. Vernieri C, Chiroli E, Francia V, Gross F, Ciliberto A. 2013. Adaptation to the spindle checkpoint is regulated by the interplay between Cdc28/Clbs and PP2ACdc55. J Cell Biol 202:765–778.

45. Lianga N, Williams EC, Kennedy EK, Doré C, Pilon S, Girard SL, Deneault JS, Rudner AD. 2013. A wee1 checkpoint inhibits anaphase onset. J Cell Biol 201:843–862.

46. Boronat S, Campbell JL. 2007. Mitotic Cdc6 Stabilizes Anaphase-Promoting Complex Substrates by a Partially Cdc28-Independent Mechanism, and This Stabilization Is Suppressed by Deletion of Cdc55. Mol Cell Biol 27:1158–1171.

47. Mui MZ, Roopchand DE, Gentry MS, Hallberg RL, Vogel J, Branton PE. 2010. Adenovirus protein E4orf4 induces premature APCCdc20 activation in Saccharomyces cerevisiae by a protein phosphatase 2A-dependent mechanism. J Virol 84:4798–809.

48. Holt LJ, Tuch BB, Villén J, Johnson AD, Gygi SP, Morgan DO. 2009. Global analysis of Cdk1 substrate phosphorylation sites provides insights into evolution. Science 325:1682–6.

49. Ubersax J a, Woodbury EL, Quang PN, Paraz M, Blethrow JD, Shah K, Shokat KM, Morgan DO. 2003. Targets of the cyclin-dependent kinase Cdk1. Nature 425:859–864.

50. Kao L, Wang Y-T, Chen Y-C, Tseng S-F, Jhang J-C, Chen Y-J, Teng S-C. 2014. Global Analysis of Cdc14 Dephosphorylation Sites Reveals Essential Regulatory Role in Mitosis and Cytokinesis. Mol Cell Proteomics 13:594–605.

51. Bloom J, Cristea IM, Procko AL, Lubkov V, Chait BT, Snyder M, Cross FR. 2011. Global analysis of Cdc14 phosphatase reveals diverse roles in mitotic processes. J Biol Chem 286:5434–5445.

52. Janssens V, Longin S, Goris J. 2008. PP2A holoenzyme assembly: in cauda venenum (the sting is in the tail)Trends Biochem Sci.

53. Godfrey M, Touati SA, Kataria M, Jones A, Snijders AP, Uhlmann F. 2017. PP2A(Cdc55) Phosphatase Imposes Ordered Cell-Cycle Phosphorylation by Opposing Threonine Phosphorylation. Mol Cell 65:393–402.e3.

54. Cundell MJ, Hutter LH, Bastos RN, Poser E, Holder J, Mohammed S, Novak B, Barr FA. 2016. A PP2A-B55 recognition signal controls substrate dephosphorylation kinetics during mitotic exit. J Cell Biol 214:539–554.

55. Bontron S, Jaquenoud M, Vaga S, Talarek N, Bodenmiller B, Aebersold R, De Virgilio C. 2013. Yeast Endosulfines Control Entry into Quiescence and Chronological Life Span by Inhibiting Protein Phosphatase 2A. Cell Rep 3:16–22.

56. Talarek N, Gueydon E, Schwob E. 2017. Homeostatic control of start through negative feedback between Cln3-Cdk1 and Rim15/greatwall kinase in budding yeast. Elife 6.

57. Schwartz D, Gygi SP. 2005. An iterative statistical approach to the identification of protein phosphorylation motifs from large-scale data sets. NatBiotechnol 23:1391–1398.

58. Mok J, Kim PM, Lam HYK, Piccirillo S, Zhou X, Jeschke GR, Sheridan DL, Parker

S a, Desai V, Jwa M, Cameroni E, Niu H, Good M, Remenyi A, Ma J-LN, Sheu Y-J, Sassi HE, Sopko R, Chan CSM, De Virgilio C, Hollingsworth NM, Lim W a, Stern DF, Stillman B, Andrews BJ, Gerstein MB, Snyder M, Turk BE. 2010. Deciphering protein kinase specificity through large-scale analysis of yeast phosphorylation site motifs. Sci Signal 3:ra12.

59. Paulson JL, Sullivan M, Lowery DM, Cohen MS, Zhang C, Randle DH, Taunton J, Yaffe MB, Morgan DO, Shokat KM. 2007. A Coupled Chemical Genetic and Bioinformatic Approach to Polo-like Kinase Pathway Exploration. Chem Biol 14:1261–1272.

60. Juanes MA, Piatti S. 2016. The final cut: cell polarity meets cytokinesis at the bud neck in S. cerevisiae. Cell Mol Life Sci.

61. McCourt P, Gallo-Ebert C, Gonghong Y, Jiang Y, Nickels JT. 2013. PP2ACdc55 regulates G1 cyclin stability. Cell Cycle 12:1201–1210.

62. Moreno-Torres M, Jaquenoud M, De Virgilio C. 2015. TORC1 controls G1-S cell cycle transition in yeast via Mpk1 and the greatwall kinase pathway. Nat Commun 6:8256.

63. Wang Y, Burke DJ. 1997. Cdc55p, the B-type regulatory subunit of protein phosphatase 2A, has multiple functions in mitosis and is required for the kinetochore/spindle checkpoint in Saccharomyces cerevisiae. Mol Cell Biol 17:620–626.

64. Riedel CG, Katis VL, Katou Y, Mori S, Itoh T, Helmhart W, Gálová M, Petronczki M, Gregan J, Cetin B, Mudrak I, Ogris E, Mechtler K, Pelletier L, Buchholz F, Shirahige K, Nasmyth K. 2006. Protein phosphatase 2A protects centromeric sister chromatid cohesion during meiosis I. Nature 441:53–61.

65. Zapata J, Dephoure N, Macdonough T, Yu Y, Parnell EJ, Mooring M, Gygi SP, Stillman DJ, Kellogg DR. 2014. PP2ARts1 is a master regulator of pathways that control cell size. J Cell Biol 204:359–76.

66. Van Zundert GCP, Rodrigues JPGLM, Trellet M, Schmitz C, Kastritis PL, Karaca E, Melquiond ASJ, Van Dijk M, De Vries SJ, Bonvin AMJJ. 2016. The HADDOCK2.2 Web Server: User-Friendly Integrative Modeling of Biomolecular Complexes. J Mol Biol 428:720–725.

67. Xu Y, Xing Y, Chen Y, Chao Y, Lin Z, Fan E, Yu JW, Strack S, Jeffrey PD, Shi Y. 2006. Structure of the Protein Phosphatase 2A Holoenzyme. Cell 127:1239–1251.

68. Robinson LC, Menold MM, Garrett S, Culbertson MR. 1993. Casein kinase I-like protein kinases encoded by YCK1 and YCK2 are required for yeast morphogenesis. Mol Cell Biol 13:2870–2881.

69. Robinson LC, Bradley C, Bryan JD, Jerome A, Kweon Y, Panek HR. 1999. The Yck2 yeast casein kinase 1 isoform shows cell cycle-specific localization to sites of polarized growth and is required for proper septin organization. Mol Biol Cell 10:1077–1092.

70. Cvrckova F, De Virgilio C, Manser E, Pringle JR, Nasmyth K. 1995. Ste20-like protein kinases are required for normal localization of cell growth and for cytokinesis in budding yeast. Genes Dev 9:1817–1830.

71. Kadota J, Yamamoto T, Yoshiuchi S, Bi E, Tanaka K. 2004. Septin Ring Assembly Requires Concerted Action of Polarisome Components, a PAK Kinase Cla4p, and the Actin Cytoskeleton in Saccharomyces cerevisiae. Mol Biol Cell 15:5329–5345.

72. Versele M, Thorner J. 2004. Septin collar formation in budding yeast requires GTP binding and direct phosphorylation by the PAK, Cla4. J Cell Biol 164:701–715.

73. Traven A, Beilharz TH, Lo TL, Lueder F, Preiss T, Heierhorst J. 2009. The Ccr4-Pop2-NOT mRNA Deadenylase Contributes to Septin Organization in Saccharomyces cerevisiae. Genetics 182:955–966.

74. Boyce KJ, Andrianopoulos A. 2011. Ste20-related kinases: Effectors of signaling and morphogenesis in fungi. Trends Microbiol.

75. Huang Z-X, Zhao P, Zeng G-S, Wang Y-M, Sudbery I, Wang Y. 2014. Phosphoregulation of Nap1 Plays a Role in Septin Ring Dynamics and Morphogenesis in Candida albicans. MBio 5:e00915-13.

76. Uhlmann F, Lottspeich F, Nasmyth K. 1999. Sister-chromatid separation at anaphase onset is promoted by cleavage of the cohesin subunit Scc1. Nature 400:37–42.

77. Mascaraque V, Hernaez ML, Jimenez-Sanchez M, Hansen R, Gil C, Martin H, Cid VJ, Molina M. 2012. Phosphoproteomic analysis of protein kinase C signaling in Saccharomyces cerevisiae reveals Slt2 MAPK-dependent phosphorylation of eisosome core components. Mol Cell Proteomics2012/12/12.

78. Monteoliva L, Martinez-Lopez R, Pitarch A, Hernaez ML, Serna A, Nombela C, Albar JP, Gil C. 2011. Quantitative proteome and acidic subproteome profiling of Candida albicans yeast-to-hypha transition. J Proteome Res2010/12/08. 10:502–517.

79. Engholm-Keller K, Birck P, Størling J, Pociot F, Mandrup-Poulsen T, Larsen MR. 2012. TiSH - a robust and sensitive global phosphoproteomics strategy employing a combination of TiO2, SIMAC, and HILIC. J Proteomics 75:5749–5761.

80. Larsen MR, Thingholm TE, Jensen ON, Roepstorff P, Jørgensen TJD. 2005. Highly selective enrichment of phosphorylated peptides from peptide mixtures using titanium dioxide microcolumns. Mol Cell Proteomics 4:873–886.

81. Thingholm TE, Jensen ON, Robinson PJ, Larsen MR. 2008. SIMAC (sequential

elution from IMAC), a phosphoproteomics strategy for the rapid separation of monophosphorylated from multiply phosphorylated peptides. Mol Cell Proteomics 7:661–671.

82. Deutsch EW, Csordas A, Sun Z, Jarnuczak A, Perez-Riverol Y, Ternent T, Campbell DS, Bernal-Llinares M, Okuda S, Kawano S, Moritz RL, Carver JJ, Wang M, Ishihama Y, Bandeira N, Hermjakob H, Vizca??no JA. 2017. The ProteomeXchange consortium in 2017: Supporting the cultural change in proteomics public data deposition. Nucleic Acids Res 45:D1100–D1106.

83. Vizcaíno JA, Csordas A, Del-Toro N, Dianes JA, Griss J, Lavidas I, Mayer G, Perez-Riverol Y, Reisinger F, Ternent T, Xu QW, Wang R, Hermjakob H. 2016. 2016 update of the PRIDE database and its related tools. Nucleic Acids Res 44:D447–D456.

84. Chou MF, Schwartz D. 2011. Biological Sequence Motif Discovery Using motif-x. Curr Protoc Bioinformatics Chapter 13:Unit13.15.

85. Szklarczyk D, Morris JH, Cook H, Kuhn M, Wyder S, Simonovic M, Santos A, Doncheva NT, Roth A, Bork P, Jensen LJ, von Mering C. 2017. The STRING database in 2017: quality-controlled protein-protein association networks, made broadly accessible. Nucleic Acids Res 45:D362–D368.

86. Huang DW, Lempicki R a, Sherman BT. 2009. Systematic and integrative analysis of large gene lists using DAVID bioinformatics resources. Nat Protoc 4:44–57.

87. Alva V, Nam S-Z, Söding J, Lupas AN. 2016. The MPI bioinformatics Toolkit as an integrative platform for advanced protein sequence and structure analysis. Nucleic Acids Res 44:W410–W415.

88. Šali A, Blundell TL. 1993. Comparative Protein Modelling by Satisfaction of Spatial

Restraints. J Mol Biol 234:779–815.

89. Shendure J, Ji H. 2008. Next-generation DNA sequencing. Nat Biotechnol 26:1135–1145.

90. Mitternacht S. 2016. FreeSASA: An open source C library for solvent accessible surface area calculations. F1000Research 5:1–12.

91. Rodrigues JPGLM, Trellet M, Schmitz C, Kastritis P, Karaca E, Melquiond ASJ, Bonvin AMJJ. 2012. Clustering biomolecular complexes by residue contacts similarity. Proteins Struct Funct Bioinforma 80:1810–1817.

Figure legends

Figure 1. Potential substrates of PP2A^{Cdc55} phosphatase. (A) The normalized heavy/light (H/L) ratio of all phosphopeptides. The number of phosphopeptides (n=1260) with H/L ratios <0.75 (corresponding to the hyperphosphorylated peptides) is shown. (B) Frequency distribution of the H/L ratios from an aliquot of the whole protein extracts before phosphopeptide enrichment. The amount of protein is unchangeable for most of the peptides. Red lines mark the lower and upper limits, which are set to 0.75 (log2=-0.41) and 1.3 (log2=0.41), respectively. (C) Distribution of the Ser, Thr and Tyr residues among the hyperphosphorylated peptides in the *cdc55Δ* mutant. All the peptides (10,069) identified in our 3 SILAC approaches were used as background. (D) Distribution of the S/TP sites within the hyperphosphorylated peptides. (E-F) Venn diagrams representing overlapping hits from the three approaches, for both hyperphosphorylated peptides and proteins. (G) Gene name corresponding to the overlapping proteins identified. (H) Volcano plot representing the common phosphopeptides among the three approaches generated from two-tailed Student’s t-test ($p<0.05$).

Figure 2. Consensus phosphorylation sites found hyperphosphorylated in absence of PP2A$^{\mathbf{Cdc55}}$. (A) Motifs logo found using Motif-X, for either central residue phospho-Serine or phospho-Threonine. (B) Phosphomotif consensus sequence, motif score and fold increase for each consensus motif. (C) Common elements between Cdk1 and PP2A^{Cdc55} targets. Venn diagrams, GO processes from the common Cdk1-PP2A^{Cdc55} targets and Common targets are shown.

Figure 3. The Interaction Network analysis identified 6 protein nodes related to PP2A-Cdc55. Distribution of the number of interactions identified 6 protein nodes with more than 16 interactions. The proteins present in these 6 protein nodes with 16 or more interactions are shown.

Figure 4. In vivo validation of PP2A$^{\mathbf{Cdc55}}$ novel substrates. (A) Summary of already known PP2A^{Cdc55} substrates identified in our SILAC experiments. (B) Validation of PP2A^{Cdc55} substrates. Strains Y1223 (*MAT a LTE1-3PK::LEU2 MET-CDC20::LEU2*), Y1224 (as Y1223, but *cdc55Δ*), Y1240 (*MAT a RTS1-6PK::TRP1 MET-CDC20::LEU2*), Y1241 (as Y1240, but *cdc55Δ*), Y1277 (*MAT a SLK19-*HA_6*::HIS3 MET-CDC20::LEU2*) and Y1278 (as Y1277, but *cdc55Δ*) were arrested in metaphase by Cdc20 depletion and synchronously release in anaphase by Cdc20 re-introduction. Lte1, Rts1 and Slk19 phosphorylation status were identified by western blot. Native protein extracts from metaphase samples were treated with alkaline phosphastase (CIP lane) as dephosphorylation controls. (C) Proteins identified as PP2A^{Cdc55} physical-interactors

proteins after TAP purification experiments. Protein extract from Y614 strain containing a TAP-Cdc55 (*MAT a*, *CDC14-HA$_6$::HIS3 TAP::CDC55 GAL1-CDC20::URA3*) was prepared and TAP purification assay was performed as described in methods. (D) Proteins identified phosphorylated and co-eluted with HA-Cdc55. Protein extract from Y2541 strain containing an HA-Cdc55 (*MAT a HA::CDC55 GAL1-CDC20::LEU2*) was prepared, HA-Cdc55 was purified and phosphopeptide enrichment was performed as described in methods.

Figure 5. Docking models of PP2A^{Cdc55} and Mob1 highlight potential binding interfaces for Cdc55 and Mob1

(A) Representatives of the best 10.000 models of the CDC55/Mob1 complexes superimposed on the human heterotrimeric PP2A structure (PDB 3dw8). Red spheres represent the centers of mass of representative models. The regulatory B55 subunit, homologous to Cdc55, is shown in green, while the catalytic subunit is shown in blue. Residues previously identified as interacting with Tau are represented as green spheres. (B) Representatives of the filtered subset of 294 models of Cdc55/Mob1, after filtering for catalytic subunit distance. (C) and (D) Per-residue interface propensities (log2 scaled, red showing higher values) calculated on 294 filtered models of Cdc55/Mob1, respectively.

List of Additional files

Additional file 1.pdf

Workflow for SILAC analysis of PP2A-Cdc55 dependent phosphoproteome. Three different methods were used for phosphopeptide enrichment: SIMAC, TiO2 and TiSH-

based approach. A detailed scheme of each methodology is presented. LC-MS/MS analysis of the eluted fractions was performed in order to identify and quantify the heavy/light labelled peptides. Identification and quantification was analysed using Proteome Discoverer.

Additional file 2.xlsx

Hyperphosphorylated peptides corresponding to putative PP2A-Cdc55 regulated proteins. List of the 1,260 quantified hyperphosphorylated peptides identified in our three SILAC experiments.

Additional file 3.pdf

Common peptides and proteins quantified in the whole cell extract and in the hyperphosphorylated list. List of the 286 matching proteins identified in the whole cell extract (non-enrich analysis) and in our hyperphosphorylated dataset. All the matching proteins had similar protein abundance between the wild type and the *cdc55*Δ mutant (heavy/light ratio >0.8 in the non-enriched analysis).

Additional file 4.pdf

Common peptides found in the phosphoproteomic study. List of the hyperphosphorylated peptides found in the three different phospho-enrichment approaches.

Additional file 5.xlsx

Hyperphosphorylated peptides with a pRS probability >95%. List of hyperphosphorylated peptides containing residues identified with very high confidence

(peptides with a pRS probability > 95%). We identified 721 unique hyperphosphorylated peptides containing 562 unique phosphomotifs.

Additional file 6.xlsx

Gene Ontology of the PP2A-Cdc55 potential substrates. The gene ontology terms of proteins displaying enhanced phosphorylation in our dataset are summarized in the Non-Clustered sheet and the functional clustering of the GO terms are summarized in the Clustered sheet.

Additional file 7.pdf

String Network analyses of the hyperphosphorylated proteins identified in our dataset. Interactions found for each protein was plotted. A magnification of the Cdc28 and Cla4 nodes is shown.

Additional file 8.pdf

Proteins identified in two TAP-Cdc55 purification assays. List of proteins identified in the two TAP-Cdc55 pull-downs that are not found in the negative control purification. A strain without the TAP epitope was used as negative control.

Additional file 9.pdf

Proteins identified in the HA-Cdc55 purifications. Proteins and peptides identified after HA-Cdc55 purification using HA-affinity columns. The eluted fractions were subjected to TiO_2 enrichment to search for proteins that are undergoing phosphorylation modifications

among the newly identified Cdc55 associated proteins. Peptide and protein modifications were obtained using the Mascot search engine.

Additional file 10.pdf

Proteins and peptides identified containing a Cdc5 consensus site. List of proteins from our PP2A-Cdc55 phosphoproteome dataset containing the D/E/N-x-S/T Cdc5 polo-like kinase consensus sites. We identified 161 phosphopeptides corresponding to 140 unique proteins.

Kinase found	Type of kinase	consensus motif	biological process	susbtrates found
ATG1	S/T	LM-X-X-s-X-FIV	vesicle formation, autophagy	-
BCK1	S/T - MAP	P-X-s-P	MPAKKK of PCK - cell wall integrity pathway	-
CBK1	S/T - NDR/LATS	H-X-R-R-X-s	polarized growth, cell separation, and cell integrity	Ssd1, Ace2
CDC28	S/T	sP	Cell cycle	many
CLA4	S/T - PAK	RK-R-X-s	cytokinesis, vacuole inheritance	Shs1, Nap1, cdc3
CMK2	S/T - Calmodulin	KR-X-X-s	stress response	-
KCC4, GIN4	S/T	-	bud growth, septin ring assembly	Shs1, Nap1
MCK1	S/T	s-X-X-X-s-P	chromosome segregation, meiotic entry, genome stability, transcriptional regulation	-
NPR1	S/T	KR-X-X-s-KR	regulation of plasma membrane transporters	Ldb19
PBS2	S/T - MAP	P-X-s-P	MAPKK of Hog pathway	-
PKC1	S/T	R-X-X-s	cell wall integrity pathway	Bck1
PKH1	S/T	-	cell wall integrity, endocytosis	Ypk1, Ypk2
PSK1	S/T - PAS	-	carbohidrate metabolism	-
RIM15	S/T - PAS	-	establishment of stationary phase	Igo1
SSK1	S/T - MAP	P-X-s-P	osmosensing	Pbs2
SSK2	S/T - MAP	P-X-s-P	MAPKKK of Hog pathway, actin cytoskeleton recovery	Pbs2
STE20	S/T - PAK	RK-R-X-s	pheromone response, vacuole inheritance, sterol uptake	-
YCK2	S/T - Casein	-	endocytsis, septins regulation	-
YCK3	S/T - Casein	-	vacuole fusion	-
YPK1, YPK2	S/T	-	cell wall integrity pathway, lipid metabolism	Gpd1

Table 1. Kinases and their substrates found in our phosphoproteomic study.

GO Category		Genes
Cell cycle	135	*SSK2, SCP160, MSC3, BUD14, SAS10, VPS13, NSP1, SIF2, BCK1, CMD1, SYP1, RFA2, STB1, CDC37, VPS1, LRS4, PCL6, MDS3, BNI5, SMI1, SNT1, BUD3, HOS3, VPS54, WHI3, MSO1, STE50, SLI15, SIS2, ORC4, SET2, POL1, PAT1, LDB19, OPY2, PCL7, YOX1, SAP155, IQG1, LTE1, SIC1, ASH1, YRB1, RIM15, TGS1, GCS1, GRR1, RTT107, MSS4, BNI1, RAD9, TOP1, TUP1, ASM4, STE20, MSG5, PMD1, MMR1, HSL1, SKG3, TFB3, SWI6, VRP1, SPO14, SET1, SET3, CDC13, SGT1, RGP1, RFA1, WHI5, RCK2, CBF2, SHP1, RFC1, RSC2*
Mitotic cell cycle	(83)	*BIR1, SAP155, KEL2, BIM1, LTE1, NET1, TOP2, SIC1, ASH1, YRB1, KIN2, PKC1, RIM15, KIN1, GRR1, CLA4, RAD9, BNI1, TOP1, TUP1, ASM4, STE20, SRC1, ULP1, RTS1, SPA2, HSL1, SKG3, STB1, HPC2, PAN1, SPC105, PBS2, SLK19, VRP1, SWI6, BUD6, CDC14, BNI4, ACE2, BUD3, KIN4, PIN4, HOS3, PEF1, SAC3, CDC12, SUM1, STU2, MBP1, GIN4, SHS1, KEL1, SSD1, PAF1, CDC28, KIP2, RRD1, CDC25, SLA2, SWI5, RGP1, FAR11, INN1, WHI5, PDS5, SWI4, CYC8, CBF2, SPT6, SPC19, STH1, PTK2, CDC3, KCC4, RFC1, CBF5, AXL2, UME6, CDC11, VHS2, YOX1*
Cytokinesis	(23)	*BNI1, PAN1, INN1, STE20, VRP1, BUD6, CDC14, BNI4, NET1, MYO2, BUD3, RTS1, SPA2, EDE1, PEF1, CDC3, CDC12, PKC1, AXL2, SHS1, CDC11, VHS2, SLA2*
Cytoskeleton organization	64	*BIR1, BIM1, MYO2, YRB1, BEM3, MSS4, SAC7, CMD1, RTS1, CDC37, MHP1, YTA6, SPC105, NUM1, BNI5, CDC14, CDC12, STU2, GIN4, CDC28, KIP2, NAP1, LIA1, CBF2, SPC19, STH1, CDC3, KCC4, VHS2*
Actin cytoskeleton organization	(36)	*PBS2, PAN1, IQG1, KEL2, SSK2, ABP1, VRP1, BUD6, RGA1, BUD14, CBK1, ARK1, AVO2, AKL1, ENT1, PKC1, SHS1, KEL1, TSC11, GCS1, YSC84, MSS4, SLA2, CLA4, BNI1, STE20, TWF1, BIT61, BEM2, BBC1, SPA2, CRN1, SYP1, VPS1, SSK1, ENT2*
Vesicle-mediated transport	83	*INP52, LTE1, BOI2, VPS9, MDR1, MYO2, ARK1, AKL1, UBP3, PIL1, KIN2, MUK1, KIN1, SEC16, GCS1, MON2, GLO3, YCK2, YCK1, GTS1, INP53, SNF7, ECM21, VPS17, APL5, DNF2, PKH1, ROM2, CMD1, RCR2, SYP1, VPS1, ENT2, GRH1, NPR1, SEC21, PAN1, BRE4, SMY1, SMY2, FTH1, PIB2, VRP1, SSA1, DNF1, GYP1, GGA1, SPO14, YPT31, ELO2, RAV1, EDE1, PEF1, ENT1, YPK1, ENT5, APM4, VPS54, CDC28, YSC84, SEC2, SNX41, SLA2, MSO1, RGP1, DDI1, OSH2, SWA2, ROD1, SFB3, SEC10, OSH3, SEC9, YCK3, SWH1, VPS53, YKR078W, PAL1, LDB19, GYP5, PIK1, SEC31, LAA1*
Endocytosis	41	*PAN1, BRE4, PIB2, FTH1, VRP1, INP52, DNF1, SPO14, EDE1, ENT1, YPK1, PIL1, APM4, YSC84, MON2, YCK2, SNX41, YCK1, SLA2, GTS1, SWA2, OSH2, INP53, ECM21, ROD1, VPS17, DNF2, OSH3, PKH1, YCK3, ROM2, CMD1, SWH1, SYP1, YKR078W, PAL1, VPS1, LDB19, PIK1, ENT2, NPR1*
Protein phosphorylation	50	*CMK2, PAN3, SSK2, TDA1, SNF1, MEH1, SIC1, ARK1, YAK1, AKL1, PIL1, NNK1, PKC1, KIN2, KIN1, RIM15, RTK1, YCK2, YCK1, CLA4, STE20, MSG5, SIF2, PKH1, BCK1, HSL1, CDC37, STE5, NPR1, PCL6, PBS2, SCY1, TFB3, CTK3, PSK1, CBK1, SNT1, NBP2, KIN4, YPK1, CDC12, GIN4, PAF1, CDC28, PRR1, YPK2, RCK2, HRK1, SLI15, STE50, YCK3, PTK2, KCC4, SIP2, SSK1, CDC11, PCL7, SKY1*
Establishment or maintenance of cell polarity	33	*PAN1, SCS2, VRP1, DNF1, BUD6, RGA1, CBK1, MYO2, BUD3, BOI2, AVO2, PEF1, CDC12, SHS1, BEM3, TSC11, SLA2, BNI1, OSH2, STE20, MSB1, PXL1, BIT61, DNF2, OSH3, BEM2, BCK1, ROM2, SPA2, CDC3, SWH1, AXL2, CDC11*
Cell budding	21	*BNI1, PAN1, NAP1, STE20, VRP1, TGL4, BUD6, RGA1, CBK1, MYO2, BOI2, BUD3, CMD1, SPA2, PEF1, KCC4, AXL2, GIN4, CDC28, SLA2, BOI1*

Table 2. Major Gene Onthology categories of all hyperphosphoproteins identified in the three approaches.

Click here to download Figure Figure1.pdf

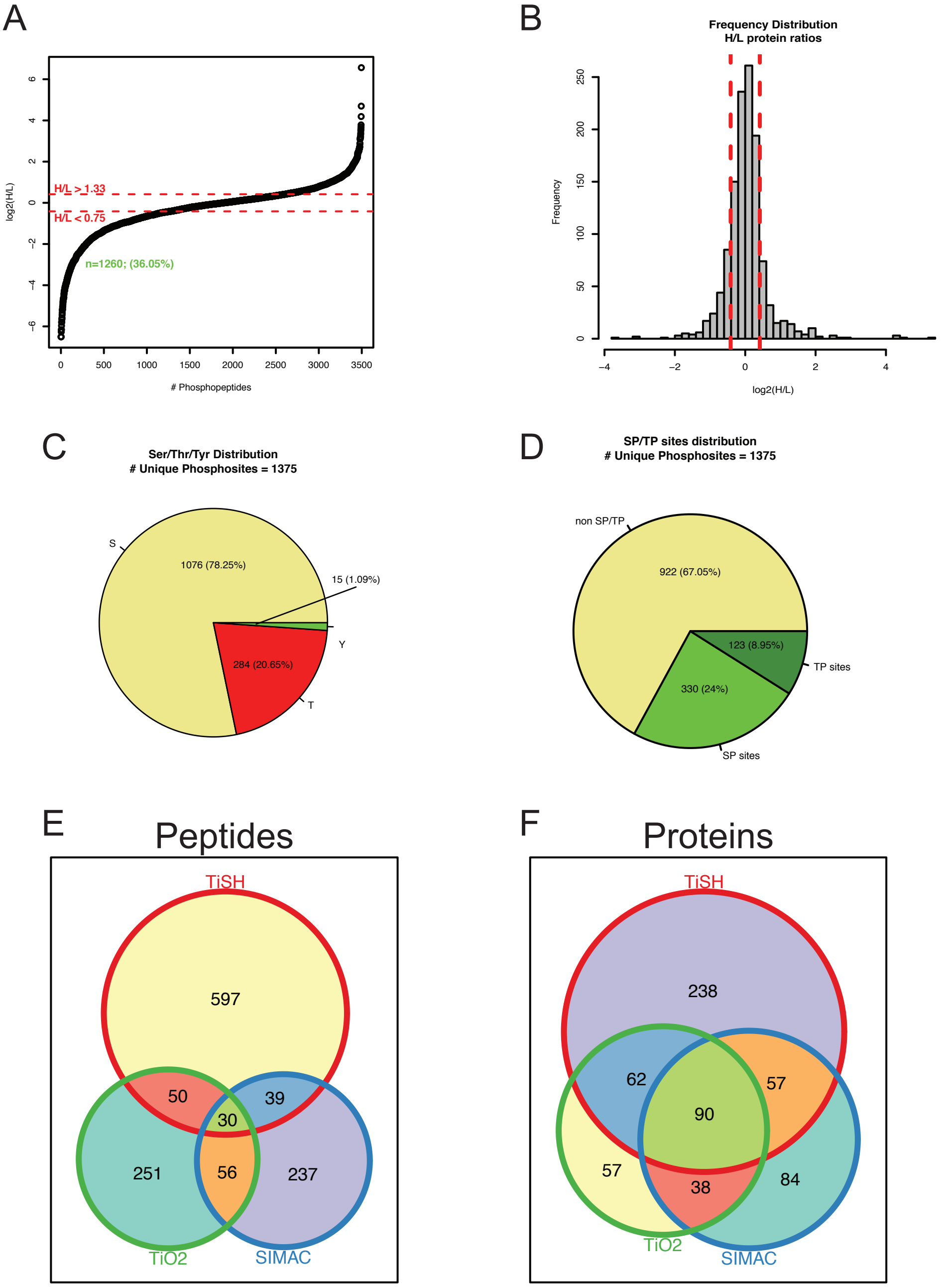

G

Common Proteins						
M1+M2+M3		M1+M2	M1+M3		M2+M3	
ABP1	PAR32	BRE4	ARG82	STB3	ACF4	STB1
ACE2	PBP1	CHS1	ARP8	SWI5	APA1	SWA2
AIM21	PEX19	CRN1	ASK10	TGL1	ASM4	TAT1
AIM3	PFK2	DCP2	ATG13	TPS3	AXL2	TDA11
AKL1	PFK26	DIP5	BNI1	VBA4	BAP2	TIF5
ALE1	PIN4	ECM21	BUD3	VIP1	BCK1	TSL1
ATG1	PRS5	ELO2	CDC25	VPS54	BCY1	TY2A-GR1
BBC1	PSH1	FLC2	CWC24	YBR197C	BLM10	VHS2
BDF1	PTK2	FLC3	DIG1	YCL012C	BOI1	VRP1
BIM1	PXL1	GCD6	DNF2	YDR186C	CDC37	WHI5
BNI5	RAD7	GPD1	DNM1	YFR016C	CYR1	YHR097C
BOI2	REG1	HHO1	EAP1	YSP2	DSF2	YIL055C
BUD6	RGT1	HXK1	FAR11		EDE1	YMR102C
CDC3	RPG1	HXT2	FBA1		ESC1	YMR196W
CLA4	RPH1	INN1	FHL1		ESF1	YPR091C
CRP1	SCY_3392	IVY1	FLC1		FAB1	YPR172W
DCP2	SCY_4679	KIP2	GIS1		FOL2	ZRC1
EDC3	SEC2	MON2	GLC8		FYV8	
ENT1	SEC31	MSL5	GTT3		GRH1	
GAT1	SFL1	MTC1	GYP5		GSY2	
GCS1	SHP1	PBS2	HOS3		GTS1	
GFD1	SIP1	PDR5	IGO1		GYP1	
GGA1	SIS2	PEX30	IML1		HEL2	
GIN4	SLF1	PTR2	LEO1		HER1	
GIP4	SMY2	RHO5	MBF1		HRB1	
GPD2	SPA2	RPL7A	MDS3		HXT1	
GPR1	SRC1	SDS23	MIG1		INP52	
GPT2	SSD1	SEG2	MOT1		INP53	
HOM3	SSK2	SET2	MSN4		LYP1	
HSL1	STE20	TCB3	MYO2		MRH1	
HSP12	STU2	TRM2	NPR2		MSS4	
KEL1	SUI3	UTP22	NUP159		NAP1	
KIN1	SUM1	VID27	OPY2		NNK1	
KIN2	SWH1	VPS13	PAM1		NOP13	
LAM5	TMA17	YCF1	PIB2		NUP2	
LSB3	TSC11	YGR054W	PKC1		PIK1	
MAM3	UBX7	YGR130C	PSK1		POP2	
MDG1	VNX1	YGR149W	ROD1		RCR2	
MSC3	WHI3		RSC2		RIM15	
NOT3	YLR257W		SAC7		SCP160	
NOT5	YLR413W		SEC9		SDS24	
NPR1	YOL019W		SHS1		SEG1	
NSG2	YRB1		SLI15		SLA2	
PAL1	YSC84		SNU114		SNF1	
PAN1	ZEO1		SPO14		SSK1	

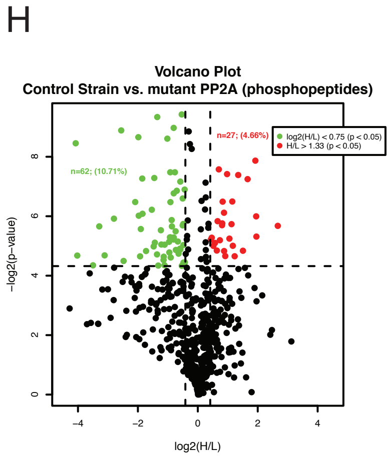

Figure2

Click here to download Figure Figure2.pdf

A

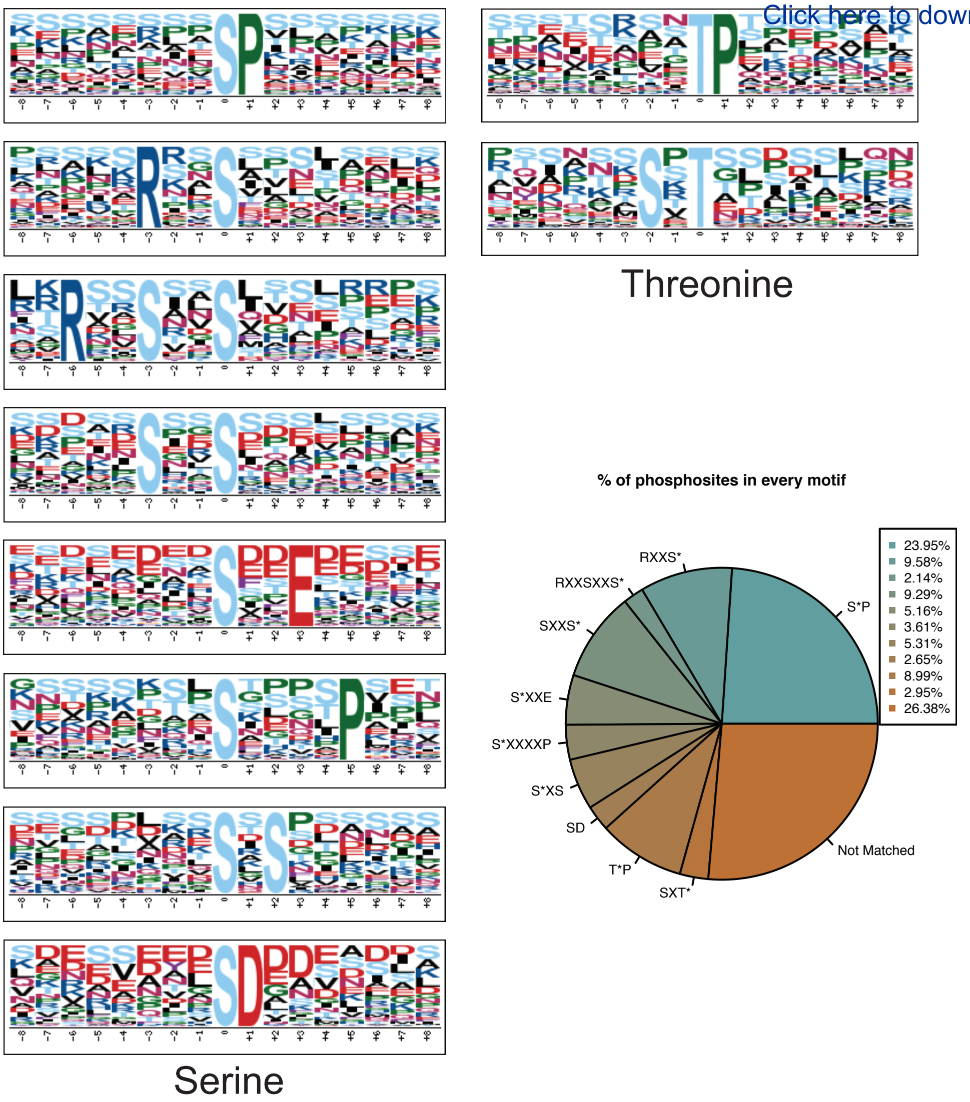

B

#	Motif	Motif Score	Foreground Matches	Foreground size	Background Matches	Background Size	Fold Increase
1.	........SP.......	16.00	325	1060	10410	237630	7.00
2.	.....R..S........	16.00	130	735	10661	227220	3.77
3.	..R..S..S........	24.82	29	605	1344	216559	7.72
4.	.....S..S........	11.82	126	576	24855	215215	1.89
5.	........S..E.....	10.19	70	450	12776	190360	2.32
6.	........S....P...	10.02	49	380	8108	177584	2.82
7.	........S.S......	8.14	72	331	18359	169476	2.01
8.	........SD.......	6.71	36	259	8100	151117	2.59

#	Motif	Motif Score	Foreground Matches	Foreground Size	Background Matches	Background Size	Fold Increase
1.	........TP.......	16.00	122	282	7896	154521	8.47
2.	......S.T........	7.72	40	160	14254	146625	2.57

C

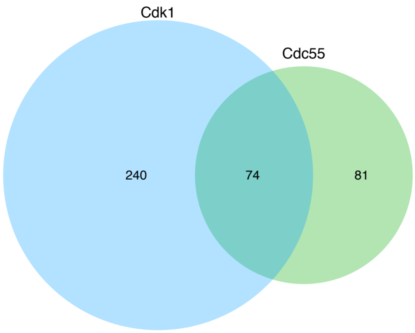

GO_term	P-value
cell cycle	5.95e-10
mitotic cell cycle process	2.26e-9
cell cycle G1/S transition	7.98e-8
biological regulation	1.44e-8
cellular component organization	3.87e-6
organelle organization	4.73e-6
cytokinesis	1.28e-5

Common elements in Cdk1 and Cdc55			
ABP1	GFD1	PIN4	SSD1
ACE2	HER1	POL1	SSK2
ACF4	INP53	PSP2	SSN2
ASH1	ISW2	PTK2	STB1
ASK10	KEL1	REG1	STE20
AVO2	KIN2	RIM15	SWI4
BAP2	LEO1	RSC2	SWI5
BEM3	MDS3	RTS1	TCB3
BNI4	MLF3	SAC3	TCO89
BOI1	MSC3	SAC7	TIF4632
BRL1	MSL5	SDS24	TOP2
CDC3	NET1	SEC10	TSL1
CLA4	NOT5	SEC31	VRP1
ECM21	NTE1	SHS1	WHI5
EDE1	NUP159	SIR4	YER079W
FAB1	NUP60	SIS2	YMR196W
FLC1	ORC4	SLA1	YPR091C
FUN19	PAL1	SPA2	
GCS1	PAR32	SRC1	

Figure 2

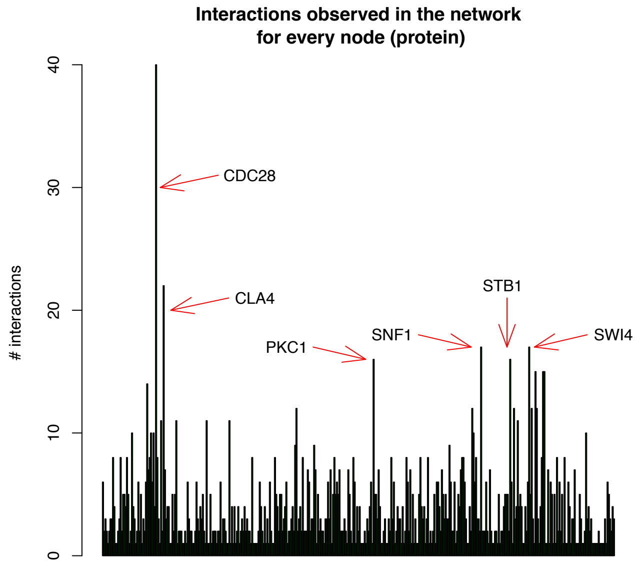

Protein Node		Proteins
Cdc28	40	Ace2, Ash1, Bni1, Bud3, Bud6, Cdc13, Cdc14, Cdc28, Cdc37, Fir1, Gin4, Grr1, Hho1, Hsl1, Kip2, Lte1, Mbp1, Net1, Pah1, Pcl6, Pcl7, Pol1, Ptk2, Pxl1, Rad9, Rim15, Sic1, Skg3, Sli15, Snf1, Srl3, Stb1, Ste20, Swi4, Swi5, Swi6, Tfb3, Tfb6, Ubp3, Whi5, Yox1
Cla4	22	Bck1, Bem3, Bni1, Bud6, Cdc12, Cdc14, Cdc3, Gin4, Hsl1, Kin4, Myo2, Nbp2, Rga1, Rho5, Rts1, Rtt107, Slk19, Smy1, Spa2, Ste20, Swi4, Vac14
Pkc1	16	Bck1, Bni1, Cyr1, Mbp1, Mig1, Pah1, Pkh1, Rho5, Smi1, Spa2, Spo14, Ssd1, Sth1, Swi4, Tsc11, Ypk1
Snf1	16	Acc1, Cdc28, Ctk3, Cyc8, Ena1, Mig1, Msn2, Reg1, Rod1, Sip1, Ssn2, Swi6, Tfb3, Tfb6, Tup1, Ypk2
Stb1	16	Ash1, Cdc28, Mbp1, Set3, Sif2, Snt1, Swi4, Swi6, Taf12, Taf5, Tfa2, Tfb3, Tfb6, Tfg1, Ume6, Whi5
Swi4	17	Bck1, Cdc28, Chd1, Cla4, Mbp1, Paf1, Pdr1, Pkc1, Ssd1, Stb1, Swi6, Taf12, Taf5, Tfb3, Tfb6, Tfg1, Whi5

Figure 3

Figure4
Click here to download Figure Figure4.pdf

A

Substrate	Our SILAC	Reference
Cdc28-Y19	Yes	Yang et al., 2000
Net1	Yes	Queralt et al., 2006
Mob1	medium confidence peptides, not quantified	Baro et al., 2013
Gis1	Yes	Bontron et al., 2013
Bfa1	medium confidence peptides, not quantified	Baro et al., 2013
Whi5	Yes	Talarek et al., 2017
Scc1	No peptide found	Yakoov et al., 2012
Cdc16	No peptide found	Vernieri et al., 2013

B

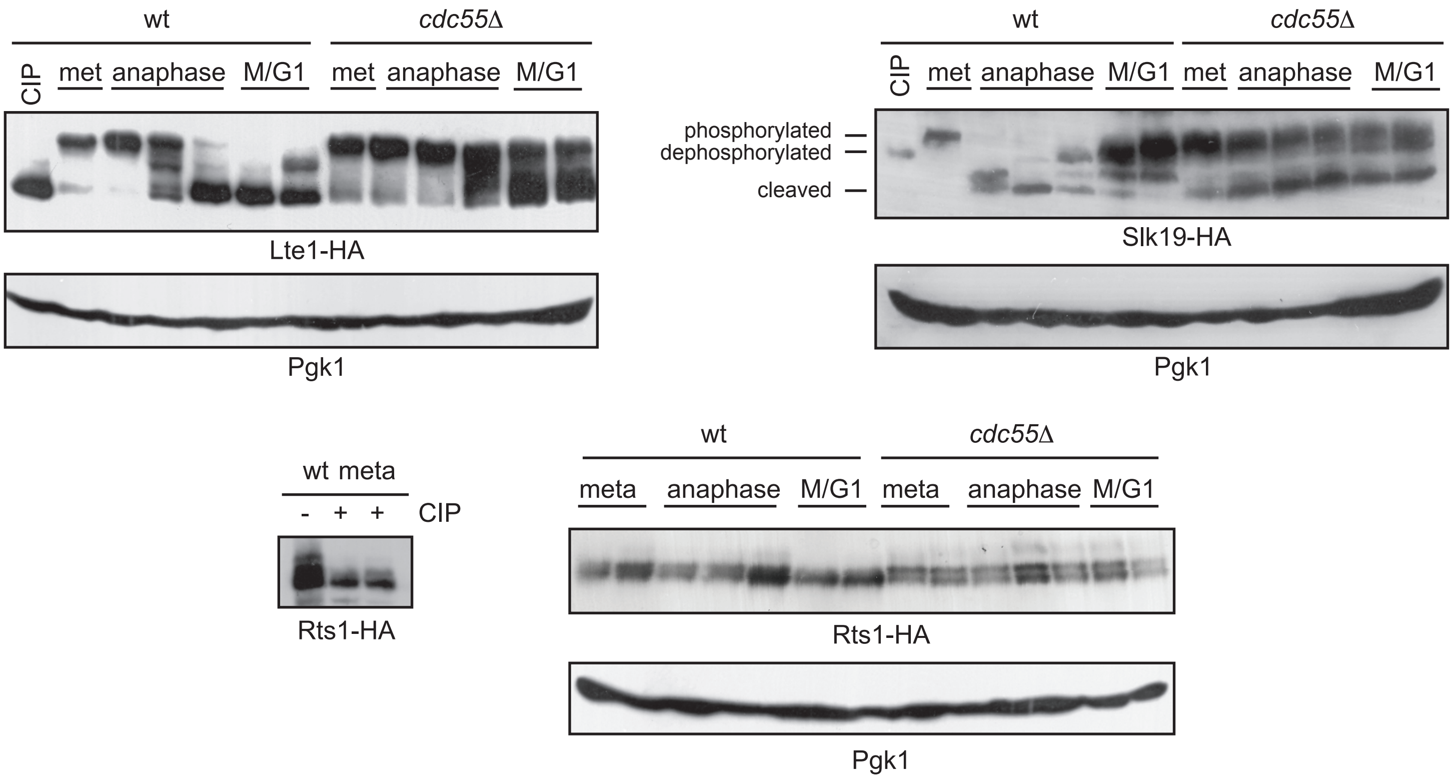

C

Protein	TAP purification
Apa1	TAP-Cdc55 (2), 23 peptides identified
Dnm1	TAP-Cdc55 (2), 8 peptides identified
Set1	TAP-Cdc55 (2), 1 peptides identified
Zeo1	TAP-Cdc55 (1), 4 peptides identified

D

Protein	Peptide sequence	Modification
Tgl1	QLDANSsTTALDALNKE	Phosphorylation
Psh1	NSALAVADDsDDGITR	Phosphorylation

Figure 4

 Click here to download Figure Figure5.pdf

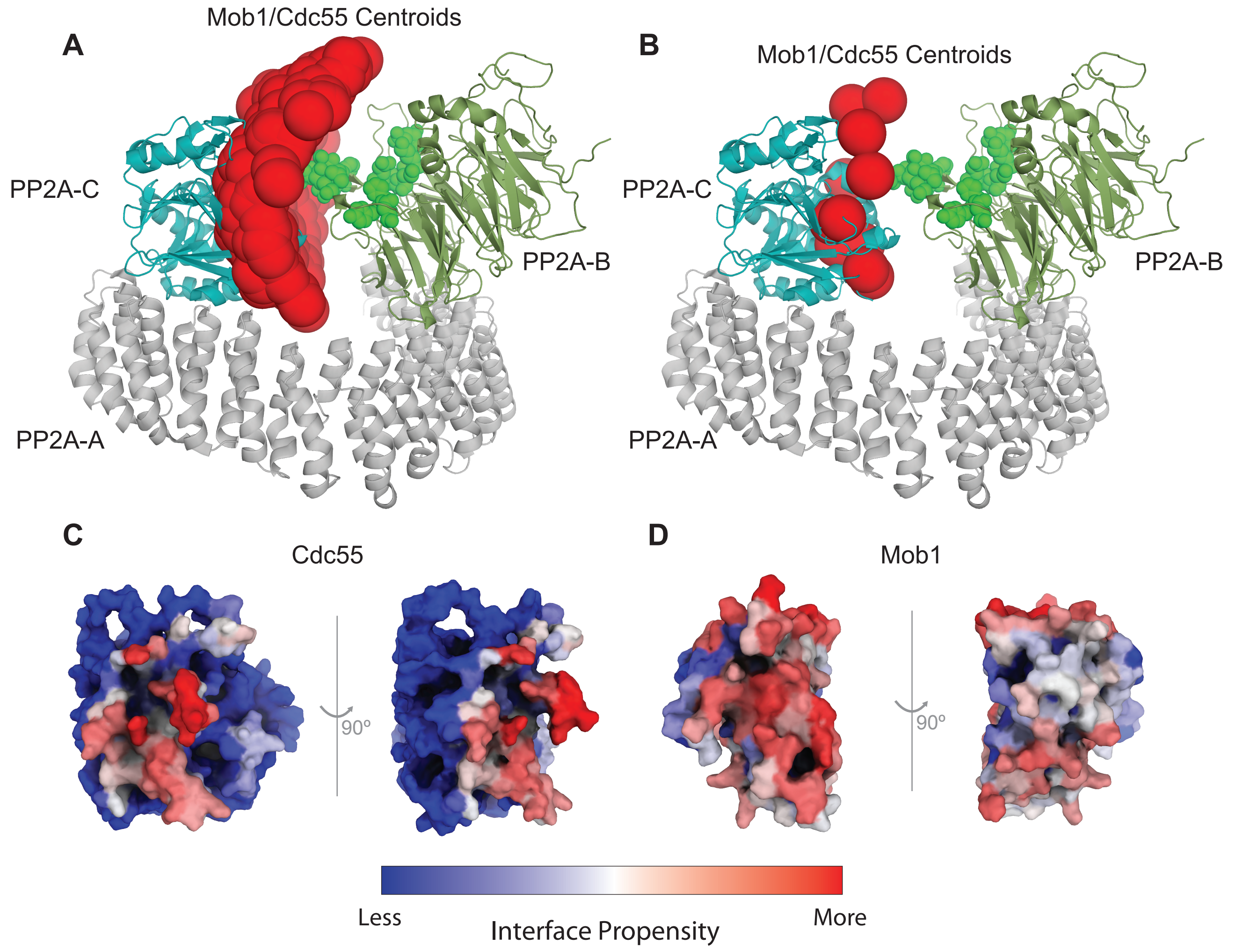

Figure 5

Supplementary Material1

Click here to access/download

Supplementary Material

Additional file 1.pdf

Click here to access/download
Supplementary Material
Additional file 2.xlsx

Supplementary Material3

Click here to access/download
Supplementary Material
Additional file 3.pdf

Click here to access/download
Supplementary Material
Additional file 4.pdf

Click here to access/download
Supplementary Material
Additional file 5.xlsx

Supplementary Material6

Click here to access/download
Supplementary Material
Additional file 6.xlsx

Supplementary Material7

Click here to access/download

Supplementary Material

Additional file 7.pdf

Click here to access/download
Supplementary Material
Additional file 8.pdf

Click here to access/download
Supplementary Material
Additional file 9.pdf

Supplementary Material10

Click here to access/download
Supplementary Material
Additional file 10.pdf

IDIBELL
Institut d'Investigació Biomèdica de Bellvitge

Dr. Ethel Queralt
Head, Cell Cycle Laboratory
Cancer Epigenetics and Biology Program (PEBC)
Bellvitge Institute for Biomedical Research (IDIBELL)
Avinguda Gran Vía de l'Hospitalet, 199-203,
08908, Hospitalet de Llobregat
Barcelona

equeralt@idibell.cat
tel +34 932607128
fax +34 932607219
www.pebc.cat; www.idibell.cat

Gigascience
Oxford University Press

Barcelona, 26 September 2017

Dear Editors,

Please find enclosed our manuscript, Baro et al. "SILAC-based phosphoproteomics reveals new PP2A-Cdc55-regulated processes in budding yeast", that we would like to submit for publication in the Gigascience journal as an original research article.

PP2A is a family of conserved serine/threonine phosphatases that has been linked to a wide variety of human diseases due to its prominent functions in cell growth and proliferation. $PP2A^{Cdc55}$ phosphatase, and its homolog in higher eukaryotes, PP2A-B55, have been extensively related to cell cycle regulation; however, few $PP2A^{Cdc55}$ substrates have been identified until date. In this study, we performed a systematic quantitative phosphoproteomic analysis of $PP2A^{Cdc55}$ deficient cells in order to identify novel $PP2A^{Cdc55}$ substrates and regulated processes in budding yeast. We used three different techniques (SIMAC, TiSH and TiO2) to purify and identify phosphoproteins and phosphopeptides. Every method has a bias, therefore using three alternative approaches to purify phosphopeptides we were able to get a broader phosphopeptide spectrum.

While preparing this manuscript, another SILAC-based study targeting $PP2A^{Cdc55}$ phosphatase was published using TiO2 phosphopeptide enrichment (Godfrey et al., Mol. Cell 2017). By comparing the phosphorylation status of Cdk1 substrates in absence of $PP2A^{Cdc55}$ at different cell cycle phases (G1, S and M), they deciphered how $PP2A^{Cdc55}$ contributes to determine progressive phosphorylation of Cdk1 substrates. In contrast, our study focused in metaphase-arrested cells (M), and we considered not only the Cdk1-counteracted substrates but all Cdc55-dependent phosphoproteome for downstream analysis. We found Cdk1,

Cla4 and Pkc1 as important nodes of interaction in the Cdc55 phosphoproteome. We also validated Slk19 and Lte1 substrates, building on our previous work on $PP2A^{Cdc55}$ function in early- and late-anaphase regulatory pathways (Queralt et al. 2006, Calabria et al. 2012, Baro et al. 2013). We thus provide novel conclusions and contribute to the understanding of $PP2A^{Cdc55}$-dependent substrates and pathways.

This study, thus, provides a significant extension of our understanding of the principles of the cell cycle processes regulated by $PP2A^{Cdc55}$, an important enzyme related to essential cellular processes and to many human diseases. We expect that it will be of considerable interest to the broad readership of the Gigascience journal.

Please note that the mass spectrometry proteomics data have been deposited to the ProteomeXchange Consortium (81) via the PRIDE (82) partner repository with the dataset identifier PXD007613 (**Username:** reviewer50711@ebi.ac.uk, **Password:** Tv9GFPI2).

The content of the manuscript has not been published or submitted for publication elsewhere and all authors have approved the manuscript for submission.

Thank you very much for considering our submission, we look forward to hearing from you soon.

With best regards

Ethel Queralt PhD.
Cell Cycle group
