# Supplementary figures and images for: SILAC-based phosphoproteomics reveals new PP2A-Cdc55-regulated processes in budding yeast
Source: Gigascience. 2018 May 24;7(5):giy047. doi: 10.1093/gigascience/giy047 (PMC5967524; doi:10.1093/gigascience/giy047)

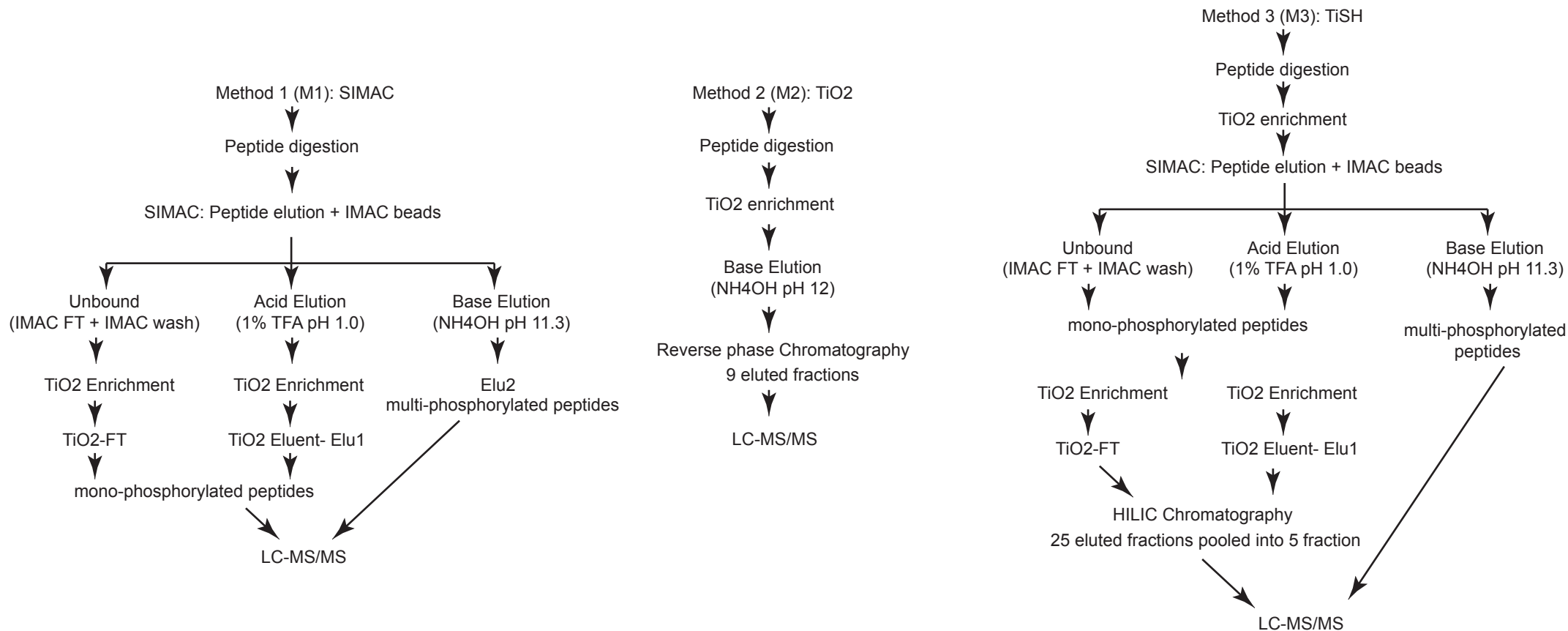

Supplement: Additional Files [file giy047_supp.zip › Additional file 1.pdf]

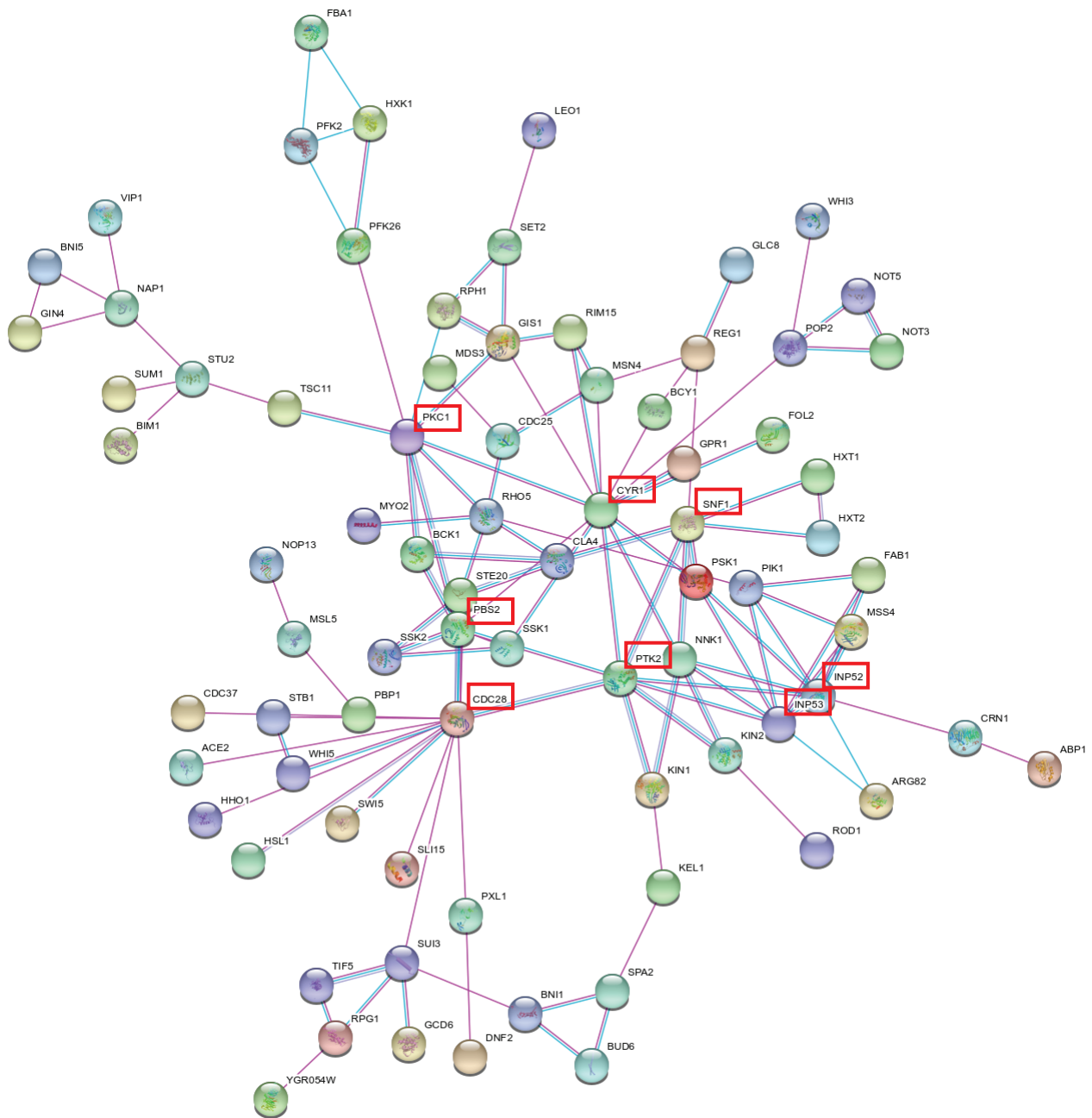

Supplement: Additional Files [file giy047_supp.zip › Additional file 9.pdf]
