# Supplementary material for: SILAC-based phosphoproteomics reveals new PP2A-Cdc55-regulated processes in budding yeast
Source: Gigascience. 2018 May 24;7(5):giy047. doi: 10.1093/gigascience/giy047 (PMC5967524; doi:10.1093/gigascience/giy047)
Supplement: Response_to_Reviewer_Comments_Original_Submission.pdf [file giy047_response_to_reviewer_comments_original_submission.pdf]

Author's Response To Reviewer Comments

Close

Reviewer #1: The manuscript by Baro et al, describes the use of 3 different SILAC based proteomic approaches combined with a powerful yeast genetic deletion model. The goal of the project was to identify possible substrates of the PP2A-B55/cdc55 phosphatase. To do this the authors arrested wild-type and Cdc55 deletion mutants in metaphase by also deleting cdc20. The only issue with this approach is that during metaphase PP2A-cdc55 activity is suppressed and the majority of all phosphorylation sites would already be maximally phosphorylated (Olsen et al., 2010). Hence the ability to further enhance phosphorylation during this time is likely to be limited and therefore the increase observed is likely a under representation of the true number of PP2A specific phosphorites.

We have reported previously that budding yeast PP2A-Cdc55 phosphatase activity is high in metaphase and it is inactivated during anaphase (Queralt et al 2006, Queralt and Uhlmann 2008), using the exactly same set-up arresting cells in metaphase by Cdc20 depletion. Under this conditions, we can detect hyperphosphorylation events of PP2A-targets. In fact, this is a well established system in our lab that allowed us to study deeply three candidates substrates Net1 (Queralt et al 2006) and Bfa1 and Mob1 (Baro et al 2013). In the current work we have extended this laboratory system to a global proteomic study.

This likely explains why only 27 sites were specifically increased, while 62 were decreased (Fig 1H).

We are really sorry since we noticed that we have failed to properly explain how we made the phosphopeptide identification. We have amended that in the manuscript and introduce further explanation in Results and Materials sections.
Combining the three methods, we identified 1260 high confidence phosphopeptides with increased phosphorylation (Heavy (wt)/light (cdc55mutant) ratio < 0.75) in the absence of Cdc55 phosphatase; therefore likely to be PP2A-Cdc55 substrates. Those 1260 hyperphosphorylated peptides include 62 (increased) phosphopeptides that were identified in at least 2 (out of 3) enrichment methods (Figure 1H) with a t-test p-value <0.05. Therefore, considering the different nature of the phosphopeptides identified for each method, the 62 phosphopeptide are pretty likely to be PP2A-Cdc55 substrates.

Nevertheless, we cannot consider the remaining 1198 hyperphosphorylated peptides to be all false positives (some phosphopeptides were identified with highly amounts of PSM, and pretty high confidence proteomic statistics). Of course, we also agree that we cannot claim that all are true positives. For this reason we introduced the table of the 62 common hyperphosphorylated peptides and we have soften the conclusions.

Often, proteomics studies are performed using just one of the phosphoenrichment methods using biological duplicates or triplicates. However, it has been reported that the different phosphopeptide enrichment methods isolated distinct, partially overlapping segments of a phosphoproteome, whereas none of the methods were able to provide a whole phosphoproteome (Zhou et al 2001, Bodenmiller et al 2007). Enrichment techniques are complementary, such that a combination of methods greatly enhances the number of phosphopeptides isolated from complex samples (Dunn et al 2009). So, none of the phosphopeptide enrichment methods provide a whole phosphoproteome. Each method provides

varying degrees of selectivity and specificity of phosphopeptide enrichment resulting in the identification of subsets of phosphopeptides of different nature. Therefore, our idea was to use the different phosphopeptide enrichment methods in order to identify the highest spectrum of the PP2A-Cdc55 phosphoproteome. Actually, we really think the strength of our data is indeed based on the 3 different enrichment methods.

To properly assess PP2A-Cdc55 substrates the ideal experiment would be to analyse the kinetics of dephosphorylation for the wt and deleted strains as they exit mitosis, similar to previous studies (Bouchoux and Uhlmann, 2011; Cundell et al., 2016). This may also explain why Mob1, which the authors previously showed to be a PP2A substrate, did not show up as a strong and significant hit in there SILAC approach.

In our experimental conditions, we can detect increase phosphorylation of PP2A-Cdc55 substrates already in metaphase; and in most of the cases since the protein is hyperphosphorylation in absence of Cdc55, its phosphorylation does not increase more during anaphase. Of course, we cannot rule out that it might occurs for some specific substrates, but in general in absence of Cdc55 the substrates are equally hyperphosphorylated in metaphase and in anaphase (see Bfa1 and Mob1 in Baro et al 2013, Figure 1 and Figure 2).
Mob1 was identified as medium confidence phosphopeptide with increase phosphorylation in absence of Cdc55, already in the first SILAC-Phosphoproteome analysis that we performed (Method 1). In fact, thanks to this identification we proceed to study the physiological relevance of this substrate and we already published it (Baro et al 2013). At that moment, we looked for protein in “our favourite” candidates list (proteins related to FEAR and MEN pathways), not only in the high confidence peptides but also in the medium confidence ones. However, for a global analysis of the data as we are doing here, we can only include high confidence peptides.

Also without the kinetic data, it is difficult to deconvolve the direct and indirect effects of PP2A deletion. It's possible that many of the increased phosphorylation events could be due to disruption of negative and positive feedback loops that impinge on other phosphatases and kinases (Grallert et al., 2014).
It is also unclear exactly how 'increased' phosphopeptides were chosed and how these were subsequently analysed and compared especially with regards to the precise number of unique phosphosites that are specifically increased between PP2A wt and deletion strains.

We are really sorry since we noticed that we have failed to properly explain how we made the phosphopeptide identification. We have amended the manuscript in several sections and introduce further explanations in Results and Materials sections.
Combining the three methods, we identified 1260 high confidence phosphopeptides with increased phosphorylation (Heavy (wt)/light (cdc55mutant) ratio < 0.75) in the absence of Cdc55 phosphatase; therefore likely to be PP2A-Cdc55 substrates.
We agree that we cannot distinguish among direct and indirect effects on PP2A-Cdc55 deletion, and in fact we already discussed that point in the manuscript (Results and discussion). Nevertheless, during anaphase a key mitotic phosphatase in budding yeast, Cdc14 is activated, and in order to exit from mitosis most of the phosphorylation events are erased, mitotic kinases (at least Cdk1 and Polo-kinase) are inactivated, therefore we believe that the feedback loops among phosphatases and kinases will be higher during anaphase. This consideration and the fact that the system of cdc55 deletion in metaphase

arrested cells worked pretty well in our laboratory, prompted us to use this set-up.

Despite these concerns, there is still some interesting data which could form the basis of an interesting publication. The authors should take the above limitations into account, and alter their conclusions accordingly. They would also benefit to rethink exactly what the experiment they have performed is actually telling them, and perhaps consider revising the initial goal to better suit the experimental data.

We thank the reviewer for his/her global evaluation of the manuscript. We think we have amended most of his/her concerns.

We are really sorry since we noticed that we failed to properly explain how the analysis was performed. In the new version of the manuscript, we have explained better how the data was acquired and analyzed (results and methods sections) and now we provide better statistical parameters details of the phosphopeptides.

Combining the three methods, we identified 1260 high confidence phosphopeptides with increased phosphorylation (Heavy (wt)/light (cdc55mutant) ratio < 0.75) in the absence of Cdc55 phosphatase; therefore likely to be PP2A-Cdc55 substrates. Those 1260 hyperphosphorylated peptides include 62 phosphopeptides that were identified in at least 2 (out of 3) enrichment methods (new Figure 1I) with a t-test p-value <0.05. Therefore, considering the different nature of the phosphopeptides identified for each method, the 62 phosphopeptide are pretty likely to be PP2A-Cdc55 substrates. Nevertheless, we cannot consider the remaining 1198 hyperphosphorylated peptides to be all false positives (some phosphopeptides were identified with highly amounts of PSM, and pretty high confidence proteomic statistics). Of course, we also agree that we cannot claim that all are true positives.

Specific points:

1. The introduction is a bit long and could be shortened, also suggest moving the discussion about previous mass spec studies to the discussion.

Done. We have shorten the introduction and move previous mass spec studies to the discussion section.

2. Page 4 Line 87: Perhaps consider citing either the Hunt or Castro labs for the role of Greatwall in regulating PP2A. (see 2010 Science papers).
Done

3. Page5 Lin105-7: "PP2ACdc55 downregulation in anaphase": I assume the authors mean increased activity here as PP2A is reactivated during mitotic exit not repressed.

As we previously published, budding yeast PP2A-Cdc55 is downregulated during anaphase (Queralt et al 2006). The reactivation of the PP2A-Cdc55 occurs upon exit from mitosis, during cytokinesis/G1.

4. Figure 1A: It would be nice to have a diagram here where you show the work flow. Specifically highlighting the Wt and deleted strains being compared, which one was grown in which media (Heavy or Light), number of biological replicates etc .as I think this may help readers understand what is being

compared. You could condense and simplify additional figure 1 and insert it here perhaps?
Done

5. Page 8 Line 181: Some greater clarification about the number of phosphopeptides that are increased in the deletion mutant are needed. From Figure 1H it appears that there are only 27 sites. But the text suggest that this number is 1,260. I assume that 1A is the whole dataset, that is 1260 sites were identified across both the wt and del mutant? If so Fig1H is the most important figure and the analysis needs to be primarily performed on the 26 sites that are increased compared to the wt. Not the complete 1260.

We have explain better this point in Page 8, Results section. We were able to quantify globally 1491 phosphoproteins, represented by 4467 phosphopeptides. Among them, we found 1,260 hyperphosphorylated peptides in the cdc55 mutant which show H/L ratios <0.75 (log2(H/L)<-0.42), corresponding to 628 phosphoproteins. The 1260 phosphopeptides (or 628 phosphoproteins) have increase phosphorylation in absence of Cdc55.
Those 1260 hyperphosphorylated peptides include 62 (increased) phosphopeptides that were identified in at least 2 (out of 3) enrichment methods (new Figure 1I) with a t-test p-value <0.05. Therefore, considering the different nature of the phosphopeptides identified for each method, the 62 phosphopeptide are considered hyperphosphorylated peptides, which showed similar performance in the different purification protocols used, rather than being more likely to be PP2ACdc55 substrates. Indeed, each approach uncovered a unique subset of hyperphosphorylated peptides useful for downstream analysis (some hyperphosphorylated peptides found in only 1 method were detected with a huge amount of PSM and great scores; therefore, those are also pretty likely to be substrates).

6. Additional File 2: What are the values listed? Are these are log2 H/L ratios as they are all below 1? A full table with the H/L ratios for all identified phosphopeptides along with the p-value would be very helpful. In addition, there should be a comparisons between biological replicates along with p-values to ensure that sites identified are significant. Were label-swaps performed? This data should be presented in a single clearly labelled table.
Additional file 3 (old Additional file 2) has been improved following the reviewers considerations.

7. It appears the data has been heavily averaged, and I couldn't quite follow what was done in order to generate Figure 1A. Why was a H/L ratio of less than 0.75 considered to be increased phosphorylation? Normally a ratio greater than would be the desired cut-off would be used (e.g. >1 for a 2-fold increase). Similarly, a value <-1 would be considered significantly decreased with values between -1 and +1 considered unchanged. These need to be clarified.

The first point to clarify is that the phosphopeptides of interest are the ones with H/L ratio < 0.75 or log2(H/L) ratio < -0.42. In the material and methods (section: "Stable Isotope Labelling of Yeast Cells and Preparation of Yeast Protein Extracts for Phosphoproteomic Analysis") we specify that the wild type (Y858) and the cdc55Δ (Y859) strains were labeled with the heavy (H) and light (L) aminoacids, respectively (we hope this point will be now much clear with the new Fig. 1A). Therefore, what is expected for the cdc55 susbtrates is the H/L ratio below 1 (or 0 in log scale). We set our threshold to 0.75 (-0.42 in log scale) as commonly done in the Proteomic laboratories for similar set-ups.
In the figure 1A (new Figure 1B) we show the whole dataset which is the result of combining the three experimental approaches (TiSH, SIMAC and TiO2) and applying the selection criteria described in the last

paragraph of the “Data Analysis for Peptide Identification and Quantitation” section in material and methods. This gives us 4,467 phosphosites being 1,260 of them the hyperphosphorylated ones (H/L ratio below 0.75, in linear scale). Moreover, the figure 1H (new Figure 1I), indeed show the statistically significant phosphopeptides which are included in at least 2 out of 3 experimental conditions (TiSH, SIMAC and TiO2). Among them, 62 are the ones that are significantly hyperphosphorylated in absence of cdc55 in more than one approach. We have modified both figures (new Figure 1B and Figure 1I) for clarity.

8. From Figure 1H it appears that only 27 or 62 phosphosites are increased in the deleted vs wt strains. If this is correct, then the analysis should be performed on these 27/62 phosphosites, not the full dataset. This goes for the rest of the analysis, such as the STRING, motif-x and GO-enrichments. This also seems to contradict Figure1A, can the authors explain this better?

The figure 1A (new Figure 1B) was generated averaging all the single phosphopeptides obtained in the three experimental approaches (we considered the three approaches as biological replicates). The total number of phosphosites ploted were 4,467 being 1,260 of them hyperphosphorilated (H/L<0.75) in absence of Cdc55. On the other hand, as we described above, the wild type cells were heavily labeled while the cdc55 KO cells were labeled with light aminoacids. Therefore, in a H/L ratio the phosphopeptides with a ratio below 1 (0 in log scale) are those that are hyperphosphorylated. We set the significance ratio below 0.75 (-0.42 in log scale) for hyperphosphorylated peptides.
We decided to use all the hyperphosphorylated peptides to increase the statistical power of the systems biology analysis (motif-x, GO analysis and STRING). This is an exploratory analysis without the aim to fully characterize the specificity of Cdc55. While we agree that the 1260 hyperphosphorylated peptides might include false positives, the bioinformatics studies allow us to uncover some interesting features:

1. We identified 10 sequences motif susceptible (MotifX) to be recognized by PP2A-Cdc55, while so far only one was properly described (S/TP). This finding is in agreement to previous published PP2A-Cdc55 substrates that do not contain the S/T P site.
2. String analysis identified two nodules, Cdc28 and Cla4, as putative kinases to be counteracted by PP2A-Cdc55. It has been pretty well established in the field that PP2A-Cdc55 is a phosphatase counteracting Cdc28 kinase at the S/T P sites. PP2A-Cdc55 was modestly related to Cla4 in the literature, and we suggest that they might be really functionally linked.
3. The GO suggested some biological processed to be regulated by PP2A-Cdc55. Several published PP2A-Cdc55 functions are included in those GO, confirming previous data. New biological processes like cytokinesis is not surprising due to the cdc55 deletion mutant phenotype and the described localization of PP2A-Cdc55 to the cytokinesis locus. However, its function and targets during cytokinesis in budding yeast have not been examined. We are currently studying the PP2A-Cdc55 function in cytokinesis in our laboratory in detail.

We think that those bioinformatics analysis mostly confirm some published data and we carefully suggested new features of the PP2A-Cdc55 focusing on those with previously reported data.

9. Page 10 Line 233: 562 unique motifs seem very high especially for an input of 721 peptides, it's almost 1:1, and above the number of total kinases in yeast. Also, the authors should clarify exactly which phosphopeptides were used for the analysis. Again if they want to show PP2A specific effects then only

the phosphopeptides that are specifically increased in the deletion vs wt should be analysed.

We thank the reviewer to point this out. There is a missunderstanding here and we have amended the text in the Results sections. As shown in figure 2A, there is only 10 unique phosphomotifs (8 for serines and 2 for Threonines).
When we mentioned before 562 unique motifs we meant 562 unique aa sequences around the phosphorylated residue. This have been fixed in the text.

10. Page 11: "Novel roles for PP2ACc55 phosphatase in cytokinesis and endocytosis". A 'd' is missing in the title. Also, I think a bit too much is made of the GO-terms. These are most likely reflecting the fact that you have mitotically arrested samples and you cannot conclude that they are PP2A specific, without doing additional assays. It is also not clear what is being compared. The most logical thing here would be to look for differences between the wt and deleted strains, but this doesn't appear to be what was done. Rather, all phosphopeptides identified were analysed. If this is the case, then the analysis is likely only reflecting the mitotic state, and not able to specifically tell you anything about PP2A substrates.

The 1260 hyperphosphorylated peptides are the ones increase in the cdc55 mutant, therefore the analysis is done looking for the differences between the wt and the cdc55 delete strains.
We agree that the use of the whole list of hyperphosphorylated proteins (628 proteins from 1,260 phosphopeptides) is a handicap. However, the 62 phosphopetides (55 proteins) identified in more than one approach reflect more the nature of those phosphopeptides to be enrich using different columns, more than to be more likely to be real substrates. Indeed, each approach uncovered a unique subset of hyperphosphorylated peptides useful for downstream analysis (some hyperphosphorylated peptides found in only 1 method were detected with a huge amount of PSM and great scores; therefore, those are also pretty likely to be substrates).

In addition, 55 proteins is a low number in statistical terms, therefore, an analysis only with 62 phosphopeptides will generate more doubts than real questions. Use the whole list, by contrast, is highly confident in terms of statistics and give us a promising starting point in the understanding of the biological meaning of the peptides that appear hyperphosphorylated when cdc55 is knocked-down. In any case, as mentioned above, the systems biology analysis (GO terms, networks and motif-x) is something exploratory focused in opening doors more than answering questions.
The GO analysis is a bioinformatic analysis which main goal is to characterize the Gene Ontology terms specifically enriched in a population of genes/proteins when they are compared with the whole genome. Therefore, the biological conclusions must always be taken carefully. In our work we analyzed the Gene Ontology terms enrichment in a set of proteins carrying hyperphosphorylated peptides in a cdc55 knock-down mutant. We agree that our results likely mean that the mitotic state of the yeast is altered. However, it also true that the population of phosphoproteins used in the comparison is deducted from a list of peptides that appeared hyperphosphorylated when cdc55 was knocked-down. Consequently, it seems highly probable that the enrichement of certain functionalities is due to the absence of this protein in the cell. In fact, the GO suggested some biological processed to be regulated by PP2A-Cdc55. Several published PP2A-Cdc55 functions are included in those GO, confirming previous data. New biological processes like cytokinesis is not surprising due to the cdc55 deletion mutant phenotype and the described localization of PP2A-Cdc55 to the cytokinesis locus, but was not described/published for Cdc55 in budding yeast. We are currently studying the PP2A-Cdc55 function in cytokinesis in our laboratory in detail.

11. Figure 4: A non-PP2A substrate should be included to show that any disruption to the timing of mitotic exit potentially caused by PP2A-Cdc55 deletion is not affecting dephosphorylation kinetics. Also timing here would be more appropriate label rather than cell cycle stage, which is subjective. Would also consider aligning all of the blots above and below each other. The phosphatase assay should be labelled as a separate panel (e.g. 4C), and a loading control is needed.

We have included Pgk1 as the loading control. The timing of the experiments is a bit different every day we release the cells from the metaphase arrest. Therefore, we estimate anaphase spindles by immunofluorescence, look for Clb2 protein levels and budding index and identify the cell cycle stages combining all these parameters.
We prefer to do not separate the phosphatase assay in another panel since we run all the samples together in the same protein gel. To separate them in two panels we will have to repeat some lanes in two different panels and it will look odd.
A complete demonstration that timing of mitotic exit and that the dephosphorylation kinetics are not disturb in a cdc55 deletion strains was already published from our lab (Baro et al 2013, Figure 3). In this figure we analyzed mitotic progression by Cdh1 dephosphorylation, Clb2 phosphorylation and degradation, Cdc5 degradation and accumulation of Sic1. As already demonstrated in the paper, the timing and general features of mitotic exit are similar in the wt and cdc55 deleted strain.

References:
Bouchoux, C., Uhlmann, F., 2011. A Quantitative Model for Ordered Cdk Substrate Dephosphorylation during Mitotic Exit. Cell 147, 803-814. doi:10.1016/j.cell.2011.09.047
Cundell, M.J., Hutter, L.H., Bastos, R.N., Poser, E., Holder, J., Mohammed, S., Novák, B., Barr, F.A., 2016. A PP2A-B55 recognition signal controls substrate dephosphorylation kinetics during mitotic exit. J. Cell Biol. 257, jcb.201606033-554. doi:10.1083/jcb.201606033
Grallert, A., Boke, E., Hagting, A., Hodgson, B., Connolly, Y., Griffiths, J.R., Smith, D.L., Pines, J., Hagan, I.M., 2014. A PP1-PP2A phosphatase relay controls mitotic progression. Nature 517, 94-98. doi:10.1038/nature14019
Olsen, J.V., Vermeulen, M., Santamaria, A., Kumar, C., Miller, M.L., Jensen, L.J., Gnad, F., Cox, J., Jensen, T.S., Nigg, E.A., Brunak, S., Mann, M., 2010. Quantitative phosphoproteomics reveals widespread full phosphorylation site occupancy during mitosis. Sci Signal 3, ra3-ra3. doi:10.1126/scisignal.2000475

Reviewer #2: The authors report on the identification of new substrates for PP2A Cdc55 phosphatase during mitosis using mass spectrometry by comparing the phosphoproteome of a wild type yeast strain to that for a cdc55 lacking mutant strain. The manuscript is well-written and appears comprehensive in reporting how the current literature relates to their results on PP2A Cdc55 phosphatase substrates.

Since I only have a layman's knowledge of proteomics and cell cycle signalling pathways, the main aim of this review is to check the consistency of the data supporting the results reported in the manuscript by the authors. In general, I feel Baro et al have been comprehensive in disseminating the data associated with their manuscript and I have only minor suggestions for improvements.

The manuscript requires an, "Availability of supporting data and materials" section. In this section, information about the raw data stored in PRIDE should be provided.
Done

With regards to the data itself, the dataset contains *.raw files which have been generated by the Orbitrap mass spectrometer. The dataset also contains *.msf files - what are these?

The msf file is the report generated by the Proteome Discoverer Software and include a compilation of the different technical replicates in one unique report. It includes the search in databases (filtered by Percolator algorithm using a FDR < 1%), the normalized quantification values of the features (peptides) as well as the list of the proteins. This file can be opened with the Proteome Discoverer software (and other proteomic software) so that the user can easily visualize the data and use different settings for the filters.

I think it would be helpful if the dataset provides information on the files it contains such as what they are and which experiments they correspond to. For example, are the files from the large-scale identification of PP2A Cdc55 -dependent phosphoproteome work, TAP- or HA-tagging experiments? Is it possible to add this information into their PRIDE archive dataset entry webpage or in a README text file that is added to their dataset?
Done

Large-scale identification of PP2ACdc55-dependent phosphoproteome in metaphase-arrested cells

Can the authors check that Fig. 1C is referred to in the manuscript text?

We thank the reviewer for this point. We missed to referred to Fig.1C in the previous version of the manuscript and it has been fixed now (new Figure 1D).

The list of peptides listed in PDF files in Additional files 3 and 4 could be provided in an Excel file or text format. This will aid the re-use of the data instead of users having to copy and paste or even type out them manually if the copying function does not work properly in their PDF document applications.

Done. We extended the reviewers comments to all figures, and all the Additional file containing list of elements presented before as pdf have been changed to xlsx.

Novel roles for PP2A Cdc55 phosphatase in cytokinesis and endocytosis

Are the protein interactions of the network shown in Additional file 7 available as a text file as well as a screenshot? This would be more useful if people wanted to re-use it.

We have added a complete list of the protein interactions nodes in the new Additional file 10.

Validation of novel PP2A cdc55 substrates in mitosis

Page 15 first paragraph: Is the reference to Additional file 1 correct on line 337? Maybe Additional file 3 is the correct file to be referred to? If so, where is Kin4 in this file?

Again we thank the reviewer to point this mistake. It should be new Additonal file 3.

Data Analysis for peptide identification and quantification

This Section mentions the use of R with the rvest, vennerable, venneuler packages - page 27 line 640. The R scripts should be provided along with documentation on how to use it. Since R scripts are source code then the manuscript will need an, "Availability of source code and requirements" section as detailed in the authors instructions (https://academic.oup.com/gigascience//pages/research).

Done. Included in Additional file 15.

Structure prediction of Cdc55

I do not have experience of the tools used in this section. Are HHpred and MODELLER command line tools? If so, then any scripts involving their use should be make available.

HHpred, MODELLER and HADDOCK are software or/and online tools available (websites) to perform this kind of analysis.

Sampling the binding interface of the CDC55/Mob1 complex
If HADDOCK is a command line tool then any script involving its use should be made available.

--

Peter Li, GigaScience

Reviewer #3: Baro et al conducted an analysis of PP2A-Cdc55 substrates and interacting proteins under mitotic arrest conditions in budding yeast by proteomics and quantitative phosphoproteomics. They validated differences in phosphorylation for proteins involved in the MEN and FEAR pathway as well as a different PP2A regulatory subunit, and generated a docking model for Cdc55 and Mob1.
PP2A is a phosphoprotein phosphatase consisting of catalytic, scaffolding, and regulatory subunit. The regulatory subunit confers substrate specificity. The PP2A regulatory subunit Cdc55 is the yeast homolog of B55 in humans and thought to be responsible for counteracting Cdk1 phosphorylation during mitotic exit. In yeast, PP2A-Cdc55 acts in concert with the Cdc14 phosphatase during mitotic exit.

The authors indicate that while their study was under preparation, another study was published that conducted PP2A-Cdc55 substrate analyses in G1, S, and G2. A comparison of the datasets should be included.

We have compared of our dataset with Godfrey et al. We have found 69% (128 common/186 proteins in Godfrey et al) of the proteins identified in Godfrey et al indicating a high degree of overlapping in both studies. We have discussed it in the discussion section and included the common proteins in Additional file 13.

Baro et al uses a Cdc55-delete strain to identify potential substrates of PP2A-Cdc55 in mitotic arrest which is induced by Cdc20 depletion. The authors state that 95% of cells were arrested in metaphase but do not show data to support this. This should be included.

The metaphase arrest in a cdc55 deletion strains is a well-established system in our laboratory leading to a 95-100% arrest of the cells in metaphase. The characterization of the mutant and mitotic exit kinetics were carefully studied in our laboratory and published previously (Baro et al 2013).

To detect differences in phosphorylation between wild-type and Cdc55-delete strains, the authors use a SLIAC strategy. It would be helpful if the authors could include a scheme of the experimental design and clearly indicate which strain was labeled heavy and which strain was labeled light.

Done. Included in new Figure 1A.

Interestingly, the authors used three different strategies for phosphopeptide enrichment but do not provide an explanation for this unusual combination. Please explain.

We have included the explanation in the manuscript in Results section.
Often, proteomics studies are performed using just one of the phosphoenrichment methods using biological duplicates or triplicates. However, it has been reported that the different phosphopeptide enrichment methods isolated distinct, partially overlapping segments of a phosphoproteome, whereas none of the methods were able to provide a whole phosphoproteome (Zhou et al 2001, Bodenmiller et al 2007). Enrichment techniques are complementary, such that a combination of methods greatly enhances the number of phosphopeptides isolated from complex samples (Dunn et al 2009).
In addition, the TiSH method (a combination of enrichment and fractionation methods abbreviated TiSH for TiO2-SIMAC-HILIC) has some others peculiarities: extensive HILIC fractionation of the monophosphorylated peptides increases the phosphopeptide coverage (Engholm-Keller et al 2012).
Moreover, the ratio of monophosphorylated and multiphosphorylated peptides detected for each method also varies considerably:
Mono-phosphorylated/multiphoshorylated (in our dataset)
42%/58% TiO_2
83%/17% SIMAC
90%/10% TiSH

So, none of the phosphopeptide enrichment methods provide a whole phosphoproteome. Therefore, our idea was to use the different phosphopeptide enrichment methods in order to identify the biggest PP2A-Cdc55 phosphoproteome.
The set-up of our experiment was to use the three different phosphoenrichment methods available at the moment using SILAC. We performed a biological replicate for each method with technical duplicates or triplicates (triplicates for SIMAC and duplicates for TiO_2 and TiSH). Due to the intrinsic and distinct nature of the phosphoenrichment methods, we do not expect to increase the reproducibility of the three different phosphopeptide enrichment methods and the overlapping will remain low. To perform the sample data analysis we used the raw files from the technical replicas of each experimental method (described above). A unique peptide and protein list was obtained from the compilation of all the replicates' raw files for each phosphoenrichment method used. We have explained those points better in the manuscript.

A table of all identified peptides or at least phosphopeptides is not included, please do so.

The list of hyperphosphorylated peptides is included in new Additional file 3. The whole set of peptides identifed in the wt and cdc55 strains are deposited in PRIDE as msf.files. A summary can be found in new Additional file 2 containing 10069 peptides.

In their analyses, they identify 1,260 phosphorylation sites that increase in phosphorylation upon Cdc55 deletion. While a table of hyperphosphorylated sites is included, the description of this table (additional file 2) needs to be improved to be understandable for the reader. Are the values in the TiSh, SIMAC, TiO2 column ratios? If so of what? The statistical analysis is missing. It would also be helpful if either the site of phosphorylation would be indicated in the peptide sequence in column c or if the peptide sequence would be represent as centered around the phosphorylated residue +/- 6 amino acid for instance. P-values for the quantification of each phosphopeptides should be included.

Done. New Additional file 3 (old Additional file 2)has been improved following the reviewers suggestions.

Only the hyperphosphorylated peptides are included in additional file 2, however, the volcano plot in Figure 1H shows all data. This data needs to be added.

The volcano plot includes the 1260 hyperphosphorylated peptides in absence of Cdc55 and not all the peptides identified. We have modified the volcano plot for clarity (new Figure 1I).

To exclude that differences detected in phosphorylation are due to do difference in protein abundance, the authors conduct an analysis of protein abundance differences in both strains. However, the authors do not conduct a subtractive analysis to correct phosphopeptide ratios by protein abundance differences, just state that most proteins don't change. Such an analysis should be included.

As the reviewer suggested, correct phosphopeptide ratios by protein abundance differences will be the perfect escenario. For that you need to be able to detect “ALL” the protein and normalize by the their abundance. But the reality in proteomics is that only a fraction of the protein identified in our enrichment fractions is also identified in the whole extract (without enrichment). We have separate the whole cell extract in 8-10 fractions in order to increase the chances to identify more proteins, but still not all the proteins are detected. Therefore, the analysis we show is the best we can do at this point with the limitations of the proteomics. For instance, we were able to detect 45 proteins out of the 55 (corresponding to 62 phosphopeptides) hyperphosphorylated proteins (new figure 1J); and as included in the manuscript most of them do not change their protein abundance.
In addition, after several years of working with cdc55 mutant, we did not observe global changes in protein abundance in the cdc55 mutant. Moreover, all the proteins we have deeply studied previously do not change protein levels especifically in a cdc55 mutant (Queralt et al 2006, Queralt and Uhlmann 2008, Baro et al 2013).

The identification and quantification results for the protein analysis are not included. Only a list of protein names is provided in additional file 3. The complete list of all quantified protein ratios and their

identification metrics needs to be added.

Done. Included as new Additional file 4.

Only 145 sites are identified in 2 out of 3 replicates and only 30 in all three replicates. The low reproducibility of identifications and quantifications is potentially due to the use of three different enrichment methods, each of which likely selects for a different subset of phosphopeptides. Because the overlap of the three or even two out of three datasets is very low, subsequent analyses based on phosphopeptides that are identified as statistically significantly increased are limited. To improve the dataset, the authors need to improve the overlap of the phosphoproteomic analyses for instance by using the same phosphopeptide enrichment strategy in replicate to ensure greater reproducibility in phosphopeptide identification and quantification.

As the reviewer noted, none of the phosphopeptide enrichment methods provide a whole phosphoproteome and they purified peptides of different nature. Each method provides varying degrees of selectivity and specificity of phosphopeptide enrichment. Therefore our idea was to use the different phosphopeptides enrichment methods in order to identify the biggest PP2A-Cdc55 phosphoproteome. We actually think, that the strength of our data is indeed based on the 3 different enrichment methods. Due to the intrinsic and distinct nature of the phosphoenrichment methods, we do not expect to increase the overlapping of the 3 different enrichment methods increasing the amount of biological replicates. To perform the sample data analysis we used the raw files from the technical replicas of each experimental method (described above). A unique peptide and protein list was obtained from the compilation of all the technical replicates' raw files for each phosphoenrichment method used. We have now clarified all these points in the manuscript.
We are unable to provide biological triplicates of each phosphoenrichment method. Currently, the three proteomics units are using different and new mass-spectrometers; therefore is technically impossible to performed biological triplicates in the same conditions as we did several years ago.

Instead of concentrating on the 62 phosphopeptides with statistically significant increases in phosphorylation, the authors conduct their subsequent analyses on all increased phosphorylation sites. Because the abundance change of these phosphopeptides is not supported by statistical analysis, these analyses are not informative. For instance, the authors state that they find a preference of PP2A-Cdc55 for the dephosphorylation of phosphothreonine over phosphoserines as reported by others. However, the enrichment in threonine-directed phosphorylation sites is not observed in just the 62 statistically significant increased phosphorylation sites. The same strategy of using all increased sites is applied for all subsequent analysis, including assigning counteracting kinases, GO and String network analysis, and needs to be revised using a dataset with a higher degree of overlap in phosphopeptide identifications and quantifications.

Just note that the 62 phosphopeptides of the volcano plot are not the most statistically significant ones. They reflect the phosphopeptides being enrich in more than one approach due to their intrinsic nature. Considering the different nature of the phosphopeptides identified for each method, the 62 phosphopeptide are pretty likely to be PP2A-Cdc55 substrates. Nevertheless, we cannot consider the remaining 1198 hyperphosphorylated peptides to be all false positives (some phosphopeptides were identified with highly amounts of PSM, and pretty high confidence proteomic statistics). Of course, we

agree that we cannot claim that all are true positives.

We agree that the use of the whole list of hyperphosphorylated proteins (628 proteins from 1,260 phosphopeptides) instead of the repeated ones is a handicap. However, this number is low in statistical terms, therefore, an analysis only with 55 proteins will generate more doubts than real questions. Use the whole list, by contrast, is highly confident in terms of statistics and give us a promising starting point in the understanding of the biological meaning of the peptides that appear hyperphosphorylated when cdc55 is knocked-down. In any case, as we mentioned above, the systems biology analysis (GO terms, networks and motif-x) is something exploratory focused in opening doors more than answering questions.
The GO analysis is a bioinformatic analysis which main goal is to characterize the Gene Ontology terms specifically enriched in a population of genes/proteins when they are compared with the whole genome. Therefore, the biological conclusions must always be taken carefully. In our work we analyze the Gene Ontology terms enrichment in a set of proteins carrying hyperphosphorylated peptides in a cdc55 knock-down system. We agree that our results likely mean that the mitotic state of the yeast is altered. However, it also true that the population of phosphoproteins used in the comparison is deducted from a list of peptides that appeared hyperphosphorylated when cdc55 was knocked-down. Consequently, it seems highly probable that the enrichement of certain functionalities is due to the absence of this protein in the cell. In fact, the GO suggested some biological processed to be regulated by PP2A-Cdc55. Several published PP2A-Cdc55 functions are included in those GO, confirming previous data. New biological processes like cytokinesis is not surprising due to the cdc55 deletion mutant phenotype and the described localization of PP2A-Cdc55 to the cytokinesis locus, but was not described/published for Cdc55. We are currently studying the PP2A-Cdc55 function in cytokinesis in our laboratory in detail.

The authors also perform Cdc55 interactome studies. Again only protein names are provided in additional file 8, no information on peptide counts versus controls or parameters/metrics of identification are provided. Surprisingly, for the analysis of HA-tagged Cdc55 this information is available in additional file 9. Here the authors perform two replicates, however, it is not determined if the identifications were reproducible. There is no explanation why the authors performed TAP as well as HA purifications. The authors concluded that the interactomes analysis in total are not informative because only a few proteins identified in the phosphoprotomics analysis are captured here.

The purification of Cdc55 was an alternative strategy that we use in our laboratory in order to identify processes related to PP2A-Cdc55. We tried two purification strategies (HA and TAP) that were optimized in our laboratory for other purposes. We agree that some of the rawdata is pretty por (HA purification), but sadly is what we get at that moment from the Proteomic service that perform the analysis. For the TAP strategy, the proteomic service provide us with the raw data (Excel file) and it is included in the PRIDE repository.

Finally, the authors perform an in silico analysis of Cdc55 and Mob1 to identify interaction surfaces which recapitulates a previously identified interface on Cdc55 but does not identify a corresponding interface on Mob1. In general the identification of PP2A holoenzyme specific substrates is of high interest and could be a great resource for the community. However, the analyses conducted here lack rigor and need to be improved upon to be valuable.

We thank the reviewer for his/her insights and we trully believe that we have addresses most of his/her concerns in the new version of the manuscript.

We are really sorry since we noticed that we failed to properly explain why we used the three different phosphoenrichment methods. In the new version of the manuscript, we have explained better why we used the three phosphoenrichment methods and we provided better statistical parameters details of the phosphopeptides.

Combining the three methods, we identified 1260 high confidence phosphopeptides with increased phosphorylation (Heavy (wt)/light (cdc55mutant) ratio < 0.75) in the absence of Cdc55 phosphatase; therefore likely to be PP2A-Cdc55 substrates. Those 1260 hyperphosphorylated peptides include 62 phosphopeptides that were identified in at least 2 (out of 3) enrichment methods (Figure 1H) with a t-test p-value <0.05. Therefore, considering the different nature of the phosphopeptides identified for each method, the 62 phosphopeptide are pretty likely to be PP2A-Cdc55 substrates. Nevertheless, we cannot consider the remaining 1198 hyperphosphorylated peptides to be all false positives (some phosphopeptides were identified with highly amounts of PSM, and pretty high confidence proteomic statistics). Of course, we also agree that we cannot claim that all are true positives.

Often, proteomics studies are performed using just one of the phosphoenrichment methods using biological duplicates or triplicates. However, it has been reported that the different phosphopeptide enrichment methods isolated distinct, partially overlapping segments of a phosphoproteome, whereas none of the methods were able to provide a whole phosphoproteome (Zhou et al 2001, Bodenmiller et al 2007). Enrichment techniques are complementary, such that a combination of methods greatly enhances the number of phosphopeptides isolated from complex samples (Dunn et al 2009). In addition, the TiSH method (a combination of enrichment and fractionation methods abbreviated TiSH for TiO2-SIMAC-HILIC) has some others peculiarities: extensive HILIC fractionation of the monophosphorylated peptides increases the phosphopeptide coverage (Engholm-Keller et al 2012). Moreover, the ratio of monophosphorylated and multiphosphorylated peptides detected for each method also varies considerably:
Mono-phosphorylated/multiphoshorylated (in our dataset)
42%/58% TiO2
83%/17% SIMAC
90%/10% TiSH

So, none of the phosphopeptide enrichment methods provide a whole phosphoproteome. Each method provides varying degrees of selectivity and specificity of phosphopeptide enrichment resulting in the identification of subsets of phosphopeptides of different nature. Therefore our idea was to use the different phosphopeptide enrichment methods in order to identify the highest spectrum of the PP2A-Cdc55 phosphoproteome. Preliminar results with one biological replicates in one of the approaches did not increase the reproducibility of the three different phosphopeptides enrichment methods. The intrinsic reproducibility of a given phosphoenrichment methods is high, but do not increase the matching phosphopeptides in more than one approach. Due to the intrinsic and distinct nature of the phosphoenrichment methods, we do not expect to increase the reproducibility of the three different phosphopeptide enrichment methods and the overlapping will remain low. Actually, we really think the strength of our data is indeed based on the 3 different enrichment methods.

Close
