# Supplementary material for: SILAC-based phosphoproteomics reveals new PP2A-Cdc55-regulated processes in budding yeast
Source: Gigascience. 2018 May 24;7(5):giy047. doi: 10.1093/gigascience/giy047 (PMC5967524; doi:10.1093/gigascience/giy047)
Supplement: Response_to_Reviewer_Comments_Revision_1.pdf [file giy047_response_to_reviewer_comments_revision_1.pdf]

Author's Response To Reviewer Comments

Close

Response to Reviewers:

Reviewer #1
Please "increase the font size on many of their figures, the labelling is very small and difficult to read (most Figure 1 especially)."

We thank the reviewer for his global evaluation of the manuscript. We thank the reviewer to suggest to increase the font size. Indeed, some labeling was very small. We have increased the font size of the figures 1, 2 and 3 following the reviewer suggestions.

Reviewer #2
"The R scripts in Additional file 15.doc should all be uploaded into a GitHub (https://github.com) repository with documentation on their execution if this manuscript is accepted for publication. The technical team at GigaScience can assist with this if required."

Following the reviewer suggestions we will upload the R scripts to the GigaScience GitHub. We have tried to do it ourselves and we did not manage; therefore, we will need assistance from the GigaScience technical team to do it.

Reviewer #3:
The main concern about lack of experimental rigor with regards to phosphopeptide quantifications is still not resolved. This concern is shared with another reviewer, and the authors did not address it in revision. Because of that, the conclusions drawn from this dataset in the systems biology analyses are questionable. The comment: "we cannot consider the remaining 1198 hyperphosphorylated peptides to be all false positives (some phosphopeptides were identified with highly amounts of PSM, and pretty high confidence proteomic statistics)" does not address the problem of reproducible quantification, just identification. Inclusion of all sites in the downstream analysis is not supported by the data and leads to confusion. The authors' statements: "We decided to use all the hyperphosphorylated peptides to increase the statistical power of the systems biology analysis (motif-x, GO analysis and STRING)" and "In addition, 55 proteins is a low number in statistical
terms, therefore, an analysis only with 62 phosphopeptides will generate more doubts than real questions" are difficult to reconcile with each other, and therefore problematic. Including more poor data is not the correct approach to increase statistical power.

We thank the reviewer for her global evaluation of the manuscript. Following the reviewer suggestion we have performed the analyses with the 62 statistically significant phosphopeptides (Fig. 1I). In addition, all the manuscript text has been changed accordingly. All the changes are listed below:

1. New Fig. 1D. The Ser/Thr distribution was calculated from the 62 phosphopeptides.
2. New Fig. 1E. The SP/TP sites distribution was calculated from the 62 phosphopeptides.
3. New Fig. 1H. We included a list of the 62 statistically significant phosphopeptides. A complete list with

the statistical parameters can be found in new Additional file 7.
4. The list of common phosphopeptides and phosphoproteins from Fig. 1F and Fig. 1G (venn diagrams) can be found in new Additional file 6.
5. New Figure 2. The Motif-X analysis was done using the 62 statistically significant phosphopeptides. The new phosphosites and statistics are depicted in Fig. 2A-B. We could identify the S/TP consensus site from Pro-directed kinases; the S-x-x-x-x-P containing the S-x-x-x-S/T consensus site from GSK3 kinases and the S-x-x-E motif described for Polo-kinases and casein kinase II (S-x-x-D/E). Therefore, our conclusion that PP2A-Cdc55 counteracts other kinases in addition to Cdk1 is still supported by the data.
6. New Fig. 2C. The comparison with the Cdk1 substrates published was done using the 55 statistically significant phosphoproteins (corresponding to the 62 phosphopeptides). A subset of 20 common proteins was identified, being common hits as Cdk1 and PP2A-Cdc55 targets.
7. The GO analysis was made using the 55 phosphoproteins (new Table 1). An enrichment on actin-cytoskeleton terms was observed. In order to extend our analysis we also performed the GO (new Additional file 8 and new Table 2) and the String (new Figure 3 and Additional file 9, 10) analysis with the 247 proteins identified in at least 2 phosphoenrichment methods (common proteins from Fig. 1G, venn diagrams).
8. New Additional file 13. The list of peptides containing a D/E/N-x-S/T Cdc5 consensus sites within the 62 phosphopeptides.
9. New Additional file 14. Comparison with the published data from Godfrey et al was also done using the 55 significant phosphoproteins.

Close
