# Supplementary material for: SILAC-based phosphoproteomics reveals new PP2A-Cdc55-regulated processes in budding yeast
Source: Gigascience. 2018 May 24;7(5):giy047. doi: 10.1093/gigascience/giy047 (PMC5967524; doi:10.1093/gigascience/giy047)
Supplement: Reviewer_1_Report_(Original_Submission) -- Andrew Burgess — 10/12/2017 Reviewed [file giy047_reviewer_1_report_(original_submission).pdf]

Reviewer Report

Title: SILAC-based phosphoproteomics reveals new PP2A-Cdc55-regulated processes in budding yeast.

Version: Original Submission **Date:** 10/12/2017

Reviewer name: Andrew Burgess

Reviewer Comments to Author:

The manuscript by Baro et al, describes the use of 3 different SILAC based proteomic approaches combined with a powerful yeast genetic deletion model. The goal of the project was to identify possible substrates of the PP2A-B55/cdc55 phosphatase. To do this the authors arrested wild-type and Cdc55 deletion mutants in metaphase by also deleting cdc20. The only issue with this approach is that during metaphase PP2A-cdc55 activity is suppressed and the majority of all phosphorylation sites would already be maximally phosphorylated (Olsen et al., 2010). Hence the ability to further enhance phosphorylation during this time is likely to be limited and therefore the increase observed is likely a under representation of the true number of PP2A specific phosphosites. This likely explains why only 27 sites were specifically increased, while 62 were decreased (Fig 1H).
To properly assess PP2A-Cdc55 substrates the ideal experiment would be to analyse the kinetics of dephosphorylation for the wt and deleted strains as they exit mitosis, similar to previous studies (Bouchoux and Uhlmann, 2011; Cundell et al., 2016). This may also explain why Mob1, which the authors previously showed to be a PP2A substrate, did not show up as a strong and significant hit in there SILAC approach. Also without the kinetic data, it is difficult to deconvolve the direct and indirect effects of PP2A deletion. It's possible that many of the increased phosphorylation events could be due to disruption of negative and positive feedback loops that impinge on other phosphatases and kinases (Grallert et al., 2014). It is also unclear exactly how 'increased' phosphopeptides were chosed and how these were subsequently analysed and compared especially with regards to the precise number of unique phosphosites that are specifically increased between PP2A wt and deletion strains. Despite these concerns, there is still some interesting data which could form the basis of an interesting publication. The authors should take the above limitations into account, and alter their conclusions accordingly. They would also benefit to rethink exactly what the experiment they have performed is actually telling them, and perhaps consider revising the initial goal to better suit the experimental data.

Specific points:

1. The introduction is a bit long and could be shortened, also suggest moving the discussion about previous mass spec studies to the discussion.

2. Page 4 Line 87: Perhaps consider citing either the Hunt or Castro labs for the role of Greatwall in regulating PP2A. (see 2010 Science papers).

3. Page5 Lin105-7: "PP2ACdc55 downregulation in anaphase": I assume the authors mean increased activity here as PP2A is reactivated during mitotic exit not repressed.

4. Figure 1A: It would be nice to have a diagram here where you show the work flow. Specifically highlighting the Wt and deleted strains being compared, which one was grown in which media (Heavy or Light), number of biological replicates etc .as I think this may help readers understand what is being compared. You could condense and simplify additional figure 1 and insert it here perhaps?

5. Page 8 Line 181: Some greater clarification about the number of phosphopeptides that are increased in the deletion mutant are needed. From Figure 1H it appears that there are only 27 sites. But the text suggest that this number is 1,260. I assume that 1A is the whole dataset, that is 1260 sites were

identified across both the wt and del mutant? If so Fig1H is the most important figure and the analysis needs to be primarily performed on the 26 sites that are increased compared to the wt. Not the complete 1260.

6. Additional File 2: What are the values listed? Are these are log2 H/L ratios as they are all below 1? A full table with the H/L ratios for all identified phosphopeptides along with the p-value would be very helpful. In addition, there should be a comparisons between biological replicates along with p-values to ensure that sites identified are significant. Were label-swaps performed? This data should be presented in a single clearly labelled table.

7. It appears the data has been heavily averaged, and I couldn't quite follow what was done in order to generate Figure 1A. Why was a H/L ratio of less than 0.75 considered to be increased phosphorylation? Normally a ratio greater than would be the desired cut-off would be used (e.g. >1 for a 2-fold increase). Similarly, a value <-1 would be considered significantly decreased with values between -1 and +1 considered unchanged. These need to clarified

8. From Figure 1H it appears that only 27 or 62 phosphosites are increased in the deleted vs wt strains. If this is correct, then the analysis should be performed on these 27/62 phosphosites, not the full dataset. This goes for the rest of the analysis, such as the STRING, motif-x and GO-enrichments. This also seems to contradict Figure1A, can the authors explain this better?

9. Page 10 Line 233: 562 unique motifs seem very high especially for an input of 721 peptides, it's almost 1:1, and above the number of total kinases in yeast. Also, the authors should clarify exactly which phosphopeptides were used for the analysis. Again if they want to show PP2A specific effects then only the phosphopeptides that are specifically increased in the deletion vs wt should be analysed.

10. Page 11: "Novel roles for PP2ACc55 phosphatase in cytokinesis and endocytosis".
A 'd' is missing in the title. Also, I think a bit too much is made of the GO-terms. These are most likely reflecting the fact that you have mitotically arrested samples and you cannot conclude that they are PP2A specific, without doing additional assays. It is also not clear what is being compared. The most logical thing here would be to look for differences between the wt and deleted strains, but this doesn't appear to be what was done. Rather, all phosphopeptides identified were analysed. If this is the case, then the analysis is likely only reflecting the mitotic state, and not able to specifically tell you anything about PP2A substrates.

11. Figure 4: A non-PP2A substrate should be included to show that any disruption to the timing of mitotic exit potentially caused by PP2A-Cdc55 deletion is not affecting dephosphorylation kinetics. Also timing here would be more appropriate label rather than cell cycle stage, which is subjective. Would also consider aligning all of the blots above and below each other. The phosphatase assay should be labelled as a separate panel (e.g. 4C), and a loading control is needed.

References:
Bouchoux, C., Uhlmann, F., 2011. A Quantitative Model for Ordered Cdk Substrate Dephosphorylation during Mitotic Exit. Cell 147, 803-814. doi:10.1016/j.cell.2011.09.047
Cundell, M.J., Hutter, L.H., Bastos, R.N., Poser, E., Holder, J., Mohammed, S., Novák, B., Barr, F.A., 2016. A PP2A-B55 recognition signal controls substrate dephosphorylation kinetics during mitotic exit. J. Cell Biol. 257, jcb.201606033-554. doi:10.1083/jcb.201606033
Grallert, A., Boke, E., Hagting, A., Hodgson, B., Connolly, Y., Griffiths, J.R., Smith, D.L., Pines, J., Hagan, I.M., 2014. A PP1-PP2A phosphatase relay controls mitotic progression. Nature 517, 94-98. doi:10.1038/nature14019
Olsen, J.V., Vermeulen, M., Santamaria, A., Kumar, C., Miller, M.L., Jensen, L.J., Gnad, F., Cox, J., Jensen, T.S., Nigg, E.A., Brunak, S., Mann, M., 2010. Quantitative phosphoproteomics reveals widespread full phosphorylation site occupancy during mitosis. Sci Signal 3, ra3-ra3. doi:10.1126/scisignal.2000475

Methods

Are the methods appropriate to the aims of the study, are they well described, and are necessary controls included? No

Conclusions

Are the conclusions adequately supported by the data shown? No

Reporting Standards

Does the manuscript adhere to the journal's guidelines on minimum standards of reporting? No

Choose an item.

Statistics

Are you able to assess all statistics in the manuscript, including the appropriateness of statistical tests used? No, and I do not feel adequately qualified to assess the statistics.

Quality of Written English

Please indicate the quality of language in the manuscript: Needs some language corrections before being published

Declaration of Competing Interests

Please complete a declaration of competing interests, considering the following questions:

- Have you in the past five years received reimbursements, fees, funding, or salary from an organisation that may in any way gain or lose financially from the publication of this manuscript, either now or in the future?
- Do you hold any stocks or shares in an organisation that may in any way gain or lose financially from the publication of this manuscript, either now or in the future?
- Do you hold or are you currently applying for any patents relating to the content of the manuscript?
- Have you received reimbursements, fees, funding, or salary from an organization that holds or has applied for patents relating to the content of the manuscript?
- Do you have any other financial competing interests?
- Do you have any non-financial competing interests in relation to this paper?

If you can answer no to all of the above, write 'I declare that I have no competing interests' below. If your reply is yes to any, please give details below.

I declare that I have no competing interests

I agree to the open peer review policy of the journal. I understand that my name will be included on my report to the authors and, if the manuscript is accepted for publication, my named report including any attachments I upload will be posted on the website along with the authors' responses. I agree for my report to be made available under an Open Access Creative Commons CC-BY license (http://creativecommons.org/licenses/by/4.0/). I understand that any comments which I do not wish to be included in my named report can be included as confidential comments to the editors, which will not be published.

I agree to the open peer review policy of the journal

To further support our reviewers, we have joined with Publons, where you can gain additional credit to further highlight your hard work (see: https://publons.com/journal/530/gigascience). On publication of this paper, your review will be automatically added to Publons, you can then choose whether or not to claim your Publons credit. I understand this statement.

Yes Choose an item.
