# Supplementary material for: SILAC-based phosphoproteomics reveals new PP2A-Cdc55-regulated processes in budding yeast
Source: Gigascience. 2018 May 24;7(5):giy047. doi: 10.1093/gigascience/giy047 (PMC5967524; doi:10.1093/gigascience/giy047)
Supplement: Reviewer_2_Report_(Original_Submission) -- Peter Li — 10/17/2017 Reviewed [file giy047_reviewer_2_report_(original_submission).pdf]

Reviewer Report

Title: SILAC-based phosphoproteomics reveals new PP2A-Cdc55-regulated processes in budding yeast.

Version: Original Submission **Date:** 10/17/2017

Reviewer name: Peter Li

Reviewer Comments to Author:

The authors report on the identification of new substrates for PP2A Cdc55 phosphatase during mitosis using mass spectrometry by comparing the phosphoproteome of a wild type yeast strain to that for a cdc55 lacking mutant strain. The manuscript is well-written and appears comprehensive in reporting how the current literature relates to their results on PP2A Cdc55 phosphatase substrates.

Since I only have a layman's knowledge of proteomics and cell cycle signalling pathways, the main aim of this review is to check the consistency of the data supporting the results reported in the manuscript by the authors. In general, I feel Baro et al have been comprehensive in disseminating the data associated with their manuscript and I have only minor suggestions for improvements.

The manuscript requires an, "Availability of supporting data and materials" section. In this section, information about the raw data stored in PRIDE should be provided.

With regards to the data itself, the dataset contains *.raw files which have been generated by the Orbitrap mass spectrometer. The dataset also contains *.msf files - what are these?

I think it would be helpful if the dataset provides information on the files it contains such as what they are and which experiments they correspond to. For example, are the files from the large-scale identification of PP2A Cdc55 -dependent phosphoproteome work, TAP- or HA-tagging experiments? Is it possible to add this information into their PRIDE archive dataset entry webpage or in a README text file that is added to their dataset?

Large-scale identification of PP2ACdc55-dependent phosphoproteome in metaphase-arrested cells

Can the authors check that Fig. 1C is referred to in the manuscript text?

The list of peptides listed in PDF files in Additional files 3 and 4 could be provided in an Excel file or text format. This will aid the re-use of the data instead of users having to copy and paste or even type out them manually if the copying function does not work properly in their PDF document applications.

Novel roles for PP2A Cdc55 phosphatase in cytokinesis and endocytosis

Are the protein interactions of the network shown in Additional file 7 available as a text file as well as a screenshot? This would be more useful if people wanted to re-use it.

Validation of novel PP2A cdc55 substrates in mitosis

Page 15 first paragraph: Is the reference to Additional file 1 correct on line 337? Maybe Additional file 3 is the correct file to be referred to? If so, where is Kin4 in this file?

Data Analysis for peptide identification and quantification

This Section mentions the use of R with the rvest, vennerable, venneuler packages - page 27 line 640. The R scripts should be provided along with documentation on how to use it. Since R scripts are source code then the manuscript will need an, "Availability of source code and requirements" section as detailed in the authors instructions (https://academic.oup.com/gigascience//pages/research).

Structure prediction of Cdc55

I do not have experience of the tools used in this section. Are HHpred and MODELLER command line tools? If so, then any scripts involving their use should be make available.

Sampling the binding interface of the CDC55/Mob1 complex
If HADDOCK is a command line tool then any script involving its use should be made available.

--

Peter Li, GigaScience

Methods

Are the methods appropriate to the aims of the study, are they well described, and are necessary controls included? Yes

Conclusions

Are the conclusions adequately supported by the data shown? Yes

Reporting Standards

Does the manuscript adhere to the journal's guidelines on [minimum standards of reporting?]() Yes

Choose an item.

Statistics

Are you able to assess all statistics in the manuscript, including the appropriateness of statistical tests used? No, and I do not feel adequately qualified to assess the statistics.

Quality of Written English

Please indicate the quality of language in the manuscript: Acceptable

Declaration of Competing Interests

Please complete a declaration of competing interests, considering the following questions:

- Have you in the past five years received reimbursements, fees, funding, or salary from an organisation that may in any way gain or lose financially from the publication of this manuscript, either now or in the future?
- Do you hold any stocks or shares in an organisation that may in any way gain or lose financially from the publication of this manuscript, either now or in the future?
- Do you hold or are you currently applying for any patents relating to the content of the manuscript?
- Have you received reimbursements, fees, funding, or salary from an organization that holds or has applied for patents relating to the content of the manuscript?
- Do you have any other financial competing interests?
- Do you have any non-financial competing interests in relation to this paper?

If you can answer no to all of the above, write 'I declare that I have no competing interests' below. If your reply is yes to any, please give details below.

I am a paid employee of GigaScience.

I agree to the open peer review policy of the journal. I understand that my name will be included on my report to the authors and, if the manuscript is accepted for publication, my named report including any attachments I upload will be posted on the website along with the authors' responses. I agree for my report to be made available under an Open Access Creative Commons CC-BY license (http://creativecommons.org/licenses/by/4.0/). I understand that any comments which I do not wish to be included in my named report can be included as confidential comments to the editors, which will not be published.

I agree to the open peer review policy of the journal

To further support our reviewers, we have joined with Publons, where you can gain additional credit to further highlight your hard work (see: https://publons.com/journal/530/gigascience). On publication of this paper, your review will be automatically added to Publons, you can then choose whether or not to claim your Publons credit. I understand this statement.

Yes Choose an item.
