# Supplementary material for: SILAC-based phosphoproteomics reveals new PP2A-Cdc55-regulated processes in budding yeast
Source: Gigascience. 2018 May 24;7(5):giy047. doi: 10.1093/gigascience/giy047 (PMC5967524; doi:10.1093/gigascience/giy047)
Supplement: Reviewer_2_Report_(Revision_1) -- Peter Li — 1/22/2018 Reviewed [file giy047_reviewer_2_report_(revision_1).pdf]

Reviewer Report

Title: SILAC-based phosphoproteomics reveals new PP2A-Cdc55-regulated processes in budding yeast.

Version: Revision 1 **Date:** 1/22/2018

Reviewer name: Peter Li

Reviewer Comments to Author:

I am satisfied that the authors have addressed all my points in my first review. However, the R scripts in Additional file 15.doc should all be uploaded into a GitHub (https://github.com) repository with documentation on their execution if this manuscript is accepted for publication. The technical team at GigaScience can assist with this if required.

Methods

Are the methods appropriate to the aims of the study, are they well described, and are necessary controls included? Yes

Conclusions

Are the conclusions adequately supported by the data shown? Yes

Reporting Standards

Does the manuscript adhere to the journal's guidelines on minimum standards of reporting? Yes

Choose an item.

Statistics

Are you able to assess all statistics in the manuscript, including the appropriateness of statistical tests used? No, and I do not feel adequately qualified to assess the statistics.

Quality of Written English

Please indicate the quality of language in the manuscript: Acceptable

Declaration of Competing Interests

Please complete a declaration of competing interests, considering the following questions:

- Have you in the past five years received reimbursements, fees, funding, or salary from an organisation that may in any way gain or lose financially from the publication of this manuscript, either now or in the future?

- Do you hold any stocks or shares in an organisation that may in any way gain or lose financially from the publication of this manuscript, either now or in the future?
- Do you hold or are you currently applying for any patents relating to the content of the manuscript?
- Have you received reimbursements, fees, funding, or salary from an organization that holds or has applied for patents relating to the content of the manuscript?
- Do you have any other financial competing interests?
- Do you have any non-financial competing interests in relation to this paper?

If you can answer no to all of the above, write 'I declare that I have no competing interests' below. If your reply is yes to any, please give details below.

I am a salaried employee of GigaScience.

I agree to the open peer review policy of the journal. I understand that my name will be included on my report to the authors and, if the manuscript is accepted for publication, my named report including any attachments I upload will be posted on the website along with the authors' responses. I agree for my report to be made available under an Open Access Creative Commons CC-BY license (http://creativecommons.org/licenses/by/4.0/). I understand that any comments which I do not wish to be included in my named report can be included as confidential comments to the editors, which will not be published.

I agree to the open peer review policy of the journal

To further support our reviewers, we have joined with Publons, where you can gain additional credit to further highlight your hard work (see: https://publons.com/journal/530/gigascience). On publication of this paper, your review will be automatically added to Publons, you can then choose whether or not to claim your Publons credit. I understand this statement.

Yes Choose an item.
