# Supplementary material for: SILAC-based phosphoproteomics reveals new PP2A-Cdc55-regulated processes in budding yeast
Source: Gigascience. 2018 May 24;7(5):giy047. doi: 10.1093/gigascience/giy047 (PMC5967524; doi:10.1093/gigascience/giy047)
Supplement: Reviewer_3_Report_(Original_Submission) -- Arminja Kettenbach — 10/18/2017 Reviewed [file giy047_reviewer_3_report_(original_submission).pdf]

Reviewer Report

Title: SILAC-based phosphoproteomics reveals new PP2A-Cdc55-regulated processes in budding yeast.

Version: Original Submission **Date:** 10/18/2017

Reviewer name: Arminja Kettenbach

Reviewer Comments to Author:

Baro et al conducted an analysis of PP2A-Cdc55 substrates and interacting proteins under mitotic arrest conditions in budding yeast by proteomics and quantitative phosphoproteomics. They validated differences in phosphorylation for proteins involved in the MEN and FEAR pathway as well as a different PP2A regulatory subunit, and generated a docking model for Cdc55 and Mob1.

PP2A is a phosphoprotein phosphatase consisting of catalytic, scaffolding, and regulatory subunit. The regulatory subunit confers substrate specificity. The PP2A regulatory subunit Cdc55 is the yeast homolog of B55 in humans and thought to be responsible for counteracting Cdk1 phosphorylation during mitotic exit. In yeast, PP2A-Cdc55 acts in concert with the Cdc14 phosphatase during mitotic exit.

The authors indicate that while their study was under preparation, another study was published that conducted PP2A-Cdc55 substrate analyses in G1, S, and G2. A comparison of the datasets should be included.

Baro et al uses a Cdc55-delete strain to identify potential substrates of PP2A-Cdc55 in mitotic arrest which is induced by Cdc20 depletion. The authors state that 95% of cells were arrested in metaphase but do not show data to support this. This should be included.

To detect differences in phosphorylation between wild-type and Cdc55-delete strains, the authors use a SLIAC strategy. It would be helpful if the authors could include a scheme of the experimental design and clearly indicate which strain was labeled heavy and which strain was labeled light.

Interestingly, the authors used three different strategies for phosphopeptide enrichment but do not provide an explanation for this unusual combination. Please explain.

A table of all identified peptides or at least phosphopeptides is not included, please do so.

In their analyses, they identify 1,260 phosphorylation sites that increase in phosphorylation upon Cdc55 deletion. While a table of hyperphosphorylated sites is included, the description of this table (additional file 2) needs to be improved to be understandable for the reader. Are the values in the TiSh, SIMAC, TiO2 column ratios? If so of what? The statistical analysis is missing. It would also be helpful if either the site of phosphorylation would be indicated in the peptide sequence in column c or if the peptide sequence would be represent as centered around the phosphorylated residue +/- 6 amino acid for instance. P-values for the quantification of each phosphopeptides should be included. Only the hyperphosphorylated peptides are included in additional file 2, however, the volcano plot in Figure 1H shows all data. This data needs to be added.

To exclude that differences detected in phosphorylation are due to do difference in protein abundance, the authors conduct an analysis of protein abundance differences in both strains. However, the authors do not conduct a subtractive analysis to correct phosphopeptide ratios by protein abundance differences, just state that most proteins don't change. Such an analysis should be included. The identification and quantification results for the protein analysis are not included. Only a list of protein names is provided in additional file 3. The complete list of all quantified protein ratios and their identification metrics needs to be added.

Only 145 sites are identified in 2 out of 3 replicates and only 30 in all three replicates. The low reproducibility of identifications and quantifications is potentially due to the use of three different enrichment methods, each of which likely selects for a different subset of phosphopeptides. Because the overlap of the three or even two out of three datasets is very low, subsequent analyses based on phosphopeptides that are identified as statistically significantly increased are limited. To improve the dataset, the authors need to improve the overlap of the phosphoproteomic analyses for instance by using the same phosphopeptide enrichment strategy in replicate to ensure greater reproducibility in phosphopeptide identification and

quantification.
Instead of concentrating on the 62 phosphopeptides with statistically significant increases in phosphorylation, the authors conduct their subsequent analyses on all increased phosphorylation sites. Because the abundance change of these phosphopeptides is not supported by statistical analysis, these analyses are not informative. For instance, the authors state that they find a preference of PP2A-Cdc55 for the dephosphorylation of phosphothreonine over phosphoserines as reported by others. However, the enrichment in threonine-directed phosphorylation sites is not observed in just the 62 statistically significant increased phosphorylation sites. The same strategy of using all increased sites is applied for all subsequent analysis, including assigning counteracting kinases, GO and String network analysis, and needs to be revised using a dataset with a higher degree of overlap in phosphopeptide identifications and quantifications.
The authors also perform Cdc55 interactome studies. Again only protein names are provided in additional file 8, no information on peptide counts versus controls or parameters/metrics of identification are provided. Surprisingly, for the analysis of HA-tagged Cdc55 this information is available in additional file 9. Here the authors perform two replicates, however, it is not determined if the identifications were reproducible. There is no explanation why the authors performed TAP as well as HA purifications. The authors concluded that the interactomes analysis in total are not informative because only a few proteins identified in the phosphoprotomics analysis are captured here.
Finally, the authors perform an in silico analysis of Cdc55 and Mob1 to identify interaction surfaces which recapitulates a previously identified interface on Cdc55 but does not identify a corresponding interface on Mob1.
In general the identification of PP2A holoenzyme specific substrates is of high interest and could be a great resource for the community. However, the analyses conducted here lack rigor and need to be improved upon to be valuable.

Methods

Are the methods appropriate to the aims of the study, are they well described, and are necessary controls included? No

Conclusions

Are the conclusions adequately supported by the data shown? No

Reporting Standards

Does the manuscript adhere to the journal’s guidelines on minimum standards of reporting? Yes

Choose an item.

Statistics

Are you able to assess all statistics in the manuscript, including the appropriateness of statistical tests used? Yes, and I have assessed the statistics in my report.

Quality of Written English

Please indicate the quality of language in the manuscript: Acceptable

Declaration of Competing Interests

Please complete a declaration of competing interests, considering the following questions:

- Have you in the past five years received reimbursements, fees, funding, or salary from an organisation that may in any way gain or lose financially from the publication of this manuscript, either now or in the future?
- Do you hold any stocks or shares in an organisation that may in any way gain or lose financially from the publication of this manuscript, either now or in the future?
- Do you hold or are you currently applying for any patents relating to the content of the manuscript?
- Have you received reimbursements, fees, funding, or salary from an organization that holds or has applied for patents relating to the content of the manuscript?
- Do you have any other financial competing interests?
- Do you have any non-financial competing interests in relation to this paper?

If you can answer no to all of the above, write 'I declare that I have no competing interests' below. If your reply is yes to any, please give details below.

No competing interests

I agree to the open peer review policy of the journal. I understand that my name will be included on my report to the authors and, if the manuscript is accepted for publication, my named report including any attachments I upload will be posted on the website along with the authors' responses. I agree for my report to be made available under an Open Access Creative Commons CC-BY license (http://creativecommons.org/licenses/by/4.0/). I understand that any comments which I do not wish to be included in my named report can be included as confidential comments to the editors, which will not be published.

I agree to the open peer review policy of the journal

To further support our reviewers, we have joined with Publons, where you can gain additional credit to further highlight your hard work (see: https://publons.com/journal/530/gigascience). On publication of this paper, your review will be automatically added to Publons, you can then choose whether or not to claim your Publons credit. I understand this statement.

Yes Choose an item.
